# Supplementary material for: Gaps and opportunities in modelling human influence on species distributions in the Anthropocene
Source: Nat Ecol Evol. 2024 Jun 12;8(7):1365–77. doi: 10.1038/s41559-024-02435-3 (PMC11239511; doi:10.1038/s41559-024-02435-3)
Supplement: Supplementary file 1 — Supplementary Figs. 1 and 2 and Tables 1–4. [file 41559_2024_2435_MOESM1_ESM.pdf]

nature ecology & evolution

Supplementary information https://doi.org/10.1038/s41559-024-02435-3

Gaps and opportunities in modelling human influence on species distributions in the Anthropocene

In the format provided by the authors and unedited

Contents

Supplementary Figure 1. Variation in human and environmental predictor use by domain 2
Supplementary Figure 2. Human predictor selection and Sustainable Development Goals 3
Supplementary Table 1. Abstract screening iterations and dictionary of terms 4
Supplementary Table 2. Systematic review data field descriptions 12
Supplementary Table 3. Qualitative evaluation summary of human predictor performance 18
Supplementary Table 4. List of human predictors used in SDM studies 19

Supplementary Figure 1. Variation in human and environmental predictor use by domain

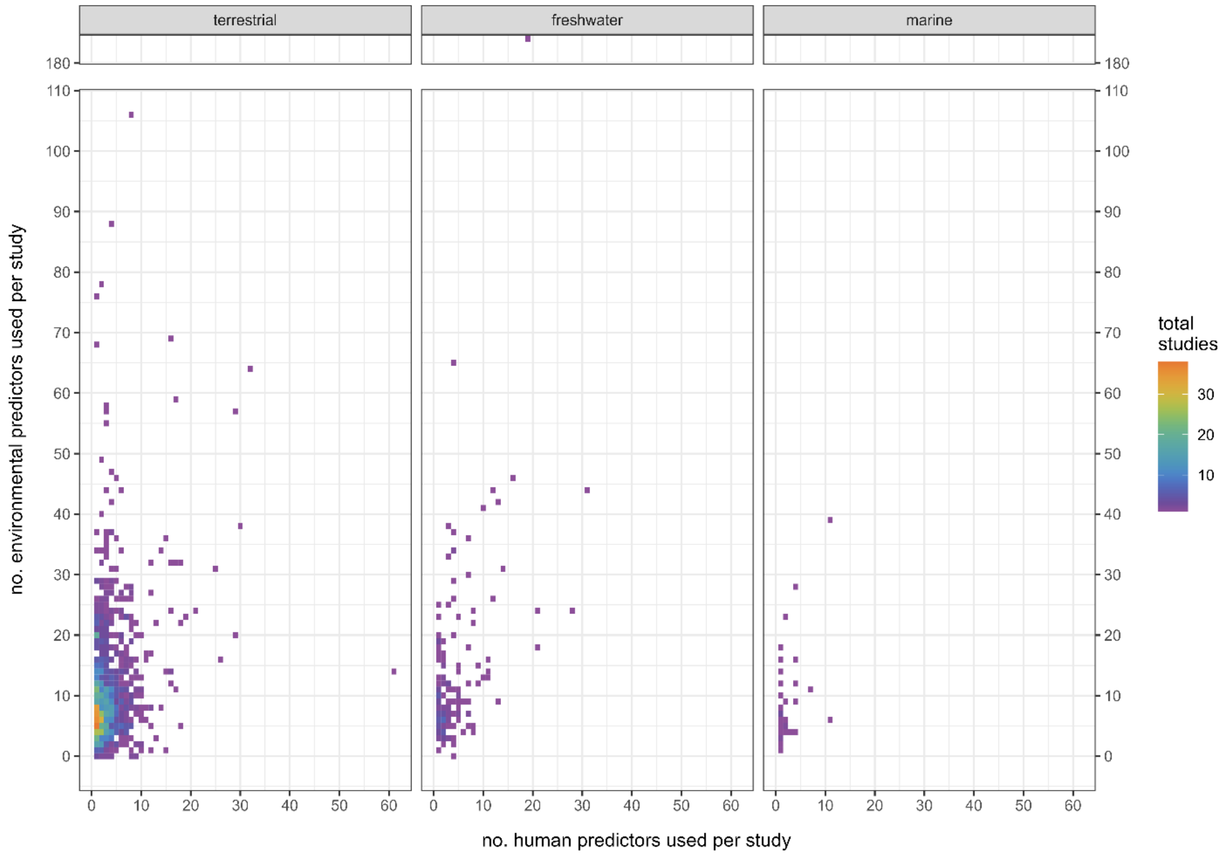

Supplementary Figure 1. Comparison of human and environmental predictor use in SDM training across terrestrial, freshwater, and marine domains, and the frequency of their use across all studies from the 1,429 accepted full articles. In terrestrial and marine studies, as many as 10 human predictors are typically used alongside environmental predictors, while the number of environmental predictors varied greatly. In marine studies, there are very few human predictors used compared to environmental predictors.

Supplementary Figure 2. Human predictor selection and Sustainable Development Goals

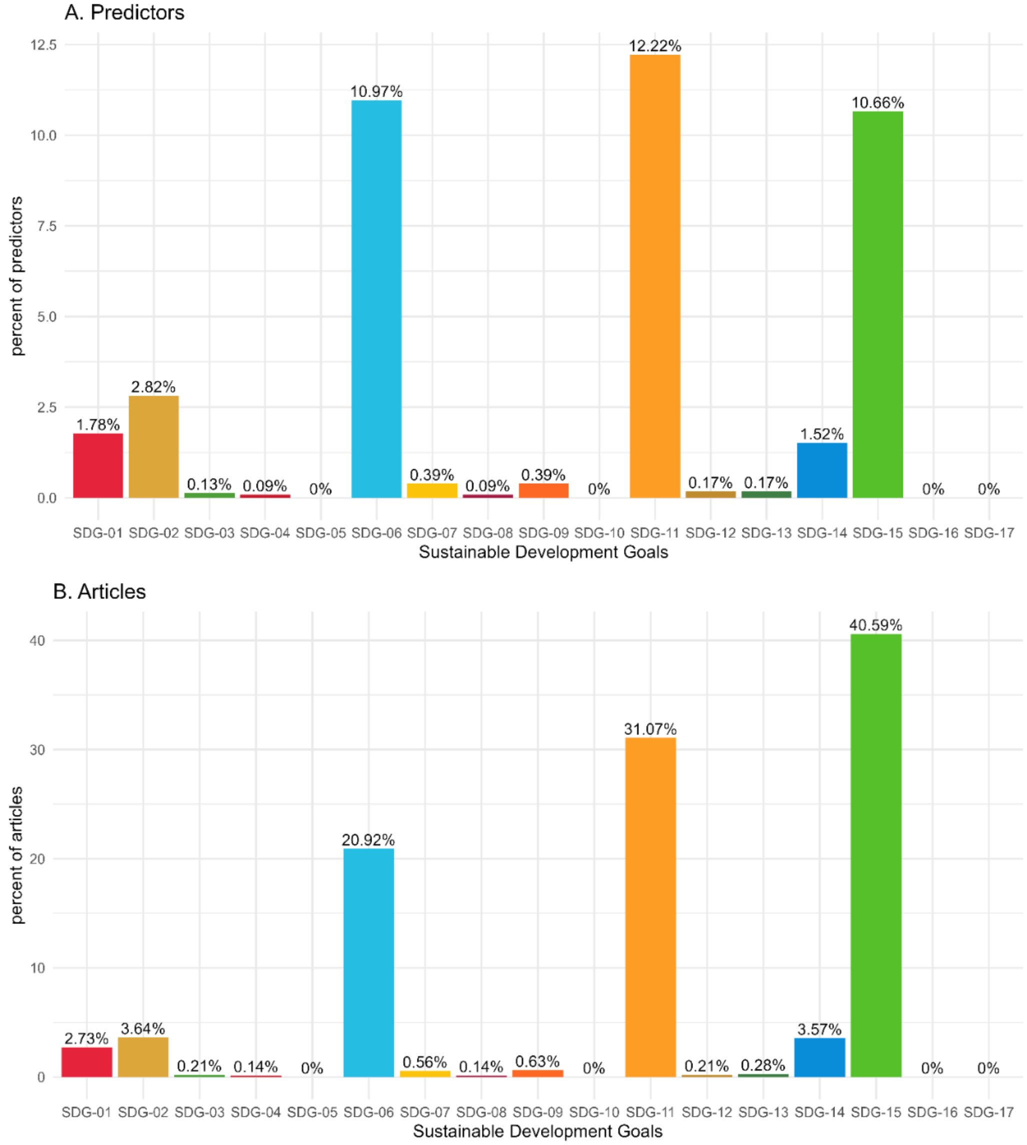

Supplementary Figure 2. Human predictors used in SDMs are more closely related to Life on Land (SDG-15), Sustainable Cities and Communities (SDG-11), and Clean Water and Sanitation (SDG-06). This was seen both for frequency of predictors related to SDGs (A), and the number of articles using those predictors (B). A total of 682 of the 2,307 human predictors related to SDGs. They were used by 924 of 1,429 articles (65%). There were no predictors related to Gender Equality (SDG-05), Reduced Inequality (SDG-10), Peace and Justice Strong Institutions (SDG-16), and Partnerships to Achieve the Goal (SDG-17). *Abbreviations: SDG-01: No Poverty; SDG-02: Zero Hunger; SDG-03: Good Health and Well-being; SDG-04: Quality Education; SDG-05: Gender Equality; SDG-06: Clean Water and Sanitation; SDG-07: Affordable and Clean Energy; SDG-08: Decent Work and Economic Growth; SDG-09: Industry, Innovation and Infrastructure; SDG-10: Reduced Inequality; SDG-11: Sustainable Cities and Communities; SDG-12: Responsible Consumption and Production; SDG-13: Climate Action; SDG-14: Life Below Water; SDG-15: Life on Land; SDG-16: Peace and Justice Strong Institutions ; SDG-17: Partnerships for the Goals).

Supplementary Table 1. Abstract screening iterations and dictionary of terms

Supplementary Table 1. Description of the abstract screening steps for 12,683 articles on SDMs. Over the course of 28 iterations, ~300 abstracts were reviewed at a time and manually accepted or rejected (see Extended Data Fig. 1 and methods for acceptance criteria). Then, for the articles that were accepted, any key words or phrases that the authors used to describe human influence on species' distributions were recorded, along with any potential synonyms. Assuming that any other articles using the same key terms would be equally relevant for our synthesis, we used the key words from each respective screening iteration to scan the entire pool of abstracts and accept additional, similar articles. This process was repeated 28 times, yielding 8,320 abstracts manually screened and 551 abstracts manually accepted, leading to 477 key words and phrases being recorded from the author abstracts, from which 4,626 abstracts using those terms were accepted. In total, we accepted 5,177 article abstracts for the full article screening step. Of note, all rejected abstracts were manually screened, as no search terms were used to automatically reject them.

iteration	no. screened	no. accepted	no. rejected	keywords added	no. new keywords	no. pooled from key-words	cum. total accepted	cum. total rejected
**1**	301	122	179	"agricultur*; aquacultur*; hunting; viticultur*; hunted; plantation*; livestock; game bird*; palm oil; cropland; anthropogenically-transformed; urban*; anthropogenic impact*; human impact*; pollutant*; insecticide*; pesticide*; land abandonment; deforest*; landuse change; land use change; landcover change; land cover change; habitat conversion; land conversion; habitat degradation; oil spill*; collision; human-wildlife conflict; human interaction*; road kill; roadkill; artificial surface*; hydropower; artificial light; artificial night light"	36	2133	2255	168
**2**	289	49	240	"anthropogenic change; human use; anthropogenic activit*; shipping; anthropogenic global change; human activit*; human population; anthropogenic stressor*; human footprint*; anthropogenic driver*; human pressure*; synanthrop*; anthropic	24	755	3059	404

				intervention; anthropogenic cause*; anthropogenic habitat; human-dominated; exploitation; exploited; domestic animal*; highway*; ^mines; reintroduc*; relocat*; translocat*"				
**3**	300	40	260	"fishing; human water use; cattle; whaling; market demand*; anthropic pressure*; harvest*; aquarium; grazing; anthropogenic pressure*; global demand; ^logging; reservoir*; land use; globalization; globalisation; land cover; landcover; distance from village*; anthropogenic variable*; reclamation; human development; anthropogenic litter; land use change*; entanglement; wind energy development; human persecution; habitat encroachment; developed landscape*"	29	851	3950	634
**4**	301	32	269	"human related activit*; human-related activit*; human-mediated; farmland; anthropogenic and; anthropogenic ecological change*; human settlement*; village; villages; anthropogenic expansion*; human infrastructure*; land-use change*; abandoned field*; abandoned area*; developed area*"	15	125	4107	901
**5**	302	27	275	"rice field*; public land; public use*; human influence index; closeness to ports; domestic pig census; human-related change*; land-use data; land use data; land cover data; landcover data; human-altered landscape*; built environment*; built-up area; habitat destruction; land-use variable*; human-caused habitat loss; human-induced global environmental change; human-carnivore conflict*; ^city; ^cities; effectiveness of protected areas; effectiveness of PA; eradicat*;	28	98	4232	1166

				forest management practice*; ability of PAs to conserve; park boundar*; nonreserved area*"				
**6**	300	17	283	"crop area*; human-induced variables; land-use realism; Socio-Economic Connectedness; human behavior; disturbed environment*; ski slope*; clear-cut logging; human modification; abandoned land*; abandonment; ^mining activit*; Human-sun bear conflict; human propert*; conflict area*; conflict hotspot*; shelterbelt*; human-made habitats; farmstead"	19	14	4263	1447
**7**	300	22	278	"abstraction; demand for; commercial logging; human and natural variable*; human and natural predictor*; landscape-Use; land-use pattern; anthropogenic factor*; land-cover; natural *land*; National Land Cover Data; *NLCD*; forest availability; oil development; gas development; *restored habitat; human-caused; distance to anthropogenic; density of vehicle roads; road density; density of roads; avoid* road*; road* avoid*; avoid* of road*; avoid* of trail*; avoid* trail*; attract* to road*; attract* to trail*; undeveloped; human intervention*"	30	150	4435	1710
**8**	301	16	285	"timber production; social value*; ballast; ^cities; orchard*; pasture*; human-induced; companies that import; imported by compan*; company import*; companies import; human occup*; land-use; human-pressure*; landscape chang*; chang* landscape*; poach*; access* roads; access to road*; road* access*; industrial cent*; power development; wind energy; anthropogenic structure*"	24	109	4560	1967

**9**	300	13	287	"anthropogenic* introduc*; forest edge*; changes that human*; changes that people; human influence*; anthropogenic environmental change*; human incidence*; international travel; freight transport*; pitlake; ^pit lake; anthropogenic stress; human-made; roadside; tourism development"	15	26	4599	2250
**10**	300	11	289	"anthropo* parameter*; anthropo* feature*; pollution; pollutant*; ^escape*; air traffic; distance* from road*; distance* from *trail*; distance* from highway*; distance* from settlement*; distance* from village*; fisher* bycatch; ship strike*; anthro* sound*; anthropo* *nourishment; renourishment event; anthropic restoration; uninterrupt* area*; forest thinning; thin* forest*; habitat management; manag* habitat*"	22	73	4683	2524
**11**	300	7	293	"shade tree*; coffee farm*; anthrop* dominated; anthrop*-dominated; human dominated; human-dominated; dominated by human*; dominated by people; anthrop* indices; anthrop* index; barrier effect; main road*; vector control; manage* factor*; protect* area*"	15	51	4741	2802
**12**	300	11	289	"widely traded; widely-traded; dam removal.*; and anthropogenic\\.; human* interfer.*; N emission.*; road mortality; road-kill.*; wind farm.*; rely on intensive manage.*; rely on manage.*; habitat.* alteration; habitat change.* from restoration"	13	60	4812	3064
**13**	300	13	287	"fishery catch; accessible to human.*; anthropogenic predictor.*; anthropogenic parameter.*; anthropogenic extinction; anthropogenic feature.*; demand.* for timber;	21	10	4835	3348

				preindustrial period; industrialization; industrialisation; anthropogenic degradation; edge.* of fields; field edge.*; conflict with human.*; human safety; human disturbed; human-disturbed; disturbed by human.*; anthropo.* disturb.*; Ski-resort.*; ski resort.*"				
**14**	301	11	290	"planting area.*; intentional.* introduc.*; trade pattern.*; socioeconomic factor.*; industrial source.*; municipal source.*; anthropogenic fire.*; human induced threat.*; human-induced threat.*; threat.* by human.*; anthrop.* threat.*; non-ecological; nonecological; introduction site.*; occurs in protected areas"	15	34	4880	3626
**15**	300	13	287	"silvicultur*; socio-economic covariate.*; socioeconomic covariate.*; accidentally introduced; accidentally-introduced; unintentionally transported; transport hub; inadvertent.* introduc.*; post-mining landscape; intentional feeding; unintentional feeding; transport pressure; dam construction; ^on road.*; prescribed fire; fire suppression; euthan*"	17	29	4922	3899
**16**	300	15	285	"^hunted area; human presence; humans created niches; human economic activity; due to energy development; fishery has the potential to compete with; collector.* interest; distance to road; settlements; night-time light.*; nighttime light; artificial light; ecological impact of hydroelectric power plants; effectiveness of conservation areas; expanding protected areas; measures to protect has led to; protection measures; conservation and economic development"	18	0	4937	4184

**17**	309	20	289	"threat from fisheries; fisher.* threat; human and spatial variable.*; human and environment.* variable.*; human and spatial predictor; human and environent.* predictor; human-related variable; human related variable; human variable; human-related predictor; human related predictor; human predictor; human induced pressure; human-induced pressure; exacerbated by human; human threat.*; during ecological restoration.*; wolf (Canis lupus) restoration"	18	6	4963	4470
**18**	300	8	292	"distribution of fisher.*; species was imported; energy development variable.*; distance to oil/gas road.*; human landscape alteration; public garden.*; domestic garden.*; protected area coverage"	8	5	4976	4759
**19**	300	21	279	"olive cultivation; prevalence in vineyards; human introduc.*; biocontrol agent; anthropogenic landscape.*; anthropogenic vector.*; anthropic; role played by human.*; amount of cleared land; marine litter; microplastic; mesoplastics; macrolitter; anthropogenic modification; coincid.* with human; dive tourism; conflict.* with human.*; major harbor.*; airport.*; entry through ports; manipulated organism*; political division.*; Water Framework Directive; sensitive to forest management"	24	25	5022	5024
**20**	300	10	290	"live animal trade; non-cultivated area.*; anthropogenic process.*; anthropological change; forest cover change; seafloor dredging; coastal intervention; avoid grazed land; inhabit people's homes; biological control"	1	19	5051	5302

**21**	300	9	291	"resources by humans; 20th-century logging; human arrival; land degradation; mine edge; ^mining; ^mines; culling; culled; energy generation development; green infrastructure; fish are stocked; stocking"	13	17	5077	5581
**22**	300	8	292	"cultivation intensity; seismic survey; roads,; dyke; paved road; response to habitat restoration"	6	5	5090	5872
**23**	300	9	291	"land resource use; human access; socioecological framework; human-environment relationship; human means; arrive by human; human assisted; human-assisted; development project; pet store; powerline; restoration success; restoration design; after restoration"	14	9	5108	6157
**24**	300	10	290	"land-cover data; ^mining activit*; trawling impact; cost of conflict resolution; human-cougar conflict; resource utilization with human; co-occurrence of human; restorations strateg; restoration strateg"	9	5	5123	6442
**25**	300	11	289	"pre-clearing; introduced in cities; traded pets; socioeconomic condition; disturbed by human; distance to watercourses; restoration performance; indicators of restoration; regulated by dam; sluices; introducing species; pleistocene rewilding"	12	2	5136	6729
**26**	300	15	285	"geographic expansion of cultivated populations; anthropogenic land; socioeconomic variable; human and historic variables; impact of humans; social factor; development and population; land clearing; anthropogenic mortality; density of road; human-modified; historical land management;	16	8	5159	7008

				MPA zoning; with irrigation; without irrigation; distance to protected area"				
**27**	300	5	295	"cultivated/naturalised population; cultivated population; land transformation; human-managed; natural-like fishways"	5	1	5165	7302
**28**	216	6	210	"coffee presence; introduction risk area; maritime port; major cities; manipulated by; manipulation by; stand density; afforestation; mowing; conservation-dependent"	10	6	5177	7506

Supplementary Table 2. Systematic review data field descriptions

Supplementary Table 2. Description of data fields for the systematic review, corresponding to the data provided in Supplementary Tables 3-6.

Column name	Description	Details
**uid**	unique identification number for each article (corresponds to numbers for references listed in Supporting Information)	###
**year**	publication year	YYYY; obtained from ISI Web of Science search export
**title**	article title	text; obtained from ISI Web of Science search export
**author**	author(s)	text; obtained from ISI Web of Science search export
**journal**	journal	text; obtained from ISI Web of Science search export
**relevant**	define whether the reviewed article is a correlative species distribution model and uses human-related (anthropogenic) predictors in model training	yes, no, semi; yes for articles accepted both through the abstract and full article screening and used for synthesis in the systematic review; if no, then no further data fields are filled in for this study; semi is a way of making special note of articles that do not use human predictors within the SDM training, but human predictors are part of the study's overall assessments (e.g., post-SDM applications, masking predictions by urban areas, etc.); note that articles that solely use human predictors for e.g. sampling bias or observer effort probability are marked 'no'
**study_focus**	the primary (main) focus (aim) of the study, as stated by the authors in the abstract or introduction	conflict/collisions (e.g., roadkill, vessel strikes, wind turbine collisions, species being harmed to protect persons or property, livestock depredation); conservation (protection, relating to coexistence); disturbance/habitat change (disturbance typically has a negative connotation for species, e.g., deforestation, fragmentation, poaching, ecological traps, urbanization, development, pollution, or technology; habitat or land use change can refer to e.g., intensification of a certain land cover type like

		agriculture); exploratory (e.g., a case study for a new model or framework; a study focusing on understanding driving factors of a species' distribution); food/economics (related to human gain; agricultural productivity; animals for hunting or recreation; harvested species of economic importance; species affecting agriculture; species with beneficial services to humans; disease vectors affecting recreational species); human health/safety (medicinal importance; medicinal plants; disease vectors affecting humans); invasions (human-caused distribution that affects the environment; invasive/introduced range expansions); reintroduction/restoration (including translocation, or range expansion over time)
**study_area_scale**	description of the geographic scale of the study area	global; continental; multinational (studies that cross political borders); national; regional (within-country; multiple states, provinces, or municipalities); local (province, state, town, county, municipality); if multiple scales are used (e.g., in model training versus projection or in comparative studies), the largest scale is noted
**study_area_country**	study area country	if multinational, countries are listed as semi-colon-separated (;); continental and global scale articles are listed at their respective scales only
**time**	general idea on the time frames of focus in the article	past-only or present-only (predicting); past-present, past-present-future, past-future, or present-future (forecasting); present-past (hindcasting)
**hum_time**	general idea on the time frames used for human predictors in the article (i.e., in model training and projection)	past-only or present-only (predicting); past-present, past-present-future, past-future, or present-future (forecasting); present-past (hindcasting)
**time_start**	earliest year of the study (typically determined from dates of occurrence data or inferred from years of the predictors, especially if hindcasting is done—whichever is earliest)	YYYY

**time_end**	latest year of the study (typically determined from dates of occurrence data or from years of the predictors, especially if forecasting is done—whichever is latest)	YYYY
**future_time_start**	considering e.g., climate change scenarios and how human predictors are transferred over time, this is the first year of the future climate interval for articles that do the forecasts; the year may be the exact start year or the average start year, based on how it is reported by the authors	YYYY
**future_time_end**	the latest (last) year of the future climate interval for articles that do forecasts; the year may be the exact end year or the average end year, based on how it is reported by the authors	YYYY
**taxa**	taxonomic group representing the species studied	amphibians; birds; fish; herbaceous plants; invertebrates; mammals; microorganisms; reptiles; trees/shrubs; if multiple taxa, then they are listed as semi-colon-separated (;)
**ttl_species**	total number of species modeled in the article	#
**domain**	environmental domain(s), or, habitat, of the study area	terrestrial, freshwater, or marine
**SDM_algorithm**	the species distribution model (SDM) algorithm used; here, we only focus on correlative SDMs that use species presence/ (pseudo-)absence or presence/ background data as the response variable (i.e., the predictions are probability of presence/occupancy/detection/use, and not count/density/abundance/ richness, etc.)	ANN (artificial neural network); BRT (boosted regression tree); CART; CTA (classification tree analysis); DOMAIN (also known as Gower's distance); ENFA (environmental niche factor analysis); Favorability function; FDA (flexible discriminant analysis); GAM (generalized additive model); GARP (genetic algorithm for rule-set production); GBM (gradient boosting model, including TreeNet); GLM (general/generalized linear model); Hierarchical model (typically customized learning method; e.g. Bayesian inference); Logistic regression; Mahalanobis distance; MARS (multivariate adaptive regression splines); Maxent (maximum entropy);

		MaxLike (maximum likelihood); MDA (mixture discriminant analysis); Occupancy model; Penrose distance (also known as orthogonal distance or perpendicular distance); RF (random forest); RSF (resource selection function); SRE (surface range envelope, also known as BIOCLIM); SVM (support vector machine); ensemble (multiple SDM algorithms combined via e.g., weighted averaging); multiple (multiple SDM algorithms, but not combined as an ensemble)
**SDM_algorithm_ ensembles**	list of SDM algorithms used within an ensemble or multiple-SDM study	see 'SDM_algorithm'; list is semi-colon-separated (;)
**hum_preds**	list of human predictors used in SDM of the study (note that while other human predictors may have been mentioned in the article, when indicated, only those ultimately selected after collinearity tests or model selection are listed here)	unique names; multiple entries, separated by semi-colon (;)
**hum_pred_type**	data types (transformations) of the human predictors	unique names; multiple entries, separated by semi-colon (;); density/count (frequencies or sums); descriptive (categorical, factor, presence/absence); distance (continuous or classified); index (calculated from combination of other predictors); size (area, length); note that human predictors relating to e.g. sampling bias or observer effort probability are excluded
**hum_pred_cat**	human predictor category (type)	12 categories: barriers/access (e.g., fence presence/absence, passable/impassable stream barriers); disturbance (e.g., fragmentation, deforestation, avoidance); energy/raw materials (e.g., wind farms, dams, seismic lines, mines); food/agriculture; human presence (e.g., human footprint, human influence index); infrastructure (e.g., developed areas); management/interventions (e.g., protected area distance); pollution (e.g., night light intensity, count of

		poisoning incidents); recreation/tourism (e.g., trails, scenic locations); socio-economics (e.g., income, education, state names, illegal activities, gross domestic product); transportation (e.g., roads, shipping); ambiguous (predictors that can equally represent environmental predictors as they do human predictors (e.g., land use/land cover, forested/unforested areas)
**hum_amb_pred**	list of ambiguous predictors used in the SDM (if any)	Some predictors can equally represent environmental predictors as much as human predictors due to their format (e.g., land use/land cover or forested/unforested areas). Environmental predictors such as forest cover, forest edge, or fire frequency are included in the list of human predictors when the authors explicitly state their relationship to anthropogenic effects on species distributions (e.g., deforestation, fragmentation, or fires due to land use intensification); this data field is a subset of the hum_preds data field
**num_env_preds**	total number of environmental predictors that were used in the model	#; environmental predictors are any non-human predictors in the SDM; these are typically climatic, topographic, edaphic, or refer to vegetation cover; predictors related to sampling bias or effort are excluded
**num_hum_preds**	total number of human predictors that were used in the SDM	#; this number excludes any predictors that are not within SDM training (e.g., from post-SDM analyses); note that ambiguous predictors are included here as a human predictor; human predictors relating to e.g., sampling bias or observer effort probability are excluded
**num_amb_pred**	number of ambiguous predictors	#
**worldclim**	indicates whether the article uses a common set of bioclimatic predictors/data sources in their study	yes, no, unk (unknown); an indicator of uniformity (common structures, consensus) in SDM procedures across studies
**qual_eval**	qualitative, holistic evaluation made by authors on how models using human predictors	better, worse, no_difference, depends (e.g., differences depending on scale, resolution or modeled species),

	performed compared to models using only environmental predictors	not_stated; this holistic assessment was usually found in the discussion, and is only based on authors' statements; author evaluations could be based on e.g., metrics (accuracy, predictor importance, significance) and/or differences in probability of presence predicted from the models.
**reject_code**	reason for rejecting a full article from the systematic review	#; numeric code assigned as one of the following: (1) not a traditional, correlative SDM for modeling species distributions (e.g. species abundance or density models or deductive, expert-opinion models are rejected; see Table S3 for list of typical SDM algorithms that are accepted); (2) no human predictors were used in SDM model training (i.e., no human predictors in the paper, or human predictors are used as masks, detection probability estimates, or in a post-analysis of an SDM); (3) not a research article of modeling species distributions (e.g., a book chapter, literature review, or a model of disease, fire, cover, or virtual species), or the authors use SDMs from another source

Supplementary Table 3. Qualitative evaluation summary of human predictor performance

Supplementary Table 3. Summary of qualitative, holistic evaluations made by authors on how SDMs using human and environmental predictors performed compared to SDMs using only environmental predictors. Out of the 1,429 accepted articles, a total of 127 of them ran SDMs with and without human predictors, of which 97 provided a qualitative evaluation in either the results or discussion section of the articles. Further information can be found in the corresponding articles listed under 'article ID', which match their unique identification numbers (UID) searchable in Supplementary Table 5.

human predictor performance	study focus	article ID	no. articles
**better**	reintroduction; invasions; conservation; disturbance; exploratory; human food security; protection; human health; human-wildlife conflict; habitat change; human economics	755; 1658; 2935; 3040; 3112; 4550; 4563; 4581; 4762; 4944; 5174; 5334; 5394; 5554; 5829; 5903; 5999; 6360; 7016; 7240; 7605; 7874; 7990; 8300; 8654; 8712; 8750; 8761; 9108; 9398; 9521; 9539; 9740; 9785; 10187; 10360; 10545; 10640; 10747; 10813; 11413; 11640; 11814	43
**no difference**	invasions; exploratory; conservation; human food security; habitat change	632; 3134; 4355; 4595; 4918; 5284; 5427; 5594; 5643; 6343; 6522; 6622; 6856; 7537; 8287; 9501; 10984; 11008	18
**worse**	exploratory; habitat change; human gain; invasions; illegal activity; conservation	498; 1573; 3302; 5627; 6787; 6978; 8486; 10334; 10989; 11625	10
**depends**	exploratory; invasions; habitat change; restoration; disturbance; conservation; human health; reintroduction	408; 1048; 2367; 2484; 3854; 5192; 5294; 5411; 5863; 6422; 7201; 8404; 8482; 8516; 8663; 8840; 9326; 9678; 9782; 9957; 10431; 11427; 11474; 11618; 11958; 12246	26
**not stated**	conservation; invasions; reintroduction; human food security; human disturbance; exploratory; protection; disturbance; human economics; human health	106; 188; 1576; 3130; 3238; 3601; 4159; 4396; 4818; 6211; 6300; 6347; 6834; 7468; 7618; 7944; 8548; 8736; 8858; 9182; 9235; 9673; 9938; 9939; 10047; 10829; 10959; 11230; 11404; 11425	30
**NA (not assessed)**	NA	NA	1302

Supplementary Table 4. List of human predictors used in SDM studies

Supplementary Table 4. List of the 2,307 unique human predictors used in the 1,429 full articles identified in the systematic review and synthesis. The predictors are sorted by category, data type, and predictor name. Details on the 12 categories, 5 data types, and range in taxa and study focus are described in Table S2. Predictor descriptions can be found in the corresponding articles that used them, which are listed in Supplementary Tables 5-6. The SDG codes correspond to the following: SDG-01: No Poverty; SDG-02: Zero Hunger; SDG-03: Good Health and Well-being; SDG-04: Quality Education; SDG-05: Gender Equality; SDG-06: Clean Water and Sanitation; SDG-07: Affordable and Clean Energy; SDG-08: Decent Work and Economic Growth; SDG-09: Industry, Innovation and Infrastructure; SDG-10: Reduced Inequality; SDG-11: Sustainable Cities and Communities; SDG-12: Responsible Consumption and Production; SDG-13: Climate Action; SDG-14: Life Below Water; SDG-15: Life on Land; SDG-16: Peace and Justice Strong Institutions; SDG-17: Partnerships for the Goals. First and last year refer to the first and last (most recent) year that a human predictor was used in one of the full articles.

category	data type	predictor	time	SDG code	taxa	study focus	no.	first year	last year
ambiguous	density/count	abandoned areas percent	past; present; future		birds	disturbance/habitat change	1	2010	2010
ambiguous	density/count	herbaceous areas density 50m radius	present		birds	invasions	1	2018	2018
ambiguous	density/count	intactness percent	present		fish	conservation	1	2020	2020
ambiguous	density/count	land use low percent	past; present	15	fish	conservation	1	2021	2021
ambiguous	density/count	non-forest secondary land percent	present; future	15	mammals	disturbance/habitat change	1	2021	2021
ambiguous	density/count	open areas percent historic yr1830	present		herbaceous plants	invasions	1	2019	2019
ambiguous	density/count	openness percent downstream	present		fish	conservation	1	2019	2019
ambiguous	density/count	openness percent upstream	present		fish	conservation	1	2019	2019
ambiguous	density/count	pastures and open areas percent	present		mammals	invasions	1	2016	2016
ambiguous	density/count	rangeland and forested areas percent	present		mammals	conservation	1	2018	2018

ambiguous	density/count	urban and transports land cover percent	present	06; 11; 15	invertebrates	exploratory	1	2020	2020
ambiguous	density/count	urban crops or urban herbaceous areas percent	present	02; 06; 11	amphibians	exploratory	1	2015	2015
ambiguous	density/count	urban herbaceous areas percent	present	06; 11	amphibians	exploratory	1	2015	2015
ambiguous	descriptive	abandoned areas with vegetation	present		birds	exploratory	1	2012	2012
ambiguous	descriptive	burn natural or unnatural	present		herbaceous plants	invasions	1	2013	2013
ambiguous	descriptive	forest non-forest	past; present; future	15	mammals; herbaceous plants; invertebrates; birds	conservation; disturbance/habitat change; conflict/collisions; human health/safety; exploratory	5	2007	2021
ambiguous	descriptive	forest stands 15-30yrs old	present	15	mammals	reintroduction/restoration	1	2015	2015
ambiguous	descriptive	historic vegetation	present		birds	reintroduction/restoration	1	2020	2020
ambiguous	descriptive	land use and vegetation type	present	15	invertebrates	human health/safety	1	2020	2020
ambiguous	descriptive	land use historic	present	15	amphibians; birds; reptiles	disturbance/habitat change	1	2021	2021
ambiguous	descriptive	land use historic yr1888	present	15	herbaceous plants	invasions	1	2011	2011
ambiguous	descriptive	land use historic yr1951	present	15	trees/shrubs	exploratory	1	2013	2013
ambiguous	descriptive	land use historic yr1971	present	15	trees/shrubs	exploratory	1	2013	2013
ambiguous	descriptive	land use historic yr1990	present	15	herbaceous plants	invasions	1	2012	2012
ambiguous	descriptive	land use historic yr2000	present	15	herbaceous plants	invasions	1	2012	2012
ambiguous	descriptive	land use other	past; present	15	invertebrates	exploratory	1	2014	2014
ambiguous	descriptive	land use other subbasin	present	15	fish	conservation	1	2018	2018

ambiguous	descriptive	land use other upstream	present	15	fish	conservation	1	2018	2018
ambiguous	descriptive	land use permanent crop subbasin	present	15	fish	conservation	1	2018	2018
ambiguous	descriptive	land use simplified	present	15	mammals	conservation	1	2017	2017
ambiguous	descriptive	land use/land cover	past; present; future	15	mammals; herbaceous plants; birds; reptiles; invertebrates; amphibians; trees/shrubs; microorganisms; fish	conflict/collisions; disturbance/habitat change; exploratory; invasions; conservation; reintroduction/restoration; human health/safety; food/economics	397	2000	2021
ambiguous	descriptive	land use/land cover 1km radius	present	15	herbaceous plants	invasions	1	2018	2018
ambiguous	descriptive	land use/land cover 2080	future	15	herbaceous plants	disturbance/habitat change	1	2014	2014
ambiguous	descriptive	land use/land cover 4km radius	present	15	reptiles; herbaceous plants	invasions	2	2018	2018
ambiguous	descriptive	land use/land cover adjacent area area size	present	15	mammals	disturbance/habitat change	1	2018	2018
ambiguous	descriptive	land use/land cover adjacent percent	present	15	mammals	disturbance/habitat change	1	2018	2018
ambiguous	descriptive	land use/land cover aggregation index	present	15	mammals	conservation	1	2011	2011
ambiguous	descriptive	land use/land cover change class	present	01; 15	invertebrates	conservation	1	2010	2010
ambiguous	descriptive	land use/land cover change percent	present	15	microorganisms	human health/safety	1	2020	2020
ambiguous	descriptive	land use/land cover change rate	past; present; future	15	mammals	disturbance/habitat change	1	2020	2020

ambiguous	descriptive	land use/land cover class	present	01; 15	invertebrates; mammals	human health/safety; conservation	2	2019	2019
ambiguous	descriptive	land use/land cover class sum	present; future	01; 15	birds	exploratory	1	2009	2009
ambiguous	descriptive	land use/land cover cohesion	present	15	mammals	exploratory	1	2015	2015
ambiguous	descriptive	land use/land cover contagion	present	15	mammals; birds; fish; invertebrates	reintroduction/restoration; disturbance/habitat change; exploratory	4	2009	2021
ambiguous	descriptive	land use/land cover count	present	15	invertebrates	disturbance/habitat change	2	2017	2019
ambiguous	descriptive	land use/land cover density	present	15	mammals	conservation	1	2021	2021
ambiguous	descriptive	land use/land cover dominant	present	15	herbaceous plants	invasions	1	2021	2021
ambiguous	descriptive	land use/land cover dominant 2000m radius	present	15	fish	conservation	1	2019	2019
ambiguous	descriptive	land use/land cover dominant 4.5km radius	present	15	mammals	conflict/collisions	1	2015	2015
ambiguous	descriptive	land use/land cover dominant 500m radius	present	15	herbaceous plants	invasions	1	2017	2017
ambiguous	descriptive	land use/land cover edge density	present	15	mammals	conservation	1	2021	2021
ambiguous	descriptive	land use/land cover evenness	present	15	amphibians; invertebrates; mammals; reptiles	conflict/collisions	1	2021	2021
ambiguous	descriptive	land use/land cover fragmentation 1km radius	present	15	birds	disturbance/habitat change	1	2013	2013
ambiguous	descriptive	land use/land cover generic	present	15	birds	exploratory	1	2016	2016
ambiguous	descriptive	land use/land cover historic 1870	present	15	herbaceous plants	exploratory	1	2016	2016
ambiguous	descriptive	land use/land cover historic yr1960	present	15	herbaceous plants	exploratory	1	2016	2016

ambiguous	descriptive	land use/land cover index 100m radius	present	15	fish	reintroduction/restoration	1	2021	2021
ambiguous	descriptive	land use/land cover index 1km radius	present	15	fish	reintroduction/restoration	1	2021	2021
ambiguous	descriptive	land use/land cover interspersion	present	15	invertebrates; birds	human health/safety; conservation	2	2016	2021
ambiguous	descriptive	land use/land cover matrix index	present	15	reptiles	exploratory	1	2011	2011
ambiguous	descriptive	land use/land cover mean	present	15	mammals	conservation	1	2021	2021
ambiguous	descriptive	land use/land cover open	present	15	birds	conservation	1	2021	2021
ambiguous	descriptive	land use/land cover other	present; future	15	invertebrates	disturbance/habitat change	1	2013	2013
ambiguous	descriptive	land use/land cover other percent	present	15	birds; herbaceous plants; invertebrates	exploratory	1	2011	2011
ambiguous	descriptive	land use/land cover patch count	past; present	15	mammals	exploratory; conservation	2	2004	2021
ambiguous	descriptive	land use/land cover patch density 100ha radius	present	15	invertebrates	exploratory	1	2009	2009
ambiguous	descriptive	land use/land cover patch mean size	present	15	mammals	conservation	1	2004	2004
ambiguous	descriptive	land use/land cover patch richness	present	15	birds	conservation	1	2019	2019
ambiguous	descriptive	land use/land cover patch size	present	15	herbaceous plants	exploratory	1	2018	2018
ambiguous	descriptive	land use/land cover percent	present	15	birds; mammals	exploratory	2	2015	2021
ambiguous	descriptive	land use/land cover percent 2.25ha radius	present	15	birds	exploratory	1	2010	2010
ambiguous	descriptive	land use/land cover percent 20.25ha radius	present	15	birds	exploratory	1	2010	2010
ambiguous	descriptive	land use/land cover richness	present	15	invertebrates; birds; mammals	human health/safety; conflict/collisions; exploratory	3	2012	2021

ambiguous	descriptive	land use/land cover shape index	present	15	birds	disturbance/habitat change	1	2015	2015
ambiguous	descriptive	land use/land cover specific	present	15	birds	exploratory	1	2016	2016
ambiguous	descriptive	pond isolation class	present	1	amphibians	disturbance/habitat change	1	2016	2016
ambiguous	descriptive	remnant or nonremnant vegetation	present		mammals	conservation	1	2020	2020
ambiguous	descriptive	secondary land	present; future		mammals	exploratory	1	2021	2021
ambiguous	descriptive	secondary land forested	present; future	15	amphibians; birds; mammals; reptiles	invasions	1	2021	2021
ambiguous	descriptive	secondary land non-forested	present; future	15	amphibians; birds; mammals; reptiles	invasions	1	2021	2021
ambiguous	descriptive	unexploited areas	present		herbaceous plants	exploratory	1	2009	2009
ambiguous	descriptive	unproductive and non-forested areas	present		mammals	exploratory	1	2021	2021
ambiguous	distance	artificial or natural water body distance	present	6	birds; invertebrates; mammals	conservation	1	2015	2015
ambiguous	distance	degraded forest distance	present	15	mammals	food/economics	1	2018	2018
ambiguous	distance	forest distance	present; future	15	mammals	exploratory	2	2011	2014
ambiguous	distance	forests natural and commercial distance	present	15	birds	conflict/collisions	1	2020	2020
ambiguous	distance	land use/land cover change distance	present	15	herbaceous plants	invasions	1	2014	2014
ambiguous	distance	non-forest distance	present	15	birds	exploratory	1	2007	2007
ambiguous	distance	remnant native habitat distance	present		amphibians	disturbance/habitat change	1	2016	2016

ambiguous	distance	roads to forest distance	present	15	herbaceous plants	invasions	1	2014	2014
ambiguous	index	land condition index	present		birds	exploratory	1	2021	2021
ambiguous	index	land use change percent	present	15	fish; birds	conservation	2	2017	2019
ambiguous	index	land use change percent forest to open percent	present	15	herbaceous plants	invasions	1	2014	2014
ambiguous	index	land use change percent open to forest percent	present	15	herbaceous plants	invasions	1	2014	2014
ambiguous	index	land use change percent percent	present	15	herbaceous plants	invasions	1	2014	2014
ambiguous	index	land use diversity	present; future	15	birds; mammals	conservation; exploratory; reintroduction/restoration; food/economics	6	2006	2016
ambiguous	index	land use intensity	present	15	birds; herbaceous plants; invertebrates	disturbance/habitat change; conservation	2	2010	2016
ambiguous	index	land use low-intensity percent	present	15	invertebrates	conservation	1	2021	2021
ambiguous	index	land use medium or high-intensity percent	present	15	invertebrates	conservation	1	2021	2021
ambiguous	index	land use medium or high-intensity percent 100m radius	present	15	invertebrates	conservation	1	2021	2021
ambiguous	index	land use richness	present	15	birds	exploratory	1	2014	2014
ambiguous	index	land use/land cover diversity	present; future	15	birds; herbaceous plants; amphibians; invertebrates; mammals; reptiles	exploratory; invasions; conflict/collisions; reintroduction/restoration; human health/safety; conservation; disturbance/habitat change	25	2003	2021

ambiguous	index	land use/land cover diversity 1km radius	present	15	birds	disturbance/habitat change; reintroduction/restoration	2	2009	2013
ambiguous	index	land use/land cover diversity 2.5km radius	present	15	birds	reintroduction/restoration	1	2009	2009
ambiguous	index	land use/land cover diversity 5km radius	present	15	birds	reintroduction/restoration	1	2009	2009
ambiguous	index	land use/land cover edge diversity	present	15	birds	conservation	1	2016	2016
ambiguous	index	landscape condition index	present		herbaceous plants; birds	invasions; conservation	2	2016	2020
ambiguous	index	landscape diversity	present		birds	exploratory	1	2011	2011
ambiguous	index	landscape integrity	present		amphibians; birds; mammals; reptiles	exploratory	1	2017	2017
ambiguous	index	pastures and open areas clumpiness	present		mammals	invasions	1	2016	2016
ambiguous	index	wildness index	present		birds	conservation	1	2021	2021
ambiguous	size	land use size	present	15	invertebrates	conservation	1	2020	2020
ambiguous	size	remnant vegetation area size	present		birds	reintroduction/restoration	1	2020	2020
ambiguous	time	land use previous year	present	15	birds	conservation	1	2010	2010
ambiguous	time	time since land abandonment in years	present		herbaceous plants; invertebrates	conservation	1	2011	2011
barriers/access	density/count	fencing areas frequency	present		mammals	invasions	1	2013	2013
barriers/access	density/count	hedgerows and woodlands percent	present		birds	exploratory	1	2021	2021
barriers/access	density/count	hedgerows density	present		mammals; invertebrates	conflict/collisions; exploratory	2	2017	2020
barriers/access	density/count	hedgerows percent	present		invertebrates; birds	human health/safety; exploratory	4	2015	2020

barriers/access	density/count	hedgerows percent 1km radius	present		birds	disturbance/habitat change	1	2013	2013
barriers/access	density/count	water flow obstacles upstream density	past; present	6	mammals	exploratory	1	2016	2016
barriers/access	descriptive	country boundary	present		herbaceous plants	exploratory	1	2019	2019
barriers/access	descriptive	fence	present		mammals	disturbance/habitat change	1	2018	2018
barriers/access	descriptive	fence presence	present		mammals	conservation	1	2012	2012
barriers/access	descriptive	hedgerows presence	present		birds	exploratory; disturbance/habitat change	2	2013	2015
barriers/access	descriptive	human access density class	present	1	mammals	exploratory	1	2002	2002
barriers/access	descriptive	impassable stream barrier	future		fish	conservation	1	2018	2018
barriers/access	descriptive	passable stream barrier	present; future		fish	conservation	1	2018	2018
barriers/access	descriptive	walls length	present		herbaceous plants	invasions	1	2013	2013
barriers/access	descriptive	walls south-facing	present		reptiles	invasions; exploratory	2	2016	2018
barriers/access	distance	accessible lake from natural-unnatural barrier distance	present		fish	exploratory	1	2021	2021
barriers/access	distance	barrier distance	present		mammals	reintroduction/restoration	1	2018	2018
barriers/access	distance	fence distance	present		mammals	food/economics; reintroduction/restoration	2	2012	2017
barriers/access	distance	fence distance 1km max	present		mammals	reintroduction/restoration	1	2012	2012
barriers/access	distance	hedgerows distance percent	present		mammals	exploratory	1	2020	2020
barriers/access	distance	park boundary distance	present; future		mammals	disturbance/habitat change	1	2020	2020
barriers/access	distance	park fence distance	present		mammals	conservation	1	2014	2014
barriers/access	distance	unfenced park boundary distance	present		mammals	conservation	1	2014	2014
barriers/access	index	hedgerows density index	present		mammals	human health/safety	1	2014	2014

barriers/access	index	human accessibility	present	invertebrates; fish; herbaceous plants; microorganisms; amphibians; birds; mammals; reptiles; trees/shrubs	invasions	2	2017	2020
barriers/access	size	hedgerows continuous length	present	birds	exploratory	1	2012	2012
barriers/access	size	hedgerows interrupted length	present	birds	exploratory	1	2012	2012
barriers/access	size	hedgerows length	past; present; future	birds	disturbance/habitat change; exploratory	2	2010	2015
barriers/access	size	wall length	present; future	birds	conservation	1	2019	2019
disturbance	density/count	artificial areas percent	present	mammals; birds	exploratory; invasions	4	2013	2021
disturbance	density/count	artificial surfaces percent	present; future	amphibians; trees/shrubs; mammals; invertebrates; herbaceous plants; birds; fish; microorganisms; reptiles	conservation; invasions; human health/safety; exploratory; disturbance/habitat change	8	2006	2021
disturbance	density/count	bare ground percent	present	birds	conservation	1	2010	2010
disturbance	density/count	cleared or disturbed land percent	present	birds	disturbance/habitat change	1	2011	2011
disturbance	density/count	cutovers and burns percent	present	reptiles	exploratory	1	2012	2012

disturbance	density/count	degraded habitat patch percent 8km radius	present		mammals	exploratory	1	2021	2021
disturbance	density/count	disturbed forest density	past; present	15	mammals	disturbance/habitat change	1	2015	2015
disturbance	density/count	exotic species density 1000m radius	present		birds	invasions	1	2018	2018
disturbance	density/count	exotic species density 500m radius	present		birds	invasions	1	2018	2018
disturbance	density/count	exotic species density 50m radius	present		birds	invasions	1	2018	2018
disturbance	density/count	forest burns recent percent	present	15	birds	conservation	1	2014	2014
disturbance	density/count	forest cut burn <10yrs percent	present	15	birds	conservation	1	2014	2014
disturbance	density/count	forest cut burn 10-20yrs percent	present	15	birds	conservation	1	2014	2014
disturbance	density/count	forest cut recent percent	present	15	birds	conservation	1	2014	2014
disturbance	density/count	fragmentation density	present		fish	disturbance/habitat change	1	2013	2013
disturbance	density/count	fragmentation percent	past; present		birds	conservation	1	2017	2017
disturbance	density/count	gross canopy loss 2000-2016	present		mammals	human health/safety	1	2018	2018
disturbance	density/count	logging cut-block areas percent	present		mammals	disturbance/habitat change	1	2019	2019
disturbance	density/count	logging cut-block density 12k radius	present		mammals	exploratory	1	2012	2012
disturbance	density/count	logging cut-block density 5k radius	present		mammals	exploratory	1	2012	2012
disturbance	density/count	logging cut-block density 70m radius	present		mammals	exploratory	1	2012	2012
disturbance	density/count	logging percent	present		mammals	exploratory	1	2014	2014
disturbance	density/count	logging roads density	present		mammals	exploratory	2	2014	2016
disturbance	density/count	natural and semi-natural areas percent	present		herbaceous plants	invasions	1	2016	2016

disturbance	density/count	road-stream crossings density local watershed	present	fish	conservation	1	2020	2020
disturbance	density/count	road-stream density crossings upstream watershed	present	fish	conservation	1	2020	2020
disturbance	density/count	roads and stream crossings and slope catchment density	present	invertebrates	conservation	1	2021	2021
disturbance	density/count	roads and stream crossings and slope river density	present	invertebrates	conservation	1	2021	2021
disturbance	density/count	roads and stream crossings catchment density	present	invertebrates	conservation	1	2021	2021
disturbance	density/count	semi-natural areas percent	past; present	mammals	exploratory; conservation	2	2013	2016
disturbance	density/count	semi-natural grassland percent	present	invertebrates	food/economics	1	2013	2013
disturbance	density/count	stream crossings count local watershed	present	fish	conservation	1	2015	2015
disturbance	density/count	stream crossings count upstream watershed	present	fish	conservation	1	2015	2015
disturbance	descriptive	agricultural semi-natural areas	present	invertebrates	disturbance/habitat change	1	2018	2018
disturbance	descriptive	artificial areas	present	mammals; herbaceous plants	reintroduction/restoration; invasions	2	2017	2020
disturbance	descriptive	artificial surfaces	present; future	mammals; invertebrates	conflict/collisions; disturbance/habitat change; reintroduction/restoration	3	2013	2017
disturbance	descriptive	artificial surfaces area size	present	birds; herbaceous plants	exploratory; disturbance/habitat change	2	2013	2013
disturbance	descriptive	bare land	present; future	birds	exploratory	1	2009	2009

disturbance	descriptive	burnt stubble presence	present		birds	disturbance/habitat change	1	2011	2011
disturbance	descriptive	deforestation curvature profile	present	15	birds; herbaceous plants; invertebrates	disturbance/habitat change	1	2016	2016
disturbance	descriptive	degraded areas	present		herbaceous plants	invasions	1	2018	2018
disturbance	descriptive	destroyed and abandoned areas	present		mammals	conservation	1	2018	2018
disturbance	descriptive	disturbed forest	present	15	invertebrates; mammals	human health/safety	1	2016	2016
disturbance	descriptive	forest fragmentation class	present	01; 15	mammals	reintroduction/restoration	1	2020	2020
disturbance	descriptive	forest non-forest deforested class	present; future	01; 15	mammals	disturbance/habitat change	1	2020	2020
disturbance	descriptive	harvested cut-block <30yrs	present		birds	exploratory	1	2008	2008
disturbance	descriptive	hunting pressure class	present	1	mammals	disturbance/habitat change	1	2015	2015
disturbance	descriptive	logging cut-block areas	present		mammals; herbaceous plants; trees/shrubs	invasions; disturbance/habitat change; reintroduction/restoration; exploratory	5	2012	2020
disturbance	descriptive	logging cuts <=5yrs old	present		mammals	reintroduction/restoration	1	2015	2015
disturbance	descriptive	logging industrial	present		birds	disturbance/habitat change	1	2000	2000
disturbance	descriptive	logging regenerating cuts 5-10yrs old	present		mammals	reintroduction/restoration	1	2015	2015
disturbance	descriptive	meadow exploited	present		herbaceous plants	exploratory	1	2009	2009
disturbance	descriptive	recent burn regenerating broad-leaved forest	present	15	birds	disturbance/habitat change	1	2013	2013

disturbance	descriptive	recent burn regenerating conifer forest	present	15	birds	disturbance/habitat change	1	2013	2013
disturbance	descriptive	riverbank alteration class	present	1	mammals	conservation	1	2006	2006
disturbance	descriptive	semi-natural areas	present		mammals	reintroduction/restoration	1	2017	2017
disturbance	descriptive	undisturbed areas percent	present		microorganisms	exploratory	1	2018	2018
disturbance	descriptive	undisturbed desert	present		microorganisms	disturbance/habitat change	1	2018	2018
disturbance	descriptive	undisturbed forest	present	15	microorganisms	disturbance/habitat change	1	2018	2018
disturbance	descriptive	undisturbed other	present		microorganisms	disturbance/habitat change	1	2018	2018
disturbance	descriptive	undisturbed steppe	present		microorganisms	disturbance/habitat change	1	2018	2018
disturbance	descriptive	undisturbed tropical	present		microorganisms	disturbance/habitat change	1	2018	2018
disturbance	descriptive	undisturbed tundra	present		microorganisms	disturbance/habitat change	1	2018	2018
disturbance	descriptive	water risk status	present	6	herbaceous plants	exploratory	1	2020	2020
disturbance	distance	artificial areas distance	present; future		mammals; birds	invasions; conservation	2	2016	2019
disturbance	distance	artificial surfaces distance	present		birds	exploratory	1	2018	2018
disturbance	distance	deforestation distance	present	15	mammals	conflict/collisions	1	2015	2015
disturbance	distance	deforestation historic distance	past; present; future	15	mammals	disturbance/habitat change	1	2020	2020
disturbance	distance	industrial chronic noise source distance	present		amphibians	conservation	1	2019	2019
disturbance	distance	logging distance	present		mammals	disturbance/habitat change	1	2019	2019
disturbance	distance	logging roads distance	present		mammals	conservation; exploratory	2	2016	2019
disturbance	distance	logging roads distance mean	present		mammals	conservation	1	2006	2006
disturbance	distance	logging sawmills distance	present		invertebrates	invasions	1	2019	2019

disturbance	distance	vegetation removal distance	present		invertebrates	human health/safety	1	2018	2018
disturbance	index	agricultural impact index	present		birds	disturbance/habitat change	1	2011	2011
disturbance	index	anthropogenic fragmentation	present		invertebrates	disturbance/habitat change	1	2013	2013
disturbance	index	fire frequency	present		herbaceous plants; mammals	invasions; exploratory	2	2014	2018
disturbance	index	fire frequency index	present		mammals	conservation	1	2006	2006
disturbance	index	fishing pressure	present	14	invertebrates	conservation	1	2017	2017
disturbance	index	forest fragmentation	present	15	mammals	conservation	2	2010	2015
disturbance	index	forest fragmentation index	present	15	birds	disturbance/habitat change	1	2015	2015
disturbance	index	forest loss 10yr mean	present	15	mammals	exploratory	1	2017	2017
disturbance	index	fragmentation index	present		birds	exploratory	1	2018	2018
disturbance	index	grazing pressure index	present		birds	disturbance/habitat change	1	2020	2020
disturbance	index	habitat fragmentation	present	15	birds	reintroduction/restoration	1	2011	2011
disturbance	index	habitat threat index local watershed	present		fish	conservation	1	2020	2020
disturbance	index	habitat threat index upstream watershed	present		fish	conservation	1	2020	2020
disturbance	index	human-induced extirpation risk	past; present; future		mammals	conservation	1	2021	2021
disturbance	index	human impact index	present		reptiles	conservation	1	2012	2012
disturbance	index	human land transformation	present		herbaceous plants	exploratory	1	2006	2006
disturbance	index	human modification index	present		invertebrates; mammals; birds	invasions; exploratory; conservation	3	2020	2021
disturbance	index	human perturbation index	present		trees/shrubs	conservation	1	2013	2013
disturbance	index	human population pressure	present	11	microorganisms	conservation	1	2020	2020
disturbance	index	hunting pressure percent	present		mammals	conflict/collisions	1	2016	2016

disturbance	index	land transformation	present		mammals	reintroduction/restoration	1	2008	2008
disturbance	index	landscape avoidance index	present		mammals	invasions; reintroduction/restoration	2	2006	2009
disturbance	index	landscape fragmentation	present		mammals	conservation	1	2020	2020
disturbance	index	marine human impact index	present	14	microorganisms	conservation	1	2020	2020
disturbance	index	marine human impacts	present	14	fish; herbaceous plants; invertebrates; microorganisms; mammals; reptiles; trees/shrubs	invasions	1	2015	2015
disturbance	index	noise level	present		birds	exploratory	1	2021	2021
disturbance	index	poaching index	present		mammals	conflict/collisions	1	2013	2013
disturbance	index	roads traffic noise	present		birds	disturbance/habitat change	1	2020	2020
disturbance	index	shipping noise tonal detection frequency	present		mammals	disturbance/habitat change	1	2021	2021
disturbance	index	sound pressure level	present		mammals	disturbance/habitat change	1	2021	2021
disturbance	index	toxic pressure msPAF	present		fish	disturbance/habitat change	1	2017	2017
disturbance	size	fragmentation size	present		mammals	exploratory	1	2016	2016
disturbance	size	habitat fragmentation suitable patch size	present	15	invertebrates	disturbance/habitat change	1	2017	2017
disturbance	size	logging cut-block areas size	present; future		birds	disturbance/habitat change	1	2021	2021
disturbance	size	modified habitat area size	present		birds	conservation	1	2016	2016
disturbance	size	semi-natural areas area	present		birds	conservation	1	2007	2007
disturbance	size	semi-natural areas weighted area	present		birds	conservation	1	2007	2007
disturbance	time	logging duration	present		birds	exploratory	1	2020	2020

disturbance	time	logging end date	present		birds	exploratory	1	2020	2020
disturbance	time	logging years since cut	present; future		birds	disturbance/habitat change	1	2021	2021
disturbance	time	years since prescribed fire	present		mammals	reintroduction/restoration	1	2019	2019
energy/ raw materials	density/count	agricultural areas using electric energy count	present	7	herbaceous plants	food/economics	1	2018	2018
energy/ raw materials	density/count	bioenergy crops percent	present; future	02; 07	birds	food/economics	1	2014	2014
energy/ raw materials	density/count	clear cut areas harvest <30yrs ago density	present		mammals	disturbance/habitat change	1	2015	2015
energy/ raw materials	density/count	dams count	present		fish	reintroduction/restoration; exploratory	2	2017	2021
energy/ raw materials	density/count	dams density	present		fish; invertebrates	conservation; food/economics	2	2014	2019
energy/ raw materials	density/count	dams density downstream	present		fish; invertebrates	reintroduction/restoration; conservation	3	2018	2021
energy/ raw materials	density/count	dams density local watershed	present		fish	conservation	1	2020	2020
energy/ raw materials	density/count	dams density open downstream	present		fish; invertebrates	conservation	1	2018	2018
energy/ raw materials	density/count	dams density open upstream	present		fish; invertebrates	conservation	1	2018	2018
energy/ raw materials	density/count	dams density upstream	present		fish; invertebrates	reintroduction/restoration; conservation	3	2018	2021
energy/ raw materials	density/count	dams density upstream watershed	present		fish	conservation	1	2020	2020
energy/ raw materials	density/count	dams downstream mainstem density	present		fish	conservation	1	2019	2019
energy/ raw materials	density/count	dams local count	present		fish	invasions	1	2015	2015
energy/ raw materials	density/count	dams mainstem density	present		fish	conservation	1	2019	2019
energy/ raw materials	density/count	dams stream gradient mean	present; future		fish	disturbance/habitat change	1	2020	2020

energy/ raw materials	density/count	dams upstream mainstem density	present		fish	conservation	1	2019	2019
energy/ raw materials	density/count	dams upstream network density	present		fish	conservation	1	2019	2019
energy/ raw materials	density/count	dams upstream total	present		fish	invasions	1	2015	2015
energy/ raw materials	density/count	energy transmission lines density	past; present	7	birds	disturbance/habitat change	1	2017	2017
energy/ raw materials	density/count	mineral extraction sites percent	present		birds	reintroduction/restoration	1	2012	2012
energy/ raw materials	density/count	mines density	present		mammals; invertebrates; fish	conservation; food/economics	5	2013	2021
energy/ raw materials	density/count	mines density catchment	present		fish	reintroduction/restoration	1	2021	2021
energy/ raw materials	density/count	mines percent	present		amphibians	conservation	1	2015	2015
energy/ raw materials	density/count	mines quarries bedrock outcrops percent	present		reptiles	exploratory	1	2012	2012
energy/ raw materials	density/count	mining activities percent	present		reptiles	conflict/collisions	1	2021	2021
energy/ raw materials	density/count	mining mountaintop with valley fills density	present		fish	disturbance/habitat change	1	2013	2013
energy/ raw materials	density/count	mining mountaintop with valley fills percent	present		fish	disturbance/habitat change	1	2013	2013
energy/ raw materials	density/count	natural gas well count 0.04km radius	present		birds	conservation	1	2008	2008
energy/ raw materials	density/count	natural gas well count 0.65km radius	present		birds	conservation	1	2008	2008
energy/ raw materials	density/count	natural gas well count 4km radius	present		birds	conservation	1	2008	2008
energy/ raw materials	density/count	oil gas pipeline density	present		birds	conservation	1	2013	2013

energy/ raw materials	density/count	oil gas roads crossings density	present		fish	disturbance/habitat change	1	2018	2018
energy/ raw materials	density/count	oil gas well density	present		birds	conservation	1	2016	2016
energy/ raw materials	density/count	oil gas wells count	present		birds	food/economics	1	2011	2011
energy/ raw materials	density/count	oil well pads percent	present		mammals	exploratory	1	2014	2014
energy/ raw materials	density/count	powerlines density	present		birds	conservation	1	2013	2013
energy/ raw materials	density/count	powerlines percent	present		birds	reintroduction/restoration	1	2014	2014
energy/ raw materials	density/count	production forest eucalyptus percent	present	15	trees/shrubs	invasions	1	2021	2021
energy/ raw materials	density/count	production forest other percent	present	15	trees/shrubs	invasions	1	2021	2021
energy/ raw materials	density/count	production forest pine percent	present	15	trees/shrubs	invasions	1	2021	2021
energy/ raw materials	density/count	reservoir storage upstream volume in catchment	present		fish	conservation	1	2019	2019
energy/ raw materials	density/count	reservoir storage upstream volume in stream network	present		fish	conservation	1	2019	2019
energy/ raw materials	density/count	reservoir upstream annual discharge percent	present		fish	conservation	1	2019	2019
energy/ raw materials	density/count	reservoirs percent	present		amphibians	conservation	1	2015	2015
energy/ raw materials	density/count	river hydraulic works percent	present		fish	reintroduction/restoration	1	2015	2015
energy/ raw materials	density/count	salt mines percent	present		amphibians	conservation	1	2015	2015
energy/ raw materials	density/count	transmission lines percent	present		birds	disturbance/habitat change	1	2021	2021
energy/ raw materials	density/count	water withdrawl gallons per year catchment	present	6	fish	reintroduction/restoration	1	2021	2021

energy/ raw materials	density/count	well density	present		birds	exploratory; conservation	2	2016	2021
energy/ raw materials	density/count	wind power watts	present	7	birds	conservation	1	2020	2020
energy/ raw materials	density/count	wind turbines count	present	7	birds	exploratory	1	2015	2015
energy/ raw materials	density/count	wind turbines density 20km radius	present	7	birds	conservation	1	2017	2017
energy/ raw materials	descriptive	clear cut areas	present		mammals; reptiles	exploratory; conservation; disturbance/habitat change	4	2006	2020
energy/ raw materials	descriptive	clear cut areas 500m radius	present		birds	food/economics	1	2020	2020
energy/ raw materials	descriptive	dams presence	present		fish	conservation	1	2007	2007
energy/ raw materials	descriptive	dredging	present		herbaceous plants	exploratory	1	2020	2020
energy/ raw materials	descriptive	dredging and disposal areas	present	12	invertebrates	conservation	1	2014	2014
energy/ raw materials	descriptive	excavated area percent	present		birds	exploratory	1	2020	2020
energy/ raw materials	descriptive	extractive use areas	present		mammals	exploratory	1	2017	2017
energy/ raw materials	descriptive	forest processing facilities	present	15	invertebrates	invasions	1	2020	2020
energy/ raw materials	descriptive	hydropower stations	present	6	mammals	conservation	1	2018	2018
energy/ raw materials	descriptive	logged forest <25yrs old	present	15	mammals	exploratory	1	2021	2021
energy/ raw materials	descriptive	logged forest 25-200yrs old	present	15	mammals	exploratory	1	2021	2021
energy/ raw materials	descriptive	mines	present		mammals	conservation; exploratory	3	2013	2018

energy/ raw materials	descriptive	mines historic	present		fish	disturbance/habitat change	1	2018	2018
energy/ raw materials	descriptive	oil 3D seismic lines	present		mammals	invasions	1	2020	2020
energy/ raw materials	descriptive	oil cutlines	present		mammals	invasions; disturbance/habitat change	2	2018	2020
energy/ raw materials	descriptive	oil gas pipeline	present		fish	disturbance/habitat change	1	2018	2018
energy/ raw materials	descriptive	oil gas well pads conventional	present		fish	disturbance/habitat change	1	2018	2018
energy/ raw materials	descriptive	oil gas well pads unconventional	present		fish	disturbance/habitat change	1	2018	2018
energy/ raw materials	descriptive	oil gas well sites	present		mammals	disturbance/habitat change	2	2014	2018
energy/ raw materials	descriptive	oil pipelines	present		mammals	invasions; disturbance/habitat change	2	2018	2020
energy/ raw materials	descriptive	oil well sites	present		mammals	invasions	1	2020	2020
energy/ raw materials	descriptive	powerlines	present		mammals; birds	conservation; exploratory	3	2000	2018
energy/ raw materials	descriptive	renewable energy lease sites	present	7	invertebrates	conservation	1	2014	2014
energy/ raw materials	descriptive	reservoirs	present		birds	exploratory	1	2009	2009
energy/ raw materials	descriptive	reservoirs and dams	present		fish	invasions	1	2020	2020
energy/ raw materials	descriptive	roads seismic lines density	present		mammals	disturbance/habitat change	1	2015	2015
energy/ raw materials	descriptive	seismic lines	present		mammals	disturbance/habitat change	1	2014	2014
energy/ raw materials	descriptive	seismic lines 3D	present		mammals	disturbance/habitat change	1	2018	2018

energy/ raw materials	descriptive	seismic lines density 12k radius	present	mammals	exploratory	1	2012	2012
energy/ raw materials	descriptive	seismic lines density 5k radius	present	mammals	exploratory	1	2012	2012
energy/ raw materials	descriptive	seismic lines density 70m radius	present	mammals	exploratory	1	2012	2012
energy/ raw materials	descriptive	surface fuels type	present	amphibians; birds; mammals; reptiles	exploratory	1	2017	2017
energy/ raw materials	descriptive	transmission lines clearings	present	reptiles	disturbance/habitat change	1	2006	2006
energy/ raw materials	descriptive	unconventional oil and gas well pads	future	fish	disturbance/habitat change	1	2018	2018
energy/ raw materials	distance	artificial reservoir distance	present	reptiles	conflict/collisions	1	2021	2021
energy/ raw materials	distance	collection of bamboo shoots distance	present	mammals	disturbance/habitat change	1	2019	2019
energy/ raw materials	distance	collection of medicinal plants distance	present	mammals	disturbance/habitat change	1	2019	2019
energy/ raw materials	distance	dams distance	present	fish; invertebrates; birds	conservation; human health/safety	4	2013	2020
energy/ raw materials	distance	dams distance downstream	present	fish	reintroduction/restoration	1	2021	2021
energy/ raw materials	distance	dams downstream distance	present	invertebrates	exploratory	1	2015	2015
energy/ raw materials	distance	dams downstream mainstem distance	present	fish	conservation	1	2019	2019
energy/ raw materials	distance	dams mainstem upstream downstream distance	present	fish	conservation	1	2019	2019
energy/ raw materials	distance	dams upstream distance	present	invertebrates	exploratory	1	2015	2015

energy/ raw materials	distance	dams upstream mainstem distance	present		fish	conservation	1	2019	2019
energy/ raw materials	distance	electric lines distance	present		birds	exploratory; reintroduction/restoration	2	2020	2021
energy/ raw materials	distance	energy transmission lines distance	past; present	7	birds	disturbance/habitat change	1	2017	2017
energy/ raw materials	distance	extraction sites distance	present		amphibians	conservation	1	2018	2018
energy/ raw materials	distance	mines distance	present		mammals; birds	conservation; exploratory; disturbance/habitat change	6	2015	2021
energy/ raw materials	distance	mining clay distance	present		invertebrates	human health/safety	1	2018	2018
energy/ raw materials	distance	natural gas well distance	present		birds	conservation	1	2008	2008
energy/ raw materials	distance	oil camp distance	present		mammals; birds	conflict/collisions; food/economics	3	2011	2019
energy/ raw materials	distance	oil gas exploration extraction facility distance	present		mammals	disturbance/habitat change	1	2015	2015
energy/ raw materials	distance	oil gas wells distance	present		birds	food/economics	1	2011	2011
energy/ raw materials	distance	pipeline submarine cables extent	present		invertebrates	conservation	1	2014	2014
energy/ raw materials	distance	sand mining area distance	present		mammals	reintroduction/restoration	1	2021	2021
energy/ raw materials	distance	seismic lines distance	present		mammals	disturbance/habitat change	1	2015	2015
energy/ raw materials	distance	trails seismic roads pipeline main roads distance	present		mammals	disturbance/habitat change	1	2014	2014
energy/ raw materials	distance	transmission lines distance	present		birds	disturbance/habitat change; conservation; food/economics	3	2011	2018

energy/ raw materials	distance	truck to dripline distance	present		birds	reintroduction/restoration	1	2020	2020
energy/ raw materials	distance	utility features distance	present		herbaceous plants	invasions	1	2014	2014
energy/ raw materials	distance	well distance	present		mammals	conservation	1	2012	2012
energy/ raw materials	distance	wind farm distance	present	7	birds	exploratory	1	2017	2017
energy/ raw materials	distance	wind farmlands distance class	present	1	birds	disturbance/habitat change	1	2020	2020
energy/ raw materials	index	dams stream gradient heterogeneity	present; future		fish	disturbance/habitat change	1	2020	2020
energy/ raw materials	index	reservoir storage capacity	present; future		fish	disturbance/habitat change	1	2020	2020
energy/ raw materials	size	dams area size	present		birds	exploratory	1	2007	2007
energy/ raw materials	size	dams freeflowing fragment length	present		fish	conservation	1	2018	2018
energy/ raw materials	size	dams surface area size	present		fish	exploratory	1	2017	2017
energy/ raw materials	size	dams total segment network length	present		fish	conservation	1	2019	2019
energy/ raw materials	size	electric lines length	present		birds	exploratory	2	2005	2009
energy/ raw materials	size	forest dams edge length	present	15	birds	exploratory	1	2007	2007
energy/ raw materials	size	mines dump construction area	present		herbaceous plants	invasions	1	2017	2017
energy/ raw materials	size	powerlines length	present		birds	exploratory	1	2015	2015
energy/ raw materials	size	scrub dams edge length	present		birds	exploratory	1	2007	2007
energy/ raw materials	time	years since damming	present		fish	conservation	1	2007	2007

food/agriculture	density/count	abandoned cropland percent	present	trees/shrubs	exploratory	1	2020	2020
food/agriculture	density/count	abandoned pastures percent	present	trees/shrubs	exploratory	1	2020	2020
food/agriculture	density/count	abandoned pastures with rocks <30 percent	present	mammals	reintroduction/restoration	1	2003	2003
food/agriculture	density/count	abandoned pastures with rocks >30 percent	present	mammals	reintroduction/restoration	1	2003	2003
food/agriculture	density/count	agricultural areas 20km radius percent	present	mammals	exploratory	1	2020	2020
food/agriculture	density/count	agricultural areas count	present	birds	conservation	1	2017	2017
food/agriculture	density/count	agricultural areas count 100ha radius	present	invertebrates	exploratory	1	2009	2009
food/agriculture	density/count	agricultural areas density	present	mammals	conservation; disturbance/habitat change	4	2017	2021
food/agriculture	density/count	agricultural areas density 1000m radius	present	birds; mammals	invasions; exploratory	2	2017	2018
food/agriculture	density/count	agricultural areas density 2000m radius	present	mammals	exploratory	1	2017	2017
food/agriculture	density/count	agricultural areas density 250m radius	present	mammals	exploratory	1	2017	2017
food/agriculture	density/count	agricultural areas density 50m radius	present	birds	invasions	1	2018	2018
food/agriculture	density/count	agricultural areas largest patch percent 1000m radius	present	mammals	exploratory	1	2017	2017
food/agriculture	density/count	agricultural areas largest patch percent 2000m radius	present	mammals	exploratory	1	2017	2017
food/agriculture	density/count	agricultural areas largest patch percent 250m radius	present	mammals	exploratory	1	2017	2017
food/agriculture	density/count	agricultural areas largest patch percent 500m radius	present	mammals	exploratory	1	2017	2017

food/agriculture	density/count	agricultural areas or open area percent	present	mammals	invasions	1	2018	2018
food/agriculture	density/count	agricultural areas patch count 100ha radius	present	fish; invertebrates	conservation	1	2011	2011
food/agriculture	density/count	agricultural areas patch count 100ha radius 100m riparian radius	present	fish	exploratory	1	2009	2009
food/agriculture	density/count	agricultural areas patch count 100ha radius 100m upstream radius	present	fish	exploratory	1	2009	2009
food/agriculture	density/count	agricultural areas patch count 100ha radius subcatchment radius	present	fish	exploratory	1	2009	2009
food/agriculture	density/count	agricultural areas patch count 500m radius	present	mammals	conservation	1	2021	2021
food/agriculture	density/count	agricultural areas percent	past; present; future	mammals; herbaceous plants; birds; fish; amphibians; invertebrates; microorganisms; reptiles; trees/shrubs	reintroduction/restoration; disturbance/habitat change; exploratory; conservation; invasions; human health/safety; food/economics; conflict/collisions	92	2004	2021
food/agriculture	density/count	agricultural areas percent 1000m radius	present	amphibians; mammals; invertebrates	exploratory; conservation	3	2016	2020
food/agriculture	density/count	agricultural areas percent 100m radius	present	fish; amphibians; mammals	reintroduction/restoration; exploratory; conflict/collisions; disturbance/habitat change	4	2009	2021
food/agriculture	density/count	agricultural areas percent 100m upstream radius	present	fish	exploratory	1	2009	2009

food/agriculture	density/count	agricultural areas percent 10km radius	past; present	birds	disturbance/habitat change	1	2021	2021
food/agriculture	density/count	agricultural areas percent 1215m radius	past; present	birds	conservation	1	2021	2021
food/agriculture	density/count	agricultural areas percent 1500m radius	present	reptiles	conservation	1	2021	2021
food/agriculture	density/count	agricultural areas percent 165m radius	past; present	birds	conservation	1	2021	2021
food/agriculture	density/count	agricultural areas percent 1km radius	present	amphibians	disturbance/habitat change	1	2009	2009
food/agriculture	density/count	agricultural areas percent 2000m radius	present	amphibians; mammals	exploratory	2	2017	2020
food/agriculture	density/count	agricultural areas percent 200m radius	present	amphibians	exploratory	1	2020	2020
food/agriculture	density/count	agricultural areas percent 2500m radius	present	birds	invasions	1	2009	2009
food/agriculture	density/count	agricultural areas percent 250m radius	present	mammals	exploratory	1	2017	2017
food/agriculture	density/count	agricultural areas percent 2km radius	present	amphibians	disturbance/habitat change	1	2009	2009
food/agriculture	density/count	agricultural areas percent 300m radius	present	reptiles	conservation	1	2021	2021
food/agriculture	density/count	agricultural areas percent 30m radius	present	amphibians	disturbance/habitat change	1	2009	2009
food/agriculture	density/count	agricultural areas percent 315m radius	past; present	birds	conservation	1	2021	2021
food/agriculture	density/count	agricultural areas percent 3km radius	present	birds	reintroduction/restoration	1	2017	2017
food/agriculture	density/count	agricultural areas percent 4km radius	present	birds	reintroduction/restoration	1	2018	2018
food/agriculture	density/count	agricultural areas percent 5000m radius	present	amphibians	exploratory	1	2020	2020

food/agriculture	density/count	agricultural areas percent 500m radius	present		amphibians; mammals; herbaceous plants	exploratory; conflict/collisions; invasions; disturbance/habitat change	5	2009	2020
food/agriculture	density/count	agricultural areas percent 50m radius	present		birds	invasions	1	2009	2009
food/agriculture	density/count	agricultural areas percent 615m radius	past; present		birds	conservation	1	2021	2021
food/agriculture	density/count	agricultural areas percent catchment	present		fish	disturbance/habitat change	1	2014	2014
food/agriculture	density/count	agricultural areas percent local	present		invertebrates	conservation	1	2015	2015
food/agriculture	density/count	agricultural areas percent local watershed	present		fish	conservation	1	2020	2020
food/agriculture	density/count	agricultural areas percent subcatchment radius	present		fish	exploratory	1	2009	2009
food/agriculture	density/count	agricultural areas percent upstream watershed	present		fish	conservation	1	2020	2020
food/agriculture	density/count	agricultural areas percent watershed	present		herbaceous plants	invasions	1	2010	2010
food/agriculture	density/count	agricultural areas percent watershed mean	present		herbaceous plants	invasions	1	2010	2010
food/agriculture	density/count	agricultural areas percent whole	present		invertebrates	conservation	1	2015	2015
food/agriculture	density/count	agricultural areas use percent	present		fish	invasions	1	2015	2015
food/agriculture	density/count	agricultural areas using agrochemicals count	present		herbaceous plants	food/economics	1	2018	2018
food/agriculture	density/count	agricultural areas using fertilizers count	present		herbaceous plants	food/economics	1	2018	2018
food/agriculture	density/count	agricultural areas with irrigation count	present	6	herbaceous plants	food/economics	1	2018	2018

food/agriculture	density/count	agricultural areas with natural vegetation percent	present; future		invertebrates; herbaceous plants; birds	disturbance/habitat change; reintroduction/restoration	3	2012	2021
food/agriculture	density/count	agricultural areas with pest control and/or plant diseases count	present	3	herbaceous plants	food/economics	1	2018	2018
food/agriculture	density/count	agricultural areas with water resources count	present	01; 06	herbaceous plants	food/economics	1	2018	2018
food/agriculture	density/count	agricultural grasslands density	present		mammals	food/economics	1	2015	2015
food/agriculture	density/count	agricultural grasslands percent	present		invertebrates	reintroduction/restoration	1	2019	2019
food/agriculture	density/count	agricultural machinery equipment in family agricultural areas count	present		herbaceous plants	food/economics	1	2018	2018
food/agriculture	density/count	agricultural machinery equipment in non-family agricultural areas count	present		herbaceous plants	food/economics	1	2018	2018
food/agriculture	density/count	agricultural natural mosaic percent	present		birds	exploratory	1	2013	2013
food/agriculture	density/count	agricultural rural area percent	present		invertebrates	human health/safety	1	2018	2018
food/agriculture	density/count	agroforestry areas density	present	15	mammals	conservation	1	2016	2016
food/agriculture	density/count	agroforestry areas percent	present	15	mammals	conservation	2	2016	2018
food/agriculture	density/count	agroforestry percent catchment	present	15	fish	reintroduction/restoration	1	2014	2014
food/agriculture	density/count	allotments percent	present		invertebrates	conservation	1	2017	2017
food/agriculture	density/count	annual fish stock events count	present		invertebrates	invasions	1	2021	2021
food/agriculture	density/count	arable and horticultural land percent	present		invertebrates	human health/safety	1	2019	2019
food/agriculture	density/count	arable horticulture frequency	present		mammals	reintroduction/restoration	1	2021	2021
food/agriculture	density/count	arable land and cropland percent	past	15	birds	conservation	1	2021	2021

food/agriculture	density/count	arable land and meadows favorable percent	present	15	invertebrates	disturbance/habitat change	1	2009	2009
food/agriculture	density/count	arable land density	present	15	mammals	human health/safety	1	2014	2014
food/agriculture	density/count	arable land dry percent	present	15	birds	disturbance/habitat change	1	2004	2004
food/agriculture	density/count	arable land frequency	present	15	mammals	exploratory	1	2009	2009
food/agriculture	density/count	arable land irrigated percent	present	15	birds	disturbance/habitat change	1	2004	2004
food/agriculture	density/count	arable land non-irrigated percent	present	15	birds	reintroduction/restoration	1	2012	2012
food/agriculture	density/count	arable land percent	past; present; future	15	birds; invertebrates; mammals; fish	exploratory; disturbance/habitat change; reintroduction/restoration; conflict/collisions; conservation; food/economics	16	2001	2021
food/agriculture	density/count	arable land percent 1km radius	present	15	birds	disturbance/habitat change	1	2013	2013
food/agriculture	density/count	artificial pastures percent	present		amphibians	conservation	1	2015	2015
food/agriculture	density/count	aviculture frequency	present		mammals	invasions	1	2013	2013
food/agriculture	density/count	banana plantation percent	present		amphibians	conservation	1	2015	2015
food/agriculture	density/count	broadleaved deciduous orchards shrubs percent	present		amphibians	exploratory	1	2015	2015
food/agriculture	density/count	complex cultivation patterns percent	present		mammals; herbaceous plants; birds	reintroduction/restoration; disturbance/habitat change; exploratory	4	2012	2020
food/agriculture	density/count	corn field percent	present		birds	conservation	1	2018	2018
food/agriculture	density/count	crop alfalfa percent	present		birds	conservation; exploratory	4	2009	2019
food/agriculture	density/count	crop almond crops percent	present	2	birds	exploratory	1	2018	2018
food/agriculture	density/count	crop almond percent	present		reptiles	exploratory	1	2014	2014
food/agriculture	density/count	crop and natural mosaic percent	present		birds; mammals	food/economics	1	2007	2007

food/agriculture	density/count	crop artichoke frequency	present		birds	disturbance/habitat change	1	2011	2011
food/agriculture	density/count	crop artichokes harvested frequency	present		birds	disturbance/habitat change	1	2011	2011
food/agriculture	density/count	crop barley percent	present		birds	exploratory	1	2018	2018
food/agriculture	density/count	crop cereal density	present		mammals	food/economics	1	2015	2015
food/agriculture	density/count	crop cereal dry percent	present		birds	conservation	1	2006	2006
food/agriculture	density/count	crop cereal percent	present		reptiles; birds	exploratory; conservation	2	2009	2014
food/agriculture	density/count	crop density	present		mammals	reintroduction/restoration	1	2016	2016
food/agriculture	density/count	crop dry heterogeneous percent	present		birds; mammals	food/economics	1	2007	2007
food/agriculture	density/count	crop dry percent	present		reptiles; mammals	conflict/collisions; reintroduction/restoration	2	2018	2021
food/agriculture	density/count	crop fruit percent	present		invertebrates	disturbance/habitat change	1	2019	2019
food/agriculture	density/count	crop grain percent	present; future		invertebrates	exploratory	1	2021	2021
food/agriculture	density/count	crop grape percent	present		birds	exploratory	1	2018	2018
food/agriculture	density/count	crop maize 250m of rivers percent	present; future	6	birds	food/economics	1	2014	2014
food/agriculture	density/count	crop maize density	present		mammals	food/economics	1	2015	2015
food/agriculture	density/count	crop maize percent	present; future		mammals; birds	conflict/collisions; reintroduction/restoration; food/economics; exploratory	5	2006	2020
food/agriculture	density/count	crop oilseed rape density	present		mammals	food/economics	1	2015	2015
food/agriculture	density/count	crop oilseed rape percent	present; future		invertebrates; birds	exploratory; food/economics	3	2011	2021
food/agriculture	density/count	crop percent	present		invertebrates	exploratory	1	2017	2017
food/agriculture	density/count	crop potato percent	present; future		invertebrates	exploratory	1	2021	2021
food/agriculture	density/count	crop safflower percent	present		birds	exploratory	1	2018	2018
food/agriculture	density/count	crop soybean field percent	present		birds	conservation	1	2018	2018
food/agriculture	density/count	crop specialized percent	present		birds	exploratory	1	2011	2011

food/agriculture	density/count	crop spring grain percent	present; future	birds	food/economics; exploratory	2	2011	2014
food/agriculture	density/count	crop strawberry percent	present	birds	exploratory	1	2018	2018
food/agriculture	density/count	crop sugar beet percent	present; future	invertebrates	exploratory	1	2021	2021
food/agriculture	density/count	crop sugar cane percent	present	amphibians	conservation	1	2015	2015
food/agriculture	density/count	crop walnut percent	present	birds	exploratory	1	2018	2018
food/agriculture	density/count	crop wheat frequency	present	birds	disturbance/habitat change	1	2011	2011
food/agriculture	density/count	crop wheat percent	present	fish	disturbance/habitat change	1	2015	2015
food/agriculture	density/count	cropland and natural mosaic percent	present	invertebrates	human health/safety	1	2017	2017
food/agriculture	density/count	cropland and vegetation mosaic percent	present; future	invertebrates; birds	human health/safety; disturbance/habitat change	2	2012	2016
food/agriculture	density/count	cropland count	present; future	birds	conservation; disturbance/habitat change	2	2014	2014
food/agriculture	density/count	cropland density	present	mammals; fish	conservation; disturbance/habitat change	2	2013	2016
food/agriculture	density/count	cropland dry grass percent	present	birds	disturbance/habitat change	1	2013	2013
food/agriculture	density/count	cropland dry percent	present	birds	disturbance/habitat change	2	2004	2013
food/agriculture	density/count	cropland grassland percent	present	birds	conservation	1	2014	2014
food/agriculture	density/count	cropland irrigated percent	present	mammals; birds	exploratory; disturbance/habitat change	2	2004	2012
food/agriculture	density/count	cropland mosaic density	present	birds	conservation	1	2019	2019
food/agriculture	density/count	cropland mosaic percent	present	invertebrates; mammals	human health/safety; exploratory	3	2012	2017

food/agriculture	density/count	cropland natural vegetation mosaic percent	present		mammals	exploratory	1	2021	2021
food/agriculture	density/count	cropland other density	present		mammals	food/economics	1	2015	2015
food/agriculture	density/count	cropland pastures areas percent	present		birds	invasions	1	2014	2014
food/agriculture	density/count	cropland percent	past; present; future		fish; mammals; herbaceous plants; trees/shrubs; invertebrates; amphibians; birds; reptiles	conservation; conflict/collisions; exploratory; invasions; reintroduction/restoration; human health/safety; disturbance/habitat change; food/economics	44	2003	2021
food/agriculture	density/count	cropland percent 100m radius	present		invertebrates	exploratory	2	2015	2015
food/agriculture	density/count	cropland percent 10m radius	present		invertebrates	exploratory	2	2015	2015
food/agriculture	density/count	cropland percent 2km radius	present		amphibians	exploratory	1	2012	2012
food/agriculture	density/count	cropland rainfed percent	present		mammals	exploratory	1	2012	2012
food/agriculture	density/count	crops annual C3 percent	present; future	2	invertebrates	human health/safety	2	2019	2019
food/agriculture	density/count	crops annual C4 percent	present; future	2	invertebrates	human health/safety	1	2019	2019
food/agriculture	density/count	crops annual percent	present; future	2	trees/shrubs; birds	invasions; disturbance/habitat change	3	2013	2021
food/agriculture	density/count	crops autumn-sown percent 1500m radius	present	2	birds	conservation	1	2020	2020
food/agriculture	density/count	crops dryland percent	present	2	mammals	reintroduction/restoration	1	2006	2006
food/agriculture	density/count	crops nitrogen-fixing C3 percent	present; future	2	invertebrates	human health/safety	1	2019	2019
food/agriculture	density/count	crops other percent	present	2	mammals	reintroduction/restoration	2	2018	2019
food/agriculture	density/count	crops percent	present	2	mammals	reintroduction/restoration	1	2020	2020

food/agriculture	density/count	crops percent 1000m radius	present	2	mammals	disturbance/habitat change	1	2019	2019
food/agriculture	density/count	crops percent 500m radius	present	2	mammals	disturbance/habitat change	1	2019	2019
food/agriculture	density/count	crops perennial C3 percent	present; future	2	invertebrates	human health/safety	1	2019	2019
food/agriculture	density/count	crops perennial C4 percent	present; future	2	invertebrates	human health/safety	1	2019	2019
food/agriculture	density/count	crops permanent percent	present	2	mammals	reintroduction/restoration	1	2020	2020
food/agriculture	density/count	crops rainfed percent	present	2	mammals	conservation	1	2018	2018
food/agriculture	density/count	cultivated areas frequency	present		mammals	conservation	1	2020	2020
food/agriculture	density/count	cultivated areas percent	present; future		birds; fish; microorganisms; mammals; herbaceous plants; invertebrates	exploratory; reintroduction/restoration; human health/safety; conservation; disturbance/habitat change	13	2004	2021
food/agriculture	density/count	cultivated areas percent 100m radius	present		fish	reintroduction/restoration	1	2021	2021
food/agriculture	density/count	cultivated areas percent 1km radius	present		fish	reintroduction/restoration	1	2021	2021
food/agriculture	density/count	cultivated crop percent	present		birds	conservation	1	2019	2019
food/agriculture	density/count	cultivated cropland percent	present		birds	exploratory	2	2020	2020
food/agriculture	density/count	cultivated crops percent	present	2	invertebrates	disturbance/habitat change	1	2019	2019
food/agriculture	density/count	cultivated crops percent catchment	present	2	fish; invertebrates	reintroduction/restoration; disturbance/habitat change	2	2017	2021
food/agriculture	density/count	cultivated crops stream segment percent	present	2	invertebrates	disturbance/habitat change	1	2017	2017
food/agriculture	density/count	cultivated herbaceous crops percent	present	2	amphibians	exploratory	1	2015	2015
food/agriculture	density/count	cultivated woody crops percent	present	2	amphibians	exploratory	1	2015	2015

food/agriculture	density/count	cultivation and natural mosaic percent	present		mammals	reintroduction/restoration	1	2020	2020
food/agriculture	density/count	donkey density	present		invertebrates	human health/safety	1	2019	2019
food/agriculture	density/count	dry wood crops percent	present	2	birds; mammals	food/economics	1	2007	2007
food/agriculture	density/count	dryland agricultural areas percent	present		trees/shrubs	disturbance/habitat change	1	2014	2014
food/agriculture	density/count	extensive agricultural areas percent	present		birds; herbaceous plants; invertebrates	exploratory	1	2011	2011
food/agriculture	density/count	fallow and field margin percent	present		birds	reintroduction/restoration	1	2011	2011
food/agriculture	density/count	fallow cropland percent	present		birds	exploratory	1	2018	2018
food/agriculture	density/count	fallow land frequency	present		birds	disturbance/habitat change	1	2011	2011
food/agriculture	density/count	fallow land percent	present; future		birds; invertebrates	conservation; food/economics	3	2013	2018
food/agriculture	density/count	farm density 1500m radius	present		birds	conservation	1	2020	2020
food/agriculture	density/count	farm pond density	present		fish	conservation	1	2019	2019
food/agriculture	density/count	farmands percent	past; present		herbaceous plants	invasions	1	2017	2017
food/agriculture	density/count	farmers 35-50yrs old count	present		invertebrates	exploratory	1	2006	2006
food/agriculture	density/count	farmers full-time count	present		invertebrates	exploratory	1	2006	2006
food/agriculture	density/count	farmers part-time count	present		invertebrates	exploratory	1	2006	2006
food/agriculture	density/count	farmers percent 1500m radius	present		birds	conservation	1	2020	2020
food/agriculture	density/count	farming dry frequency	present		birds	conservation	1	2015	2015
food/agriculture	density/count	farming percent yr2000 100m radius	present		herbaceous plants	invasions	1	2012	2012
food/agriculture	density/count	farming percent yr2000 500m radius	present		herbaceous plants	invasions	1	2012	2012
food/agriculture	density/count	farmlands density	past; present		mammals	disturbance/habitat change	1	2015	2015

food/agriculture	density/count	farmlands in watershed percent	present		microorganisms	disturbance/habitat change	1	2015	2015
food/agriculture	density/count	farmlands percent	past; present; future		mammals; fish; birds; amphibians; herbaceous plants	disturbance/habitat change; exploratory; reintroduction/restoration; conservation; conflict/collisions; invasions; human health/safety	11	2014	2021
food/agriculture	density/count	farmlands percent 200m radius	present		microorganisms	disturbance/habitat change	1	2015	2015
food/agriculture	density/count	farmlands percent 9km radius	present		mammals	exploratory	1	2021	2021
food/agriculture	density/count	farms in agricultural areas >20ha count	present		invertebrates	exploratory	1	2006	2006
food/agriculture	density/count	farms in agricultural areas 0-5ha count	present		invertebrates	exploratory	1	2006	2006
food/agriculture	density/count	farms in agricultural areas 10-20ha count	present		invertebrates	exploratory	1	2006	2006
food/agriculture	density/count	farms in agricultural areas 5-10ha count	present		invertebrates	exploratory	1	2006	2006
food/agriculture	density/count	fertilizer nitrogen application rate mean	present	2	invertebrates	conservation	1	2021	2021
food/agriculture	density/count	fertilizer tons per hectare lime	present	2	birds	disturbance/habitat change	1	2014	2014
food/agriculture	density/count	fertilizer tons per hectare nitrogen	present	2	birds	disturbance/habitat change	1	2014	2014
food/agriculture	density/count	fertilizer tons per hectare phosphate	present	2	birds	disturbance/habitat change	1	2014	2014
food/agriculture	density/count	fertilizer tons per hectare potassium	present	2	birds	disturbance/habitat change	1	2014	2014
food/agriculture	density/count	fields percent	present		mammals	reintroduction/restoration	1	2018	2018
food/agriculture	density/count	fishing total abundance fish caught	present	14	fish	food/economics	1	2018	2018

food/agriculture	density/count	forest agricultural areas edge density	present	15	birds	exploratory	1	2011	2011
food/agriculture	density/count	forest plantation percent	present	15	herbaceous plants	invasions	1	2016	2016
food/agriculture	density/count	grazing areas percent	present		birds; herbaceous plants	conflict/collisions; exploratory; invasions	3	2013	2015
food/agriculture	density/count	grazing areas sum	present		birds	conservation	1	2014	2014
food/agriculture	density/count	grazing percent	past; present		fish; mammals	conservation; conflict/collisions	2	2013	2021
food/agriculture	density/count	grazing percent 1km radius	present		herbaceous plants	invasions	1	2019	2019
food/agriculture	density/count	grazing sheep alpine percent	present		invertebrates	disturbance/habitat change	1	2009	2009
food/agriculture	density/count	harvest <10yrs percent 1km radius	present		mammals	disturbance/habitat change	1	2018	2018
food/agriculture	density/count	harvest heavy <10yrs percent 1km radius	present		mammals	disturbance/habitat change	1	2018	2018
food/agriculture	density/count	harvested forest percent	present	15	reptiles	conflict/collisions	1	2021	2021
food/agriculture	density/count	harvested forest regenerating percent	present	15	mammals	exploratory	1	2014	2014
food/agriculture	density/count	harvesters count	present		birds	disturbance/habitat change	1	2014	2014
food/agriculture	density/count	hay count	present; future		birds	disturbance/habitat change	1	2014	2014
food/agriculture	density/count	hay crops percent	present	2	birds	exploratory	1	2018	2018
food/agriculture	density/count	hayfields percent	present		invertebrates	conservation	1	2017	2017
food/agriculture	density/count	herbaceous crops with oaks percent	present	2	birds; mammals	food/economics	1	2007	2007
food/agriculture	density/count	hiding spots from fishing percent	present	14	fish	exploratory	1	2021	2021
food/agriculture	density/count	horse density	present		invertebrates; mammals	human health/safety; disturbance/habitat change	2	2017	2019

food/agriculture	density/count	intensive agricultural areas percent	present		birds; herbaceous plants; invertebrates	exploratory	1	2011	2011
food/agriculture	density/count	irrigated agricultural areas percent	present		birds; trees/shrubs	conservation; disturbance/habitat change	3	2014	2017
food/agriculture	density/count	irrigated areas percent	present		mammals; invertebrates; birds	food/economics; human health/safety; exploratory	3	2005	2014
food/agriculture	density/count	irrigated crops percent	present	2	mammals	conservation; reintroduction/restoration	2	2018	2018
food/agriculture	density/count	irrigated crops percent catchment	present	2	fish	reintroduction/restoration	1	2014	2014
food/agriculture	density/count	irrigated cultures percent	present		birds	conservation	1	2003	2003
food/agriculture	density/count	irrigated herbaceous crops percent	present	2	birds; mammals	food/economics	1	2007	2007
food/agriculture	density/count	irrigated heterogeneous crops percent	present	2	birds; mammals	food/economics	1	2007	2007
food/agriculture	density/count	irrigated intensive agricultural areas percent	present		mammals	exploratory	1	2020	2020
food/agriculture	density/count	irrigated woody crops percent	present	2	birds; mammals	food/economics	1	2007	2007
food/agriculture	density/count	livestock cattle density	present; future		invertebrates; trees/shrubs; mammals; microorganisms; birds	human health/safety; invasions; conflict/collisions; exploratory; reintroduction/restoration; conservation; disturbance/habitat change; food/economics	19	2009	2021
food/agriculture	density/count	livestock cattle farm percent	present		birds	exploratory	1	2007	2007
food/agriculture	density/count	livestock cattle percent 1km radius	present		birds	reintroduction/restoration	1	2009	2009

food/agriculture	density/count	livestock cattle percent 2.5km radius	present	birds	reintroduction/restoration	1	2009	2009
food/agriculture	density/count	livestock cattle percent 5km radius	present	birds	reintroduction/restoration	1	2009	2009
food/agriculture	density/count	livestock count	present	invertebrates	exploratory	1	2006	2006
food/agriculture	density/count	livestock count 1km radius	present	birds	reintroduction/restoration	1	2009	2009
food/agriculture	density/count	livestock count 2.5km radius	present	birds	reintroduction/restoration	1	2009	2009
food/agriculture	density/count	livestock count 5km radius	present	birds	reintroduction/restoration	1	2009	2009
food/agriculture	density/count	livestock deer density	present	microorganisms	food/economics	1	2016	2016
food/agriculture	density/count	livestock density	past; present	mammals; fish; invertebrates; birds	disturbance/habitat change; exploratory; conservation; reintroduction/restoration; human health/safety; conflict/collisions	13	2013	2021
food/agriculture	density/count	livestock density annual	present	mammals	conflict/collisions	1	2018	2018
food/agriculture	density/count	livestock fodder percent	present	birds	conservation	1	2009	2009
food/agriculture	density/count	livestock goat density	present	invertebrates; mammals	human health/safety; conflict/collisions	3	2011	2019
food/agriculture	density/count	livestock pig density	present	mammals	conflict/collisions; reintroduction/restoration; human health/safety; food/economics	4	2014	2021
food/agriculture	density/count	livestock sheep density	present	invertebrates; mammals; birds	human health/safety; reintroduction/restoration; conflict/collisions; food/economics	7	2011	2019
food/agriculture	density/count	livestock sheep goat density	present; future	birds; mammals; microorganisms	reintroduction/restoration; conflict/collisions; food/economics; exploratory	4	2015	2021
food/agriculture	density/count	livestock sheep percent 1km radius	present	birds	reintroduction/restoration	1	2009	2009

food/agriculture	density/count	livestock sheep percent 2.5km radius	present		birds	reintroduction/restoration	1	2009	2009
food/agriculture	density/count	livestock sheep percent 5km radius	present		birds	reintroduction/restoration	1	2009	2009
food/agriculture	density/count	meadow mowed percent 1km radius	present		birds	disturbance/habitat change	1	2013	2013
food/agriculture	density/count	meadows and pastures percent	present; future		herbaceous plants	disturbance/habitat change	2	2010	2015
food/agriculture	density/count	nana plantation percent	present		amphibians	conservation	1	2015	2015
food/agriculture	density/count	non-irrigated arable land percent	present	15	herbaceous plants; birds	disturbance/habitat change; exploratory	2	2013	2015
food/agriculture	density/count	non-irrigated crops percent catchment	present	2	fish	reintroduction/restoration	1	2014	2014
food/agriculture	density/count	non-irrigated cultures percent	present		birds	conservation	1	2003	2003
food/agriculture	density/count	oat crops percent	present	2	birds	exploratory	1	2018	2018
food/agriculture	density/count	orchards and hedgerows percent	present		invertebrates	disturbance/habitat change	1	2009	2009
food/agriculture	density/count	orchards and vineyards frequency	present		mammals	exploratory	1	2012	2012
food/agriculture	density/count	orchards and vineyards percent	present		birds	exploratory	1	2020	2020
food/agriculture	density/count	orchards broadleaved deciduous percent	present		amphibians	exploratory	1	2015	2015
food/agriculture	density/count	orchards broadleaved evergreen percent	present		amphibians	exploratory	1	2015	2015
food/agriculture	density/count	orchards fruit tree percent	present; future	15	reptiles; invertebrates	conflict/collisions; exploratory	2	2021	2021
food/agriculture	density/count	orchards needle-leaved evergreen percent	present		amphibians	exploratory	1	2015	2015
food/agriculture	density/count	orchards olives frequency	present		mammals	conservation	1	2020	2020
food/agriculture	density/count	orchards olives percent	present		birds; reptiles	exploratory; conservation; reintroduction/restoration	5	2014	2021

food/agriculture	density/count	orchards other percent	present		invertebrates	disturbance/habitat change	1	2009	2009
food/agriculture	density/count	orchards percent	past; present; future		birds; amphibians; herbaceous plants	disturbance/habitat change; exploratory; conservation	7	2010	2021
food/agriculture	density/count	orchards percent 1km radius	present		birds	disturbance/habitat change	1	2013	2013
food/agriculture	density/count	orchards percent 2500m radius	present		birds	invasions	1	2009	2009
food/agriculture	density/count	orchards percent 50m radius	present		birds	invasions	1	2009	2009
food/agriculture	density/count	orchards tree density	present	15	birds	conservation	1	2020	2020
food/agriculture	density/count	pastures 10yrs ago percent 10km radius	past; present		birds	disturbance/habitat change	1	2021	2021
food/agriculture	density/count	pastures 10yrs ago percent 30km radius	past; present		birds	disturbance/habitat change	1	2021	2021
food/agriculture	density/count	pastures alpine percent	present		mammals	conservation	1	2019	2019
food/agriculture	density/count	pastures and grassland percent	present; future		birds	food/economics	1	2014	2014
food/agriculture	density/count	pastures and grasslands percent	present		mammals	reintroduction/restoration	1	2020	2020
food/agriculture	density/count	pastures and hay percent	present		birds	exploratory	2	2017	2020
food/agriculture	density/count	pastures and hay percent catchment	present		fish; invertebrates	reintroduction/restoration; conservation; disturbance/habitat change	3	2017	2021
food/agriculture	density/count	pastures and hay stream segment percent	present		invertebrates	disturbance/habitat change	1	2017	2017
food/agriculture	density/count	pastures and meadows alpine percent	present		invertebrates	disturbance/habitat change	1	2009	2009
food/agriculture	density/count	pastures and meadows overgrown percent	present		invertebrates	disturbance/habitat change	1	2009	2009

food/agriculture	density/count	pastures and meadows percent	present	birds	reintroduction/restoration	1	2021	2021
food/agriculture	density/count	pastures and meadows percent 1km radius	present	birds	disturbance/habitat change	1	2013	2013
food/agriculture	density/count	pastures and shrub edge density	present	mammals	conservation	1	2006	2006
food/agriculture	density/count	pastures count 100ha radius	present	invertebrates	exploratory	1	2009	2009
food/agriculture	density/count	pastures density	present	mammals	conservation; human health/safety	2	2014	2016
food/agriculture	density/count	pastures favorable percent	present	invertebrates	disturbance/habitat change	1	2009	2009
food/agriculture	density/count	pastures frequency	present	mammals	conservation	1	2020	2020
food/agriculture	density/count	pastures or hay percent	past; present	fish	conservation	1	2021	2021
food/agriculture	density/count	pastures patch count 100ha radius	present	fish; invertebrates	conservation	1	2011	2011
food/agriculture	density/count	pastures patch count 100ha radius 100m riparian radius	present	fish	exploratory	1	2009	2009
food/agriculture	density/count	pastures patch count 100ha radius 100m upstream radius	present	fish	exploratory	1	2009	2009
food/agriculture	density/count	pastures patch count 100ha radius subcatchment radius	present	fish	exploratory	1	2009	2009
food/agriculture	density/count	pastures percent	past; present; future	birds; mammals; reptiles; invertebrates; herbaceous plants; microorganisms; amphibians; trees/shrubs; fish	conservation; invasions; conflict/collisions; exploratory; disturbance/habitat change; reintroduction/restoration; human health/safety; food/economics	41	2003	2021

food/agriculture	density/count	pastures percent 100m radius	present	invertebrates	exploratory	2	2015	2015
food/agriculture	density/count	pastures percent 100m riparian radius	present	fish	exploratory	1	2009	2009
food/agriculture	density/count	pastures percent 100m upstream radius	present	fish	exploratory	1	2009	2009
food/agriculture	density/count	pastures percent 10km radius	present	mammals	conservation	1	2013	2013
food/agriculture	density/count	pastures percent 10m radius	present	invertebrates	exploratory	2	2015	2015
food/agriculture	density/count	pastures percent 1km radius	present	mammals; birds	conservation; reintroduction/restoration; food/economics	3	2009	2013
food/agriculture	density/count	pastures percent 2.5km radius	present	birds	reintroduction/restoration	1	2009	2009
food/agriculture	density/count	pastures percent 30km radius	past; present	birds	disturbance/habitat change	1	2021	2021
food/agriculture	density/count	pastures percent 4km radius	present	mammals	conservation	1	2013	2013
food/agriculture	density/count	pastures percent 5km radius	present	birds	reintroduction/restoration	1	2009	2009
food/agriculture	density/count	pastures percent 7km radius	present	mammals	conservation	1	2013	2013
food/agriculture	density/count	pastures percent subcatchment radius	present	fish	exploratory	1	2009	2009
food/agriculture	density/count	pastures shrub edge density	present	mammals	conservation	1	2003	2003
food/agriculture	density/count	pastures stony percent	present	invertebrates	disturbance/habitat change	1	2009	2009
food/agriculture	density/count	pastures with conifers percent	present	birds; mammals	food/economics	1	2007	2007
food/agriculture	density/count	pastures with oaks percent	present	birds; mammals	food/economics	1	2007	2007
food/agriculture	density/count	percent agricultural 2.5km radius	present	mammals	disturbance/habitat change	1	2015	2015

food/agriculture	density/count	permanent crops percent	present; future	2	invertebrates; fish	exploratory; disturbance/habitat change	6	2013	2021
food/agriculture	density/count	permanent cultures percent	present		birds	reintroduction/restoration	1	2021	2021
food/agriculture	density/count	pine plantation percent	present		trees/shrubs	invasions	1	2020	2020
food/agriculture	density/count	pine plantation percent 10km radius	present		mammals	conservation	1	2013	2013
food/agriculture	density/count	pine plantation percent 1km radius	present		mammals	conservation	1	2013	2013
food/agriculture	density/count	pine plantation percent 4km radius	present		mammals	conservation	1	2013	2013
food/agriculture	density/count	pine plantation percent 7km radius	present		mammals	conservation	1	2013	2013
food/agriculture	density/count	plantation percent	present		herbaceous plants	disturbance/habitat change	1	2015	2015
food/agriculture	density/count	platanus trees density 1000m radius	present		birds	invasions	1	2018	2018
food/agriculture	density/count	platanus trees density 500m radius	present		birds	invasions	1	2018	2018
food/agriculture	density/count	platanus trees density 50m radius	present		birds	invasions	1	2018	2018
food/agriculture	density/count	ploughed fields percent	present		birds	exploratory	1	2006	2006
food/agriculture	density/count	poplar cultivations percent	past; present; future		birds	disturbance/habitat change	1	2010	2010
food/agriculture	density/count	poultry density	present		mammals; birds	conflict/collisions; conservation	2	2017	2021
food/agriculture	density/count	poultry farms density	present		mammals	food/economics	1	2014	2014
food/agriculture	density/count	precipitation driest corrected irrigation	present	6	invertebrates	exploratory	1	2013	2013
food/agriculture	density/count	precipitation evaporation corrected irrigation	present	6	invertebrates	exploratory	1	2013	2013
food/agriculture	density/count	productive lands without trees percent	present		mammals	disturbance/habitat change	1	2019	2019

	food/agriculture	density/count	rainfed cropland density	present		birds	conservation	1	2019	2019
	food/agriculture	density/count	rainfed cropland percent	present		invertebrates	human health/safety	1	2016	2016
	food/agriculture	density/count	rangeland	past; present		mammals; birds; reptiles	exploratory; conservation	4	2018	2021
	food/agriculture	density/count	rangeland and agricultural areas percent	present		mammals	conservation	1	2018	2018
	food/agriculture	density/count	rangeland percent	present; future		mammals; amphibians; birds; reptiles; herbaceous plants; invertebrates	disturbance/habitat change; invasions; human health/safety	6	2018	2021
	food/agriculture	density/count	recently harvested area percent	present		birds	exploratory	1	2008	2008
	food/agriculture	density/count	rice paddy percent	present		reptiles; birds; mammals; herbaceous plants; invertebrates	conflict/collisions; reintroduction/restoration; conservation; disturbance/habitat change; food/economics; exploratory	9	2012	2021
	food/agriculture	density/count	root crops percent	present; future	2	birds	food/economics; exploratory	2	2011	2014
	food/agriculture	density/count	row crop agricultural areas percent	present		fish; invertebrates	conservation	1	2018	2018
	food/agriculture	density/count	row crops percent	present	2	fish	exploratory; disturbance/habitat change	2	2015	2019
	food/agriculture	density/count	small farms percent 10km radius	present		mammals	conservation	1	2013	2013
	food/agriculture	density/count	small farms percent 1km radius	present		mammals	conservation	1	2013	2013
	food/agriculture	density/count	small farms percent 4km radius	present		mammals	conservation	1	2013	2013
	food/agriculture	density/count	small farms percent 7km radius	present		mammals	conservation	1	2013	2013

food/agriculture	density/count	small ruminants density	present		mammals	conflict/collisions; food/economics; conservation	3	2013	2021
food/agriculture	density/count	spring crops count	present	2	herbaceous plants	exploratory	1	2013	2013
food/agriculture	density/count	spring tillage operations count	present		herbaceous plants	exploratory	1	2013	2013
food/agriculture	density/count	tea plantation percent	present; future		mammals; invertebrates	conservation; disturbance/habitat change	2	2015	2020
food/agriculture	density/count	tilled field frequency	present		birds	disturbance/habitat change	1	2011	2011
food/agriculture	density/count	tractors count	present		birds; invertebrates	disturbance/habitat change; exploratory	2	2006	2014
food/agriculture	density/count	tree nursery percent	present	15	amphibians	conservation	1	2015	2015
food/agriculture	density/count	trees on agricultural areas percent	present		invertebrates	disturbance/habitat change	1	2009	2009
food/agriculture	density/count	unconverted maize percent	present; future		birds	food/economics	1	2014	2014
food/agriculture	density/count	uncultivated areas percent	present		birds	reintroduction/restoration	1	2014	2014
food/agriculture	density/count	unregulated agricultural areas percent	present; future		invertebrates	exploratory	1	2021	2021
food/agriculture	density/count	untilled areas percent	present		birds	conservation	1	2009	2009
food/agriculture	density/count	vineyards and orchards percent	present		birds	exploratory	1	2014	2014
food/agriculture	density/count	vineyards frequency	present		mammals	conservation	1	2007	2007
food/agriculture	density/count	vineyards or pomiculture percent	present		mammals	invasions	1	2018	2018
food/agriculture	density/count	vineyards percent	past; present; future		birds; herbaceous plants; invertebrates	disturbance/habitat change; exploratory; reintroduction/restoration; conservation; food/economics	7	2010	2021
food/agriculture	density/count	viticulture cultivars percent	present		invertebrates	food/economics	1	2013	2013

food/agriculture	density/count	waste seeds rice density	present	06; 12	birds	conservation	1	2018	2018
food/agriculture	density/count	waste seeds soybean density	present	06; 12	birds	conservation	1	2018	2018
food/agriculture	density/count	woody cultivations percent	past; present; future		birds	disturbance/habitat change	1	2010	2010
food/agriculture	descriptive	agricultural and anthropic areas	present		trees/shrubs	invasions	1	2017	2017
food/agriculture	descriptive	agricultural areas	past; present		mammals; birds; invertebrates; reptiles; fish; trees/shrubs; herbaceous plants	disturbance/habitat change; reintroduction/restoration; exploratory; conservation; conflict/collisions; invasions; food/economics	30	2003	2021
food/agriculture	descriptive	agricultural areas 10m radius	present		invertebrates	exploratory	1	2020	2020
food/agriculture	descriptive	agricultural areas 500m radius	present		birds	food/economics	1	2020	2020
food/agriculture	descriptive	agricultural areas and forest edges area size	present	15	mammals	conservation	1	2019	2019
food/agriculture	descriptive	agricultural areas and natural vegetation mosaic	present		birds	conservation	1	2020	2020
food/agriculture	descriptive	agricultural areas bare soil edges length	present	15	birds	conservation	1	2016	2016
food/agriculture	descriptive	agricultural areas edge	present		invertebrates	exploratory	1	2020	2020
food/agriculture	descriptive	agricultural areas extensive	present		mammals	conservation	1	2009	2009
food/agriculture	descriptive	agricultural areas grass edges length	present		birds	conservation	1	2016	2016
food/agriculture	descriptive	agricultural areas heterogeneous	present		mammals; herbaceous plants; birds	conservation; invasions; conflict/collisions; exploratory	5	2009	2019

food/agriculture	descriptive	agricultural areas heterogeneous area size	present		herbaceous plants	exploratory	1	2020	2020
food/agriculture	descriptive	agricultural areas heterogeneous percent	present; future		invertebrates	disturbance/habitat change; exploratory	2	2015	2021
food/agriculture	descriptive	agricultural areas intensive	present		mammals	conservation	2	2009	2009
food/agriculture	descriptive	agricultural areas intensive percent 10km radius	present		mammals	conservation	1	2013	2013
food/agriculture	descriptive	agricultural areas intensive percent 1km radius	present		mammals	conservation	1	2013	2013
food/agriculture	descriptive	agricultural areas intensive percent 4km radius	present		mammals	conservation	1	2013	2013
food/agriculture	descriptive	agricultural areas intensive percent 7km radius	present		mammals	conservation	1	2013	2013
food/agriculture	descriptive	agricultural areas mixed	present		birds	conservation	1	2017	2017
food/agriculture	descriptive	agricultural areas mosaic	present		birds	exploratory	1	2009	2009
food/agriculture	descriptive	agricultural areas natural	present		mammals	conservation	1	2016	2016
food/agriculture	descriptive	agricultural areas patch	present		invertebrates	exploratory	1	2020	2020
food/agriculture	descriptive	agricultural areas riparian 100m radius	present		invertebrates	exploratory	1	2020	2020
food/agriculture	descriptive	agricultural areas riparian edges length	present		birds	conservation	1	2016	2016
food/agriculture	descriptive	agricultural areas shrub edges length	present		birds	conservation	1	2016	2016
food/agriculture	descriptive	agricultural areas tree edges length	present	15	birds	conservation	1	2016	2016
food/agriculture	descriptive	agricultural areas use type	present		invertebrates	food/economics	1	2019	2019
food/agriculture	descriptive	agricultural areas woody	present		birds; mammals	exploratory; conservation	2	2009	2012
food/agriculture	descriptive	agricultural grasslands	present		mammals	reintroduction/restoration	1	2017	2017
food/agriculture	descriptive	agricultural meadows	present		mammals	exploratory	2	2009	2020
food/agriculture	descriptive	agroforestry areas gyrate	present	15	mammals	conservation	1	2016	2016
food/agriculture	descriptive	agroforestry without natural vegetation	present	15	mammals	reintroduction/restoration	1	2020	2020
food/agriculture	descriptive	aquaculture finfish	present	14	herbaceous plants	exploratory	1	2020	2020

food/agriculture	descriptive	aquaculture sites	present	14	invertebrates	conservation	1	2014	2014
food/agriculture	descriptive	arable and farming lands	past; present		birds	disturbance/habitat change	1	2017	2017
food/agriculture	descriptive	arable land	present; future	15	birds; mammals; invertebrates	exploratory; conservation; reintroduction/restoration; food/economics; disturbance/habitat change	14	2000	2021
food/agriculture	descriptive	arable land and meadows residual percent	present	15	invertebrates	disturbance/habitat change	1	2009	2009
food/agriculture	descriptive	arable land non-irrigated	present	15	invertebrates; birds	disturbance/habitat change	2	2014	2018
food/agriculture	descriptive	arable land unclassified percent	present; future	15	birds	food/economics	1	2014	2014
food/agriculture	descriptive	arable land with trees	present	15	birds	exploratory	1	2012	2012
food/agriculture	descriptive	complex cultivation patterns	present		birds; invertebrates	conservation; disturbance/habitat change	2	2018	2020
food/agriculture	descriptive	crop cereal	present		birds	conservation; exploratory	2	2010	2013
food/agriculture	descriptive	crop dominance class	present	1	birds	food/economics	1	2021	2021
food/agriculture	descriptive	crop dry	present		birds	disturbance/habitat change	1	2021	2021
food/agriculture	descriptive	crop dry tree	present	15	reptiles	exploratory	1	2007	2007
food/agriculture	descriptive	crop maize presence	present		birds; mammals	food/economics; conflict/collisions	2	2013	2021
food/agriculture	descriptive	crop oilseed rape	present		birds	conservation	1	2012	2012
food/agriculture	descriptive	crop soybean presence	present		birds	food/economics	1	2021	2021
food/agriculture	descriptive	crop summer cereals	present		birds	conservation	1	2012	2012
food/agriculture	descriptive	crop type	present		birds; invertebrates	conservation; exploratory	2	2013	2017
food/agriculture	descriptive	crop winter cereals	present		birds	conservation	1	2012	2012
food/agriculture	descriptive	crop winter grain percent	present; future		birds	food/economics; exploratory	2	2011	2014

food/agriculture	descriptive	crop winter wheat crops percent	present	2	birds	exploratory	1	2018	2018
food/agriculture	descriptive	cropland	past; present; future		herbaceous plants; invertebrates; mammals; birds; microorganisms; trees/shrubs; reptiles	human health/safety; exploratory; conservation; disturbance/habitat change; food/economics; reintroduction/restoration	24	2004	2021
food/agriculture	descriptive	cropland >50 percent and vegetation mosaic	present		mammals	exploratory	1	2020	2020
food/agriculture	descriptive	cropland and <50 percent vegetation mosaic	present		mammals	exploratory	1	2020	2020
food/agriculture	descriptive	cropland and grassland percent	present		mammals	conservation	1	2015	2015
food/agriculture	descriptive	cropland and natural mosaic	present; future		invertebrates	disturbance/habitat change	1	2014	2014
food/agriculture	descriptive	cropland and natural vegetation mosaic	present; future		mammals	disturbance/habitat change; exploratory	2	2019	2021
food/agriculture	descriptive	cropland and pastures	present		mammals	exploratory	1	2015	2015
food/agriculture	descriptive	cropland edges length sum	present		birds	conservation	1	2014	2014
food/agriculture	descriptive	cropland historical yr1900	past		herbaceous plants	exploratory	1	2016	2016
food/agriculture	descriptive	cropland mixed vegetation	present		mammals	reintroduction/restoration	1	2013	2013
food/agriculture	descriptive	cropland or natural mosaic	present		birds	exploratory	1	2010	2010
food/agriculture	descriptive	cropland rainfed	present		mammals; birds	exploratory; conservation	2	2011	2013
food/agriculture	descriptive	cropland seeded	present		birds	conservation	1	2018	2018
food/agriculture	descriptive	cropland type	past		mammals	conflict/collisions	1	2019	2019
food/agriculture	descriptive	cropland vegetation mosaic	present		mammals	human health/safety	1	2018	2018
food/agriculture	descriptive	crops dry herbaceous present absent	present	2	amphibians; birds; mammals; reptiles	exploratory	2	2007	2016

food/agriculture	descriptive	crops dry herbaceous present absent percent	present	2	birds; mammals	food/economics	1	2007	2007
food/agriculture	descriptive	crops fruit tree	present	15	amphibians; birds; mammals; reptiles	exploratory	1	2016	2016
food/agriculture	descriptive	crops nitrogen-fixing	past	2	mammals	exploratory	1	2021	2021
food/agriculture	descriptive	crops perennial	past	2	mammals	exploratory	1	2021	2021
food/agriculture	descriptive	cultivated areas	past; present		mammals; amphibians; fish; herbaceous plants; invertebrates; microorganisms; birds; reptiles; trees/shrubs	exploratory; invasions; conservation	5	2014	2016
food/agriculture	descriptive	cultivated areas heterogeneous and patchy	present		mammals	conservation	1	2004	2004
food/agriculture	descriptive	cultivated areas mixed	present		birds	conservation	1	2013	2013
food/agriculture	descriptive	cultivated crops	present	2	birds	exploratory	1	2016	2016
food/agriculture	descriptive	cultivations and plantations	present		herbaceous plants	invasions	1	2018	2018
food/agriculture	descriptive	dredge fishing	present	14	herbaceous plants	exploratory	1	2020	2020
food/agriculture	descriptive	exotic trees for feeding	present		birds	invasions	1	2018	2018
food/agriculture	descriptive	fallow land	present		invertebrates; birds	food/economics; conservation; exploratory	3	2010	2016
food/agriculture	descriptive	fallow land 25m radius	present		invertebrates	food/economics	1	2013	2013
food/agriculture	descriptive	fallow land 50m radius	present		invertebrates	food/economics	1	2013	2013
food/agriculture	descriptive	family horticulture	present		birds	exploratory	1	2012	2012
food/agriculture	descriptive	FAO ruminant production systems	present		birds	conservation	1	2017	2017
food/agriculture	descriptive	farmlands	present		mammals	conservation	1	2020	2020

food/agriculture	descriptive	fertilizer type	present	2	invertebrates	exploratory	1	2013	2013
food/agriculture	descriptive	fishing vessel distribution cold store	present	14	birds	conservation	1	2014	2014
food/agriculture	descriptive	fishing vessel distribution freezer	present	14	birds	conservation	1	2014	2014
food/agriculture	descriptive	food source type human-related	present		birds	conservation	1	2017	2017
food/agriculture	descriptive	forest harvested	present	15	mammals	disturbance/habitat change	1	2018	2018
food/agriculture	descriptive	grazing areas	present; future		herbaceous plants; invertebrates; mammals	disturbance/habitat change; exploratory; invasions	5	2009	2021
food/agriculture	descriptive	grazing areas absence	present		herbaceous plants	invasions	1	2019	2019
food/agriculture	descriptive	grazing areas edge	present		birds	conflict/collisions	1	2015	2015
food/agriculture	descriptive	grazing edges length sum	present		birds	conservation	1	2014	2014
food/agriculture	descriptive	grazing intensity class	present	1	birds	exploratory	1	2017	2017
food/agriculture	descriptive	grazing modified	present		birds	disturbance/habitat change	1	2021	2021
food/agriculture	descriptive	grazing natural	present		birds	disturbance/habitat change	1	2021	2021
food/agriculture	descriptive	grazing nongrazing areas	present		mammals	exploratory	1	2014	2014
food/agriculture	descriptive	harvesting presence	present		herbaceous plants	invasions	1	2015	2015
food/agriculture	descriptive	heterogenous agricultural areas	present		mammals	exploratory	1	2015	2015
food/agriculture	descriptive	horse	present		mammals	disturbance/habitat change	1	2017	2017
food/agriculture	descriptive	horticulture	present		birds	exploratory	1	2012	2012
food/agriculture	descriptive	horticulture 10m radius	present		invertebrates	exploratory	1	2020	2020
food/agriculture	descriptive	horticulture edge	present		invertebrates	exploratory	1	2020	2020
food/agriculture	descriptive	irrigated agricultural areas	present		mammals; birds	exploratory; conservation	3	2011	2013

food/agriculture	descriptive	irrigated areas	present		birds	disturbance/habitat change	1	2021	2021
food/agriculture	descriptive	irrigated crops	present	2	birds	exploratory	1	2009	2009
food/agriculture	descriptive	irrigated fruit trees	present; future		birds	exploratory	1	2009	2009
food/agriculture	descriptive	irrigated herbaceous crops	present; future	2	amphibians; birds; mammals; reptiles	exploratory	3	2007	2016
food/agriculture	descriptive	irrigated tree crops	present	02; 15	reptiles	exploratory	1	2007	2007
food/agriculture	descriptive	irrigation system presence	present	6	birds	conservation	1	2020	2020
food/agriculture	descriptive	last tillage type	present		invertebrates	exploratory	1	2013	2013
food/agriculture	descriptive	livestock cattle presence	present		mammals	disturbance/habitat change	2	2015	2017
food/agriculture	descriptive	livestock exploitation presence	present		birds	exploratory	1	2005	2005
food/agriculture	descriptive	livestock free-grazing	present		mammals	conflict/collisions	1	2020	2020
food/agriculture	descriptive	livestock use	present		birds	conservation	1	2021	2021
food/agriculture	descriptive	meadow unfertilized	present		herbaceous plants	exploratory	1	2009	2009
food/agriculture	descriptive	mercury fish advisory	present		birds	conservation	1	2017	2017
food/agriculture	descriptive	mixed cultivation patterns	present		birds	disturbance/habitat change	1	2014	2014
food/agriculture	descriptive	nitrogen fertilizer	present	2	amphibians	exploratory	1	2021	2021
food/agriculture	descriptive	non-irrigated arable land	present	15	invertebrates; birds	food/economics; conservation	2	2020	2021
food/agriculture	descriptive	non-irrigated crops	present	2	birds	exploratory	1	2009	2009
food/agriculture	descriptive	non-irrigated fruit trees	present; future		birds	exploratory	1	2009	2009
food/agriculture	descriptive	non-irrigated herbaceous crops	present; future	2	birds	exploratory	1	2009	2009
food/agriculture	descriptive	non-irrigated vineyards	present; future		birds	exploratory	1	2009	2009

food/agriculture	descriptive	open plantations coffee percent	present		amphibians	conservation	1	2015	2015
food/agriculture	descriptive	orchards	present		mammals; birds	conservation; exploratory	3	2012	2021
food/agriculture	descriptive	orchards fruit and olives	present		mammals; birds	exploratory	2	2004	2021
food/agriculture	descriptive	orchards fruit tree	present	15	birds	conservation	1	2020	2020
food/agriculture	descriptive	orchards fruit tree and berries	present	15	invertebrates	food/economics	1	2018	2018
food/agriculture	descriptive	orchards olives	present		amphibians; birds; mammals; reptiles	exploratory	3	2009	2016
food/agriculture	descriptive	overgrazing coefficient	present	2	trees/shrubs	disturbance/habitat change	1	2019	2019
food/agriculture	descriptive	pastoral areas residual	present		mammals	conservation	1	2014	2014
food/agriculture	descriptive	pastures	present		invertebrates; reptiles; birds; mammals; microorganisms; amphibians; herbaceous plants	food/economics; invasions; exploratory; conservation; disturbance/habitat change	16	2009	2021
food/agriculture	descriptive	pastures and forage grasslands	present		birds	conservation	1	2018	2018
food/agriculture	descriptive	pastures and non-tilled grasses	present		reptiles	disturbance/habitat change	1	2006	2006
food/agriculture	descriptive	pastures gyrate	present		mammals	conservation	1	2016	2016
food/agriculture	descriptive	pastures high mountain	present		herbaceous plants	exploratory	1	2009	2009
food/agriculture	descriptive	pastures historical yr1900	past		herbaceous plants	exploratory	1	2016	2016
food/agriculture	descriptive	pastures improved	present; future		birds; mammals	conservation; exploratory	2	2017	2017
food/agriculture	descriptive	pastures mean cover class	present	1	mammals	conservation	1	2006	2006

food/agriculture	descriptive	pastures natural	present		mammals	reintroduction/restoration; conservation	2	2009	2020
food/agriculture	descriptive	pastures type	present		birds; mammals	disturbance/habitat change	1	2010	2010
food/agriculture	descriptive	pastures unfertilized	present		herbaceous plants	exploratory	1	2009	2009
food/agriculture	descriptive	pastures unimproved	present; future		birds	conservation	1	2017	2017
food/agriculture	descriptive	permanent crop land use upstream	present	15	fish	conservation	1	2018	2018
food/agriculture	descriptive	permanent crops	present; future	2	herbaceous plants; invertebrates	invasions; disturbance/habitat change	2	2013	2017
food/agriculture	descriptive	permanent crops and pastures	present	2	birds	conservation	1	2017	2017
food/agriculture	descriptive	permanent cultures	present		birds	exploratory	1	2018	2018
food/agriculture	descriptive	phosphorus fertilizer	present	2	amphibians	exploratory	1	2021	2021
food/agriculture	descriptive	plantation	present		mammals	exploratory	1	2015	2015
food/agriculture	descriptive	plantation and vegetation matrix	present		mammals	conservation	1	2003	2003
food/agriculture	descriptive	plantation non-native conifer forest	present	15	mammals	reintroduction/restoration	1	2017	2017
food/agriculture	descriptive	plantations acacia percent	present		trees/shrubs	invasions	1	2020	2020
food/agriculture	descriptive	plantations adult poplar percent	present		mammals	disturbance/habitat change	1	2015	2015
food/agriculture	descriptive	plantations adult willow percent	present		mammals	disturbance/habitat change	1	2015	2015
food/agriculture	descriptive	plantations coconut percent	present		amphibians	conservation	1	2015	2015
food/agriculture	descriptive	plantations coffee percent	present		amphibians	conservation	1	2015	2015
food/agriculture	descriptive	plantations coniferous percent	present		invertebrates	conservation	1	2011	2011
food/agriculture	descriptive	plantations eucalyptus frequency	present		mammals	conservation	1	2020	2020

food/agriculture	descriptive	plantations eucalyptus percent	past; present; future		mammals; birds	disturbance/habitat change; exploratory	2	2004	2014
food/agriculture	descriptive	plantations fruit	present		invertebrates	disturbance/habitat change	1	2018	2018
food/agriculture	descriptive	plantations fruit and berry percent	present		birds	exploratory; reintroduction/restoration	2	2012	2013
food/agriculture	descriptive	plantations old deciduous conifer percent	present		mammals	disturbance/habitat change	1	2014	2014
food/agriculture	descriptive	plantations old evergreen conifer percent	present		mammals	disturbance/habitat change	1	2014	2014
food/agriculture	descriptive	plantations percent	present		birds; mammals; microorganisms; reptiles	exploratory; conservation	3	2011	2016
food/agriculture	descriptive	plantations pine	present		birds	conservation	1	2013	2013
food/agriculture	descriptive	plantations pine and eucalyptus percent	present		mammals	disturbance/habitat change	1	2019	2019
food/agriculture	descriptive	plantations poplar	present		birds	exploratory	1	2012	2012
food/agriculture	descriptive	plantations woody	present		birds	exploratory	1	2012	2012
food/agriculture	descriptive	plantations young evergreen conifer percent	present		mammals	disturbance/habitat change	1	2014	2014
food/agriculture	descriptive	plantations young poplar percent	present		mammals	disturbance/habitat change	1	2015	2015
food/agriculture	descriptive	plantations young willow percent	present		mammals	disturbance/habitat change	1	2015	2015
food/agriculture	descriptive	ploughing class	present	1	invertebrates	exploratory	1	2013	2013
food/agriculture	descriptive	rice fields	present		birds; invertebrates	exploratory; human health/safety	2	2010	2012
food/agriculture	descriptive	rice paddy	present		birds; reptiles; fish; invertebrates	reintroduction/restoration; conflict/collisions; human health/safety; exploratory	5	2012	2021

food/agriculture	descriptive	row crop fields	present		reptiles	disturbance/habitat change	1	2006	2006
food/agriculture	descriptive	row crops	present	2	mammals	exploratory	1	2017	2017
food/agriculture	descriptive	sea fisheries areas	present	14	invertebrates	conservation	1	2014	2014
food/agriculture	descriptive	shellfish aquaculture	present	14	herbaceous plants	exploratory	1	2020	2020
food/agriculture	descriptive	tree plantations	present	15	mammals	exploratory	2	2017	2020
food/agriculture	descriptive	tree plantations percent	present	15	birds	conservation	1	2003	2003
food/agriculture	descriptive	uncultivated land	present		birds	disturbance/habitat change	1	2012	2012
food/agriculture	descriptive	vineyards	present		birds; mammals; invertebrates; amphibians; reptiles	exploratory; conservation; disturbance/habitat change; invasions	10	2009	2021
food/agriculture	descriptive	vineyards 25m radius	present		invertebrates	food/economics	1	2013	2013
food/agriculture	descriptive	vineyards 50m radius	present		invertebrates	food/economics	1	2013	2013
food/agriculture	descriptive	vineyards and olive tree orchards	present	15	mammals	conservation	1	2004	2004
food/agriculture	descriptive	viticulture	present		invertebrates	food/economics	1	2013	2013
food/agriculture	descriptive	viticulture traction or terracing	present		invertebrates	food/economics	1	2013	2013
food/agriculture	distance	abandoned agricultural areas new distance	present		mammals	exploratory	1	2015	2015
food/agriculture	distance	abandoned agricultural areas old distance	present		mammals	exploratory	1	2015	2015
food/agriculture	distance	abandoned plantation distance	present		herbaceous plants	invasions	1	2016	2016
food/agriculture	distance	agricultural areas adjacency	present		herbaceous plants	invasions	1	2010	2010
food/agriculture	distance	agricultural areas adjacency watershed	present		herbaceous plants	invasions	1	2010	2010
food/agriculture	distance	agricultural areas adjacency watershed mean	present		herbaceous plants	invasions	1	2010	2010

food/agriculture	distance	agricultural areas and orchards distance	present		birds	disturbance/habitat change	1	2018	2018
food/agriculture	distance	agricultural areas distance	past; present		mammals; birds; herbaceous plants; invertebrates; trees/shrubs; amphibians; reptiles	disturbance/habitat change; conservation; reintroduction/restoration; exploratory; invasions; conflict/collisions; food/economics; human health/safety	37	2004	2021
food/agriculture	distance	agricultural areas distance 100m radius	present		amphibians	disturbance/habitat change	1	2009	2009
food/agriculture	distance	agricultural areas distance 1km radius	present		amphibians	disturbance/habitat change	1	2009	2009
food/agriculture	distance	agricultural areas distance 2km radius	present		amphibians	disturbance/habitat change	1	2009	2009
food/agriculture	distance	agricultural areas distance 30m radius	present		amphibians	disturbance/habitat change	1	2009	2009
food/agriculture	distance	agricultural areas distance 500m radius	present		amphibians	disturbance/habitat change	1	2009	2009
food/agriculture	distance	agricultural areas extensive distance	past; present		birds	conservation	1	2011	2011
food/agriculture	distance	anthropic meadows and thickets distance	present		mammals	invasions	1	2021	2021
food/agriculture	distance	apple orchards and oat fields distance	present		mammals	exploratory	2	2019	2020
food/agriculture	distance	aquaculture distance	present	14	invertebrates; reptiles	invasions; conflict/collisions	2	2021	2021
food/agriculture	distance	aquaculture salmon distance	present	14	mammals	conservation	2	2016	2019
food/agriculture	distance	arable horticulture distance	present		mammals	reintroduction/restoration	1	2021	2021

food/agriculture	distance	arable land distance	present; future	15	herbaceous plants; mammals; amphibians; birds	invasions; conflict/collisions; exploratory; conservation	8	2009	2021
food/agriculture	distance	arable land non-irrigated distance	present	15	birds	disturbance/habitat change	1	2020	2020
food/agriculture	distance	artificial tree plantations distance	present	15	mammals	disturbance/habitat change	1	2019	2019
food/agriculture	distance	complex cultivation patterns distance	present		mammals	disturbance/habitat change	1	2014	2014
food/agriculture	distance	crcropland vegetation mosaic distance	present		mammals	conservation; disturbance/habitat change; exploratory	3	2015	2021
food/agriculture	distance	crop and native mosaic distance	present		mammals	conservation	1	2021	2021
food/agriculture	distance	cropland adjacency	present		birds	disturbance/habitat change	1	2018	2018
food/agriculture	distance	cropland and vegetation mosaic distance	present		reptiles	disturbance/habitat change	1	2015	2015
food/agriculture	distance	cropland distance	present; future		mammals; reptiles; amphibians; invertebrates; birds	conservation; exploratory; disturbance/habitat change; human health/safety; food/economics	17	2010	2021
food/agriculture	distance	cropland extent	present		birds	conservation	1	2021	2021
food/agriculture	distance	cropland rainfed distance	present		reptiles	disturbance/habitat change	1	2015	2015
food/agriculture	distance	cultivated areas distance	present		birds; herbaceous plants	conservation; invasions	3	2010	2018
food/agriculture	distance	cultivated crops distance	present	2	mammals	exploratory	1	2018	2018
food/agriculture	distance	farm pond distance	present		fish	conservation	1	2019	2019

food/agriculture	distance	farm roads distance	present		birds	disturbance/habitat change	1	2016	2016
food/agriculture	distance	farmlands distance	present		birds; mammals; amphibians; fish; invertebrates; reptiles; microorganisms	conservation; exploratory; disturbance/habitat change; invasions; food/economics; reintroduction/restoration	15	2012	2021
food/agriculture	distance	farmlands dry distance	present		birds; mammals	exploratory; conservation; invasions	3	2017	2021
food/agriculture	distance	fishing activity distance	present	14	mammals	conservation	1	2016	2016
food/agriculture	distance	fishing distance	present	14	invertebrates	human health/safety	1	2018	2018
food/agriculture	distance	fishing haul depth	present	14	fish	disturbance/habitat change	1	2015	2015
food/agriculture	distance	fishing ports sailing distance	present	14	microorganisms	invasions	1	2021	2021
food/agriculture	distance	grazing adjacency	present		birds	disturbance/habitat change	1	2018	2018
food/agriculture	distance	grazing distance	present		invertebrates	human health/safety	1	2018	2018
food/agriculture	distance	harvested forest hardwood poletimber distance	present	15	birds	exploratory	1	2000	2000
food/agriculture	distance	harvested forest hardwood regeneration distance	present	15	birds	exploratory	1	2000	2000
food/agriculture	distance	harvested forest hardwood sawtimber distance	present	15	birds	exploratory	1	2000	2000
food/agriculture	distance	harvested forest mixed poletimber burned >3yrs distance	present	15	birds	exploratory	1	2000	2000
food/agriculture	distance	harvested forest mixed regeneration distance	present	15	birds	exploratory	1	2000	2000
food/agriculture	distance	harvested forest mixed sawtimber burned <3yrs distance	present	15	birds	exploratory	1	2000	2000

food/agriculture	distance	harvested forest mixed sawtimber burned >3yrs distance	present	15	birds	exploratory	1	2000	2000
food/agriculture	distance	harvested forest pine poletimber burned >3yrs distance	present	15	birds	exploratory	1	2000	2000
food/agriculture	distance	harvested forest pine regeneration distance	present	15	birds	exploratory	1	2000	2000
food/agriculture	distance	harvested forest pine sawtimber burned <3yrs distance	present	15	birds	exploratory	1	2000	2000
food/agriculture	distance	harvested forest pine sawtimber burned >3yrs distance	present	15	birds	exploratory	1	2000	2000
food/agriculture	distance	irrigated areas distance	present		invertebrates	human health/safety	1	2010	2010
food/agriculture	distance	irrigated farmlands distance	present		birds; mammals	exploratory; conservation; invasions	4	2015	2021
food/agriculture	distance	irrigated land permanent distance	present		birds	disturbance/habitat change	1	2020	2020
food/agriculture	distance	irrigation canal distance	present	6	birds	conservation	1	2021	2021
food/agriculture	distance	livestock cattle farm distance	present		birds	exploratory	1	2007	2007
food/agriculture	distance	livestock cattle pastures distance	present		birds	conservation	1	2016	2016
food/agriculture	distance	livestock depredation event cost distance	present		mammals	conflict/collisions	1	2017	2017
food/agriculture	distance	livestock grazing distance	present		mammals	disturbance/habitat change	1	2019	2019
food/agriculture	distance	livestock sheep farm distance	present		mammals	conflict/collisions	1	2017	2017
food/agriculture	distance	orchards distance	present		amphibians; mammals; birds	conservation; invasions	3	2009	2018
food/agriculture	distance	orchards olives distance	present		mammals	disturbance/habitat change	1	2014	2014

food/agriculture	distance	palm oil plantation distance	present; future		invertebrates	disturbance/habitat change	1	2018	2018
food/agriculture	distance	pastoral land distance	present		birds	exploratory	1	2020	2020
food/agriculture	distance	pastures and crops distance	present	2	herbaceous plants	invasions	1	2020	2020
food/agriculture	distance	pastures cropland distance	present		herbaceous plants; trees/shrubs	invasions	1	2021	2021
food/agriculture	distance	pastures distance	present; future		mammals; birds; amphibians; reptiles; herbaceous plants	conflict/collisions; disturbance/habitat change; conservation; exploratory	11	2012	2020
food/agriculture	distance	perennial crops distance	present	2	mammals	exploratory	1	2015	2015
food/agriculture	distance	plantation forests distance	present	15	birds	disturbance/habitat change	1	2018	2018
food/agriculture	distance	plantations distance	present		mammals	invasions	1	2021	2021
food/agriculture	distance	rangeland distance	present		mammals; birds	conservation; disturbance/habitat change; invasions	4	2017	2021
food/agriculture	distance	rangeland good distance	present		reptiles	conservation	1	2021	2021
food/agriculture	distance	rangeland poor distance	present	1	reptiles	conservation	1	2021	2021
food/agriculture	distance	rice paddy distance	present		mammals; reptiles	conservation; exploratory	4	2010	2021
food/agriculture	distance	roads farmlands distance	present		birds	conservation	1	2017	2017
food/agriculture	distance	shellfish farm distance	present		mammals	conservation	1	2019	2019
food/agriculture	distance	tea plantations distance	present		mammals	conservation	1	2015	2015
food/agriculture	distance	timber harvest area distance	present		amphibians	exploratory	1	2011	2011
food/agriculture	distance	tree plantation distance	present	15	mammals; invertebrates	disturbance/habitat change; human health/safety	2	2018	2019
food/agriculture	distance	winery distance	present		invertebrates	food/economics	1	2019	2019

food/agriculture	index	agricultural areas aggregation index	present		mammals	exploratory	1	2014	2014
food/agriculture	index	agricultural areas clumpiness	present		mammals	invasions	1	2016	2016
food/agriculture	index	agricultural areas cohesion	present		mammals	exploratory	1	2014	2014
food/agriculture	index	agricultural areas low-intensity	present		birds	exploratory	2	2007	2007
food/agriculture	index	agricultural modification	present		reptiles	exploratory	1	2018	2018
food/agriculture	index	crop patch cohesion index	present		mammals	reintroduction/restoration	1	2021	2021
food/agriculture	index	cropland index	present		amphibians	disturbance/habitat change	1	2020	2020
food/agriculture	index	fertilizer input index	present	2	microorganisms	conservation	1	2020	2020
food/agriculture	index	fishing activity index	present	14	birds	exploratory	1	2014	2014
food/agriculture	index	fishing effort	present	14	invertebrates; mammals	exploratory	2	2015	2020
food/agriculture	index	fishing effort boat	present	14	fish	conservation	1	2018	2018
food/agriculture	index	fishing effort shore	present	14	fish	conservation	1	2018	2018
food/agriculture	index	grazing capacity	present		mammals	conservation	1	2013	2013
food/agriculture	index	grazing intensity	present		invertebrates; trees/shrubs; birds	exploratory; disturbance/habitat change; conservation	4	2000	2020
food/agriculture	index	grazing intensity index	present		birds	conservation	1	2000	2000
food/agriculture	index	harvest treatment index	present		mammals	disturbance/habitat change	1	2018	2018
food/agriculture	index	harvest wild yams intensity	present		herbaceous plants	food/economics	1	2015	2015
food/agriculture	index	human footprint agricultural	present		mammals	exploratory	1	2014	2014
food/agriculture	index	livestock encounter rate	present		mammals	conservation	1	2013	2013
food/agriculture	index	livestock large use probability	present		mammals	conflict/collisions	1	2013	2013
food/agriculture	index	meadow middle intensity use	present		herbaceous plants	exploratory	1	2009	2009
food/agriculture	index	pastures change yr1900-2005	present		herbaceous plants	exploratory	1	2016	2016

food/agriculture	index	pastures high productivity	present; future		birds	exploratory	1	2021	2021
food/agriculture	index	pastures low productivity	present; future		birds	exploratory	1	2021	2021
food/agriculture	index	ship fishing sales value	present	14	microorganisms	invasions	1	2021	2021
food/agriculture	index	small livestock use probability	present		mammals	conflict/collisions	1	2013	2013
food/agriculture	index	vineyards index	present		amphibians	disturbance/habitat change	1	2020	2020
food/agriculture	index	wet pastures ratio	past		birds	disturbance/habitat change	1	2009	2009
food/agriculture	size	agricultural areas area-weighted mean	present		mammals	conservation; exploratory	2	2014	2021
food/agriculture	size	agricultural areas area size	past; present		mammals; herbaceous plants; birds	exploratory; food/economics; invasions	6	2011	2021
food/agriculture	size	agricultural areas mean size	present		fish; invertebrates	conservation	1	2011	2011
food/agriculture	size	agricultural areas mean size 1000m radius	present		mammals	exploratory	1	2017	2017
food/agriculture	size	agricultural areas mean size 100m riparian radius	present		fish	exploratory	1	2009	2009
food/agriculture	size	agricultural areas mean size 100m upstream radius	present		fish	exploratory	1	2009	2009
food/agriculture	size	agricultural areas mean size 2000m radius	present		mammals	exploratory	1	2017	2017
food/agriculture	size	agricultural areas mean size 250m radius	present		mammals	exploratory	1	2017	2017
food/agriculture	size	agricultural areas mean size 500m radius	present		mammals	exploratory	1	2017	2017
food/agriculture	size	agricultural areas mean size subcatchment radius	present		fish	exploratory	1	2009	2009
food/agriculture	size	agricultural areas other size	present		invertebrates	exploratory	1	2017	2017

food/agriculture	size	agricultural areas patch size mean	present		invertebrates	exploratory	1	2009	2009
food/agriculture	size	agricultural areas size	present		mammals	invasions	1	2020	2020
food/agriculture	size	agricultural areas with irrigated areas ha	present		herbaceous plants	food/economics	1	2018	2018
food/agriculture	size	agricultural other area size	past; present		invertebrates	disturbance/habitat change	1	2020	2020
food/agriculture	size	agricultural upstream area	present		invertebrates	disturbance/habitat change	1	2015	2015
food/agriculture	size	agroforestry areas area-weighted mean	present	15	mammals	conservation	1	2016	2016
food/agriculture	size	arable horticulture area size	present		mammals	invasions	1	2010	2010
food/agriculture	size	arable land area size	present	15	herbaceous plants; microorganisms	exploratory; conservation	2	2020	2020
food/agriculture	size	arable land area size 1500m radius	present	15	birds	conservation	1	2020	2020
food/agriculture	size	arable land with trees size	present	15	birds	exploratory	1	2012	2012
food/agriculture	size	cassava harvested area size	present		invertebrates	food/economics	1	2011	2011
food/agriculture	size	crop damage beans area size	present		mammals	food/economics	1	2000	2000
food/agriculture	size	crop damage coffee area size	present		mammals	food/economics	1	2000	2000
food/agriculture	size	crop damage corn area size	present		mammals	food/economics	1	2000	2000
food/agriculture	size	crop damage grasses area size	present		mammals	food/economics	1	2000	2000
food/agriculture	size	crop damage oats area size	present		mammals	food/economics	1	2000	2000
food/agriculture	size	crop damage rice area size	present		mammals	food/economics	1	2000	2000
food/agriculture	size	crop damage sorghum area size	present		mammals	food/economics	1	2000	2000
food/agriculture	size	crop damage sugarcane area size	present		mammals	food/economics	1	2000	2000
food/agriculture	size	crop damage wheat area size	present		mammals	food/economics	1	2000	2000

food/agriculture	size	crop height April	present		birds	conservation	1	2017	2017
food/agriculture	size	crop height May	present		birds	conservation	1	2017	2017
food/agriculture	size	crop shaded coffee area size	present		mammals	exploratory	1	2011	2011
food/agriculture	size	cropland area-weighted mean	present		mammals	conservation	1	2016	2016
food/agriculture	size	cropland area change yr1900-2005	present		herbaceous plants	exploratory	1	2016	2016
food/agriculture	size	cropland edge length	present		birds	disturbance/habitat change; conflict/collisions	2	2015	2018
food/agriculture	size	cropland size	past; present		mammals; birds	exploratory; disturbance/habitat change; conflict/collisions	3	2015	2021
food/agriculture	size	cultivated areas size	present		invertebrates; birds	human health/safety; conservation	2	2020	2021
food/agriculture	size	farmlands area size	present		birds; invertebrates	conservation; disturbance/habitat change; invasions	3	2015	2021
food/agriculture	size	farmlands area size km	present		birds	conservation	1	2017	2017
food/agriculture	size	grapevine cultivar size	present		invertebrates	food/economics	1	2015	2015
food/agriculture	size	grazing areas size	present		birds	disturbance/habitat change; conflict/collisions	2	2015	2018
food/agriculture	size	grazing edge length	present		birds	disturbance/habitat change	1	2018	2018
food/agriculture	size	harvested area	present		mammals	exploratory	1	2015	2015
food/agriculture	size	meadows and orchards area size	present		herbaceous plants	invasions	1	2013	2013
food/agriculture	size	non-irrigated arable land size	present	15	birds	exploratory	1	2012	2012
food/agriculture	size	orchards tree DBH	present	15	birds	conservation	1	2020	2020
food/agriculture	size	orchards tree height	present	15	birds	conservation	1	2020	2020
food/agriculture	size	pastures and meadows area size	present		birds	conservation	1	2020	2020
food/agriculture	size	pastures and permanent grassland size	present		birds	exploratory	1	2012	2012

food/agriculture	size	pastures area-weighted mean	present		mammals	conservation	1	2016	2016
food/agriculture	size	pastures area size	present		mammals	conflict/collisions; exploratory	2	2011	2015
food/agriculture	size	pastures intensive area	present		birds	conservation	1	2007	2007
food/agriculture	size	pastures patch mean size	present		fish; invertebrates	conservation; exploratory	2	2009	2011
food/agriculture	size	pastures patch mean size 100m upstream radius	present		fish	exploratory	1	2009	2009
food/agriculture	size	pastures patch mean size subcatchment radius	present		fish	exploratory	1	2009	2009
food/agriculture	size	pastures patch size mean	present		invertebrates	exploratory	1	2009	2009
food/agriculture	size	pastures size	present		herbaceous plants; mammals	exploratory; invasions	2	2010	2020
food/agriculture	size	pastures size 1500m radius	present		birds	conservation	1	2020	2020
food/agriculture	size	pastures size mean	present		mammals	conservation	1	2003	2003
food/agriculture	size	reindeer husbandry area	present		mammals	reintroduction/restoration	1	2018	2018
food/agriculture	size	rice paddy area size	past; present		invertebrates; amphibians	disturbance/habitat change; exploratory	3	2017	2021
food/agriculture	size	rice paddy to forest edge total length	present	15	birds	reintroduction/restoration	1	2019	2019
food/agriculture	size	scrub pastures size	present		birds	exploratory	1	2007	2007
food/agriculture	size	tea plantation upstream area	present		invertebrates	disturbance/habitat change	1	2015	2015
food/agriculture	size	vineyards area size	present		birds; herbaceous plants	invasions; exploratory	3	2012	2016
food/agriculture	time	cropland abandonment duration	present		trees/shrubs	exploratory	1	2020	2020
food/agriculture	time	field activities period	present		birds	disturbance/habitat change	1	2011	2011
food/agriculture	time	grazing annual days	present		birds; mammals	disturbance/habitat change	1	2010	2010

food/agriculture	time	orchards years since planting	present		birds	conservation	1	2020	2020
food/agriculture	time	pastures abandonment duration	present		trees/shrubs	exploratory	1	2020	2020
food/agriculture	time	viticulture age class	present	1	invertebrates	food/economics	1	2013	2013
human presence	density/count	anthropogenic areas percent	present		mammals	reintroduction/restoration; conflict/collisions	2	2020	2021
human presence	density/count	anthropogenic habitat percent	present		mammals	reintroduction/restoration; conservation	2	2021	2021
human presence	density/count	human-dominated areas percent	present		birds	exploratory	1	2015	2015
human presence	density/count	human features density	present		mammals	food/economics	1	2015	2015
human presence	density/count	human land use percent	past; present	15	mammals	disturbance/habitat change	1	2016	2016
human presence	density/count	human populated areas density	present		mammals	food/economics	1	2014	2014
human presence	density/count	moved in yr1950-1990 percent	present		trees/shrubs	invasions	1	2018	2018
human presence	descriptive	anthrome class	present	1	birds	reintroduction/restoration	1	2017	2017
human presence	descriptive	anthropogenic activity type	present		mammals	disturbance/habitat change	1	2015	2015
human presence	descriptive	anthropogenic biome	present		invertebrates	human health/safety	1	2013	2013
human presence	descriptive	anthropogenic elements	present		mammals	exploratory	1	2009	2009
human presence	descriptive	anthropogenic land	present		birds	conservation	2	2017	2019
human presence	descriptive	anthropogenic land historic	present		amphibians; reptiles	conservation	1	2020	2020
human presence	descriptive	anthropogenic land percent	present		microorganisms; mammals	exploratory; conservation	2	2013	2019
human presence	descriptive	human-dominated landscape	present		mammals	conservation	1	2012	2012
human presence	descriptive	human areas	present		mammals	conservation	1	2016	2016
human presence	descriptive	human influence and NE expansion status	present		mammals	exploratory	1	2014	2014
human presence	descriptive	human influence and NW expansion status	present		mammals	exploratory	1	2014	2014

human presence	descriptive	human land use	present	15	mammals	exploratory; reintroduction/restoration	3	2013	2015
human presence	descriptive	human presence	present		mammals	conservation	1	2017	2017
human presence	distance	anthropogenic areas distance	present		mammals	conflict/collisions	1	2020	2020
human presence	distance	anthropogenic distance	present		invertebrates	human health/safety	1	2018	2018
human presence	distance	anthropogenic influence distance	present		mammals	disturbance/habitat change	1	2021	2021
human presence	distance	human-dominated areas distance	present		mammals	conservation	1	2017	2017
human presence	distance	human activity distance	present		mammals; birds	exploratory; conservation	6	2006	2021
human presence	distance	human areas distance	present		mammals	conservation	1	2016	2016
human presence	distance	human features distance	present		mammals	food/economics	1	2015	2015
human presence	distance	human influence areas distance	present		mammals	conservation	1	2015	2015
human presence	distance	human land use distance	present	15	reptiles	conservation	1	2020	2020
human presence	distance	human populated areas distance	present		mammals	disturbance/habitat change	1	2019	2019
human presence	index	human activity high	present		invertebrates	human health/safety	1	2017	2017
human presence	index	human activity low	present		invertebrates	human health/safety	1	2017	2017
human presence	index	human activity medium	present		invertebrates	human health/safety	1	2017	2017
human presence	index	human footprint index	past; present; future		birds; mammals; herbaceous plants; reptiles; amphibians; microorganisms; trees/shrubs; invertebrates	invasions; conservation; reintroduction/restoration; exploratory; disturbance/habitat change; conflict/collisions; human health/safety; food/economics	74	2010	2021
human presence	index	human footprint non-linear	present		mammals	exploratory	1	2014	2014

human presence	index	human influence index	present		mammals; herbaceous plants; amphibians; reptiles; trees/shrubs; birds; invertebrates; fish; microorganisms	human health/safety; invasions; exploratory; food/economics; conservation; disturbance/habitat change	36	2010	2021
infrastructure	density/count	agricultural areas and developed areas percent	present		herbaceous plants	invasions	1	2019	2019
infrastructure	density/count	agricultural areas and urban areas percent watershed	present	06; 11	fish	reintroduction/restoration	1	2010	2010
infrastructure	density/count	apartments inhabited count	present		invertebrates	exploratory	1	2006	2006
infrastructure	density/count	apartments percent	present		invertebrates	human health/safety	1	2018	2018
infrastructure	density/count	artificial flooding percent	present		birds	exploratory	1	2020	2020
infrastructure	density/count	artificial land percent	present		birds; mammals	reintroduction/restoration; conservation; invasions	3	2016	2021
infrastructure	density/count	artificial urban areas percent	present	06; 11	trees/shrubs	invasions	1	2021	2021
infrastructure	density/count	buildings area sum	present		trees/shrubs	disturbance/habitat change	1	2015	2015
infrastructure	density/count	buildings count	present		birds	conservation	1	2015	2015
infrastructure	density/count	buildings density	present		mammals; herbaceous plants	exploratory; conflict/collisions; invasions	6	2011	2017
infrastructure	density/count	buildings density 250m radius	present		herbaceous plants	invasions	1	2020	2020
infrastructure	density/count	buildings density 2km radius	present		birds	disturbance/habitat change	1	2012	2012

infrastructure	density/count	buildings paved and vegetation mosaic percent	present		invertebrates	human health/safety	1	2021	2021
infrastructure	density/count	buildings percent	present		invertebrates; reptiles; mammals; birds	conservation; conflict/collisions; exploratory	5	2015	2021
infrastructure	density/count	built-up areas and gardens percent	present		invertebrates	food/economics	1	2013	2013
infrastructure	density/count	built-up areas density	present		mammals	conservation	1	2021	2021
infrastructure	density/count	built-up areas density 50m radius	present		birds	invasions	1	2018	2018
infrastructure	density/count	built-up areas percent	past; present		birds; fish; mammals; invertebrates; microorganisms; trees/shrubs	conservation; exploratory; disturbance/habitat change; invasions; food/economics	16	2007	2021
infrastructure	density/count	built-up areas percent 2500m radius	present		birds	invasions	1	2009	2009
infrastructure	density/count	built-up areas percent 50m radius	present		birds	exploratory; invasions	2	2009	2010
infrastructure	density/count	cities density	present	06; 11	mammals	conservation	1	2012	2012
infrastructure	density/count	cities percent	present	06; 11	amphibians	conservation	1	2015	2015
infrastructure	density/count	commercial and services percent	past; present		fish	conservation	1	2021	2021
infrastructure	density/count	communication towers density	present		birds	conservation	1	2013	2013
infrastructure	density/count	construction sites percent	present		birds	reintroduction/restoration	1	2012	2012
infrastructure	density/count	developed areas age mean	present		herbaceous plants; invertebrates	conservation	1	2011	2011
infrastructure	density/count	developed areas density	present		mammals; fish	conflict/collisions; disturbance/habitat change	2	2013	2018

infrastructure	density/count	developed areas exposed percent	present	fish; invertebrates	conservation	1	2011	2011
infrastructure	density/count	developed areas patch count 100ha radius	present	fish	exploratory	1	2009	2009
infrastructure	density/count	developed areas patch count 100ha radius 100m upstream radius	present	fish	exploratory	1	2009	2009
infrastructure	density/count	developed areas patch count 100ha radius subcatchment radius	present	fish	exploratory	1	2009	2009
infrastructure	density/count	developed areas percent	past; present	fish; birds; microorganisms; invertebrates; mammals; amphibians; trees/shrubs; reptiles; herbaceous plants	conservation; exploratory; disturbance/habitat change; human health/safety; invasions	24	2013	2021
infrastructure	density/count	developed areas percent 1000m radius	present	amphibians; invertebrates	exploratory; conservation	2	2016	2020
infrastructure	density/count	developed areas percent 100m radius	present	amphibians	exploratory; disturbance/habitat change	2	2009	2020
infrastructure	density/count	developed areas percent 100m riparian radius	present	fish	exploratory	1	2009	2009
infrastructure	density/count	developed areas percent 100m upstream radius	present	fish	exploratory	1	2009	2009
infrastructure	density/count	developed areas percent 1km radius	present	amphibians	disturbance/habitat change	1	2009	2009
infrastructure	density/count	developed areas percent 2000m radius	present	amphibians	exploratory	1	2020	2020
infrastructure	density/count	developed areas percent 200m radius	present	amphibians	exploratory	1	2020	2020

infrastructure	density/count	developed areas percent 2km radius	present	amphibians	disturbance/habitat change	1	2009	2009
infrastructure	density/count	developed areas percent 30m radius	present	amphibians	disturbance/habitat change	1	2009	2009
infrastructure	density/count	developed areas percent 5000m radius	present	amphibians	exploratory	1	2020	2020
infrastructure	density/count	developed areas percent 500m radius	present	amphibians	exploratory; disturbance/habitat change	2	2009	2020
infrastructure	density/count	developed areas percent subcatchment radius	present	fish	exploratory	1	2009	2009
infrastructure	density/count	developed open space percent	present	birds; herbaceous plants; invertebrates	exploratory; food/economics	5	2014	2020
infrastructure	density/count	developed open space percent 100m radius	present	fish	reintroduction/restoration	1	2021	2021
infrastructure	density/count	developed open space percent 1215m radius	past; present	birds	conservation	1	2021	2021
infrastructure	density/count	developed open space percent 165m radius	past; present	birds	conservation	1	2021	2021
infrastructure	density/count	developed open space percent 1km radius	present	fish	reintroduction/restoration	1	2021	2021
infrastructure	density/count	developed open space percent 2500m radius	present	birds	invasions	1	2009	2009
infrastructure	density/count	developed open space percent 315m radius	past; present	birds	conservation	1	2021	2021
infrastructure	density/count	developed open space percent 50m radius	present	birds	invasions	1	2009	2009
infrastructure	density/count	developed open space percent 615m radius	past; present	birds	conservation	1	2021	2021
infrastructure	density/count	developed open space percent catchment	present	invertebrates	disturbance/habitat change	1	2017	2017

infrastructure	density/count	developed or exposed area count 100ha radius	present		invertebrates	exploratory	1	2009	2009
infrastructure	density/count	developed or exposed area percent	present		invertebrates	exploratory	1	2009	2009
infrastructure	density/count	developed residential or urban areas percent	present	06; 11	birds	disturbance/habitat change	1	2011	2011
infrastructure	density/count	dike impounded percent	present		birds	exploratory	1	2020	2020
infrastructure	density/count	discontinuous urban fabric percent	present; future	06; 11	invertebrates; birds	disturbance/habitat change; reintroduction/restoration	2	2012	2021
infrastructure	density/count	discontinuous urban fabric percent 1km radius	present	06; 11	birds	disturbance/habitat change	1	2013	2013
infrastructure	density/count	disturbance frequency aboveground	present		herbaceous plants; invertebrates	reintroduction/restoration	1	2008	2008
infrastructure	density/count	disturbance frequency aboveground^2	present		herbaceous plants; invertebrates	reintroduction/restoration	1	2008	2008
infrastructure	density/count	disturbance frequency belowground	present		herbaceous plants; invertebrates	reintroduction/restoration	1	2008	2008
infrastructure	density/count	disturbance frequency belowground^2	present		herbaceous plants; invertebrates	reintroduction/restoration	1	2008	2008
infrastructure	density/count	extra-urban miscellaneous percent	present	06; 11	mammals	invasions	1	2013	2013
infrastructure	density/count	factory area 20km radius percent	present		mammals	exploratory	1	2020	2020
infrastructure	density/count	forest and urban edge density	present	06; 11; 15	birds	conservation	1	2005	2005
infrastructure	density/count	forest rural settlements cover percent	present	15	mammals	human health/safety	1	2014	2014

infrastructure	density/count	forest to urban ratio 200m to 20m radius percent	present	06; 11; 15	invertebrates	exploratory	1	2015	2015
infrastructure	density/count	fragmented forest or urban areas percent	present	06; 11; 15	mammals	conservation	1	2011	2011
infrastructure	density/count	house density	present		mammals	conservation; exploratory	2	2016	2017
infrastructure	density/count	houses built yr1950-1990 percent	present		trees/shrubs	invasions	1	2018	2018
infrastructure	density/count	houses in towns count	present		amphibians	conservation	1	2015	2015
infrastructure	density/count	houses percent	present		invertebrates	human health/safety	1	2018	2018
infrastructure	density/count	housing conditions best percent	present	11	invertebrates	human health/safety	1	2019	2019
infrastructure	density/count	housing conditions worst percent	present	11	invertebrates	human health/safety	1	2019	2019
infrastructure	density/count	housing density	past; present; future	11	mammals; birds; invertebrates	disturbance/habitat change; exploratory; human health/safety; invasions	4	2014	2019
infrastructure	density/count	housing percent	present	11	invertebrates	food/economics	1	2013	2013
infrastructure	density/count	human population density settlements	present	11	invertebrates	human health/safety	1	2020	2020
infrastructure	density/count	human population density urban	present	06; 11	mammals	conservation; invasions	2	2017	2018
infrastructure	density/count	impervious surfaces frequency	present		fish	conservation	1	2020	2020
infrastructure	density/count	impervious surfaces percent	present; future		birds; invertebrates; amphibians; reptiles; mammals; herbaceous plants; fish	exploratory; conservation; disturbance/habitat change; human health/safety; food/economics; invasions; conflict/collisions	22	2010	2021

infrastructure	density/count	impervious surfaces percent 2km radius	present		amphibians	exploratory	1	2012	2012
infrastructure	density/count	industrial and military areas percent	past; present		fish	conservation	1	2021	2021
infrastructure	density/count	industrial areas density	present		herbaceous plants	invasions	1	2016	2016
infrastructure	density/count	industrial areas percent	present		amphibians	exploratory	1	2015	2015
infrastructure	density/count	infrastructure and settlements percent	present	06; 09	mammals	conservation	1	2019	2019
infrastructure	density/count	infrastructure percent	present	06; 09	invertebrates; birds	human health/safety; disturbance/habitat change	2	2004	2020
infrastructure	density/count	isolated houses count	present		amphibians	conservation	1	2015	2015
infrastructure	density/count	landfill frequency	present		mammals	invasions	1	2013	2013
infrastructure	density/count	low-medium urban areas percent	past; present	06; 11	fish	conservation	1	2021	2021
infrastructure	density/count	occupied buildings percent	present		mammals	human health/safety	1	2014	2014
infrastructure	density/count	open area and urban edge density	present	06; 11	birds	conservation	1	2005	2005
infrastructure	density/count	pastures close to settlements percent	present		invertebrates	disturbance/habitat change	1	2009	2009
infrastructure	density/count	paved area percent	present		invertebrates	human health/safety	1	2021	2021
infrastructure	density/count	polder cover percent	present		mammals	exploratory	1	2014	2014
infrastructure	density/count	residences count	present		mammals	conservation	1	2015	2015
infrastructure	density/count	residential area 20km radius percent	present		mammals	exploratory	1	2020	2020
infrastructure	density/count	residential areas density	past; present		mammals; birds	disturbance/habitat change; conservation	3	2015	2017
infrastructure	density/count	residential areas low-medium density percent	past; present		fish	conservation	1	2021	2021
infrastructure	density/count	residential areas percent	present; future		invertebrates; herbaceous plants	exploratory; food/economics	2	2013	2021
infrastructure	density/count	roads gravel density	present		mammals	conflict/collisions	1	2017	2017

infrastructure	density/count	roof sheet-steel-made percent	present		mammals	human health/safety	1	2018	2018
infrastructure	density/count	rural areas density	present		birds; mammals; reptiles	conservation	1	2019	2019
infrastructure	density/count	rural areas percent	present		invertebrates	human health/safety	1	2020	2020
infrastructure	density/count	scattered-density urban areas percent	present	06; 11	amphibians	exploratory	1	2015	2015
infrastructure	density/count	settlements and agricultural areas percent	present		herbaceous plants	human health/safety; invasions	2	2009	2013
infrastructure	density/count	settlements and developed areas percent	present		reptiles	exploratory	1	2012	2012
infrastructure	density/count	settlements and urban areas percent	present	06; 11	birds	exploratory	1	2007	2007
infrastructure	density/count	settlements count 25ha radius	present		birds	exploratory	1	2006	2006
infrastructure	density/count	settlements count mean 10km	present		birds	exploratory	1	2006	2006
infrastructure	density/count	settlements dense percent	present		invertebrates	conservation	1	2017	2017
infrastructure	density/count	settlements density	present		mammals; herbaceous plants	conservation; exploratory; invasions; conflict/collisions	7	2011	2021
infrastructure	density/count	settlements density 1000m radius	present		mammals	exploratory	1	2017	2017
infrastructure	density/count	settlements density 2000m radius	present		mammals	exploratory	1	2017	2017
infrastructure	density/count	settlements density 250m radius	present		mammals	exploratory	1	2017	2017
infrastructure	density/count	settlements density 500m radius	present		mammals	exploratory	1	2017	2017
infrastructure	density/count	settlements density weighted	present		mammals	exploratory	1	2020	2020
infrastructure	density/count	settlements largest patch percent 1000m radius	present		mammals	exploratory	1	2017	2017

infrastructure	density/count	settlements largest patch percent 2000m radius	present		mammals	exploratory	1	2017	2017
infrastructure	density/count	settlements largest patch percent 250m radius	present		mammals	exploratory	1	2017	2017
infrastructure	density/count	settlements largest patch percent 500m radius	present		mammals	exploratory	1	2017	2017
infrastructure	density/count	settlements percent	present; future		invertebrates; mammals	human health/safety; exploratory; invasions; conservation	5	2015	2021
infrastructure	density/count	settlements percent 1000m radius	present		mammals	exploratory	1	2017	2017
infrastructure	density/count	settlements percent 2000m radius	present		mammals	exploratory	1	2017	2017
infrastructure	density/count	settlements percent 250m radius	present		mammals	exploratory	1	2017	2017
infrastructure	density/count	settlements percent 500m radius	present		mammals	exploratory	1	2017	2017
infrastructure	density/count	settlements percent 6km radius	present		mammals	exploratory	1	2021	2021
infrastructure	density/count	settlements sparse percent	present		invertebrates	conservation	1	2017	2017
infrastructure	density/count	settlements urban percent	present	06; 11	invertebrates	food/economics	1	2016	2016
infrastructure	density/count	settling lagoons percent	present		amphibians	conservation	1	2015	2015
infrastructure	density/count	streets and roads percent	present		birds	exploratory	1	2011	2011
infrastructure	density/count	suburban areas frequency	present	11	mammals	reintroduction/restoration	1	2021	2021
infrastructure	density/count	suburban areas percent	present	11	invertebrates; birds	human health/safety; exploratory	2	2007	2019
infrastructure	density/count	towns density	present		mammals; birds	conservation; exploratory	2	2004	2021
infrastructure	density/count	towns frequency	present		mammals	conservation	1	2007	2007
infrastructure	density/count	towns or cities percent 10km radius	present	06; 11	mammals	conservation	1	2013	2013
infrastructure	density/count	towns or cities percent 1km radius	present	06; 11	mammals	conservation	1	2013	2013
infrastructure	density/count	towns or cities percent 4km radius	present	06; 11	mammals	conservation	1	2013	2013

infrastructure	density/count	towns or cities percent 7km radius	present	06; 11	mammals	conservation	1	2013	2013
infrastructure	density/count	towns percent	present		amphibians	conservation	1	2015	2015
infrastructure	density/count	transports infrastructure density	present	06; 09	mammals	exploratory	1	2015	2015
infrastructure	density/count	urban-industrial and commercial lands percent	present; future	06; 11	herbaceous plants	disturbance/habitat change	1	2010	2010
infrastructure	density/count	urban and artificial paved roads percent	present	06; 11	amphibians	exploratory	1	2015	2015
infrastructure	density/count	urban and built-up areas percent	present; future	06; 11	fish; herbaceous plants; mammals; birds; invertebrates	conservation; invasions; exploratory; disturbance/habitat change; human health/safety	6	2017	2021
infrastructure	density/count	urban and forest percent 750m radius	present; future	06; 11; 15	birds	conflict/collisions	1	2008	2008
infrastructure	density/count	urban and suburban areas percent 500m radius	present	06; 11	herbaceous plants	invasions	1	2017	2017
infrastructure	density/count	urban areas count	present; future	06; 11	birds	disturbance/habitat change	1	2014	2014
infrastructure	density/count	urban areas density	present	06; 11	mammals; herbaceous plants; reptiles	conservation; invasions; exploratory	4	2012	2016
infrastructure	density/count	urban areas diffuse percent	present	06; 11	invertebrates	invasions	1	2020	2020
infrastructure	density/count	urban areas frequency	present	06; 11	mammals	conservation	1	2020	2020
infrastructure	density/count	urban areas low-density	present	06; 11	reptiles	conflict/collisions	1	2019	2019
infrastructure	density/count	urban areas low-density percent	present	06; 11	invertebrates; amphibians	human health/safety; exploratory	2	2015	2021

infrastructure	density/count	urban areas percent	past; present; future	06; 11	mammals; herbaceous plants; fish; birds; trees/shrubs; invertebrates; amphibians; reptiles; microorganisms	reintroduction/restoration; disturbance/habitat change; exploratory; invasions; conflict/collisions; human health/safety; conservation; food/economics	83	2001	2021
infrastructure	density/count	urban areas percent 1000m radius	present	06; 11	mammals	disturbance/habitat change	1	2019	2019
infrastructure	density/count	urban areas percent 100m radius	present	06; 11	mammals; invertebrates	conflict/collisions; exploratory	2	2015	2019
infrastructure	density/count	urban areas percent 10m radius	present	06; 11	invertebrates	exploratory	1	2015	2015
infrastructure	density/count	urban areas percent 1km radius	present	06; 11	birds	reintroduction/restoration	1	2009	2009
infrastructure	density/count	urban areas percent 2.5km radius	present	06; 11	birds	reintroduction/restoration	1	2009	2009
infrastructure	density/count	urban areas percent 500m radius	present	06; 11	mammals	conflict/collisions; disturbance/habitat change	2	2019	2019
infrastructure	density/count	urban areas percent 5km radius	present	06; 11	birds	reintroduction/restoration	1	2009	2009
infrastructure	density/count	urban areas percent catchment	present	06; 11	fish	reintroduction/restoration; conservation; disturbance/habitat change	4	2014	2021
infrastructure	density/count	urban areas percent lake catchment	present	06; 11	fish	invasions	1	2016	2016
infrastructure	density/count	urban areas percent local	present	06; 11	invertebrates	conservation	1	2015	2015
infrastructure	density/count	urban areas percent local watershed	present	06; 11	fish	conservation	1	2020	2020

infrastructure	density/count	urban areas percent mean 10km	present	06; 11	birds	exploratory	1	2006	2006
infrastructure	density/count	urban areas percent mean 600m radius	present	06; 11	herbaceous plants	invasions	1	2015	2015
infrastructure	density/count	urban areas percent upstream watershed	present	06; 11	fish	conservation	1	2020	2020
infrastructure	density/count	urban areas percent watershed	present	06; 11	herbaceous plants	invasions	1	2010	2010
infrastructure	density/count	urban areas percent watershed mean	present	06; 11	herbaceous plants	invasions	1	2010	2010
infrastructure	density/count	urban areas percent whole	present	06; 11	invertebrates	conservation	1	2015	2015
infrastructure	density/count	urban artificial areas percent	present	06; 11	amphibians	exploratory	1	2015	2015
infrastructure	density/count	urban brownfields >25yrs old percent 100m radius	present	06; 11	herbaceous plants; invertebrates	conservation	1	2011	2011
infrastructure	density/count	urban brownfields >25yrs old percent 200m radius	present	06; 11	herbaceous plants; invertebrates	conservation	1	2011	2011
infrastructure	density/count	urban brownfields >25yrs old percent 25m radius	present	06; 11	herbaceous plants; invertebrates	conservation	1	2011	2011
infrastructure	density/count	urban brownfields >25yrs old percent 50m radius	present	06; 11	herbaceous plants; invertebrates	conservation	1	2011	2011
infrastructure	density/count	urban brownfields 0-11yrs old percent 100m radius	present	06; 11	herbaceous plants; invertebrates	conservation	1	2011	2011
infrastructure	density/count	urban brownfields 0-11yrs old percent 200m radius	present	06; 11	herbaceous plants; invertebrates	conservation	1	2011	2011

infrastructure	density/count	urban brownfields 0-11yrs old percent 25m radius	present	06; 11	herbaceous plants; invertebrates	conservation	1	2011	2011
infrastructure	density/count	urban brownfields 0-11yrs old percent 50m radius	present	06; 11	herbaceous plants; invertebrates	conservation	1	2011	2011
infrastructure	density/count	urban brownfields 0-4yrs old percent 100m radius	present	06; 11	herbaceous plants; invertebrates	conservation	1	2011	2011
infrastructure	density/count	urban brownfields 0-4yrs old percent 200m radius	present	06; 11	herbaceous plants; invertebrates	conservation	1	2011	2011
infrastructure	density/count	urban brownfields 0-4yrs old percent 25m radius	present	06; 11	herbaceous plants; invertebrates	conservation	1	2011	2011
infrastructure	density/count	urban brownfields 0-4yrs old percent 50m radius	present	06; 11	herbaceous plants; invertebrates	conservation	1	2011	2011
infrastructure	density/count	urban brownfields 0-6yrs old percent 100m radius	present	06; 11	herbaceous plants; invertebrates	conservation	1	2011	2011
infrastructure	density/count	urban brownfields 0-6yrs old percent 200m radius	present	06; 11	herbaceous plants; invertebrates	conservation	1	2011	2011
infrastructure	density/count	urban brownfields 0-6yrs old percent 25m radius	present	06; 11	herbaceous plants; invertebrates	conservation	1	2011	2011
infrastructure	density/count	urban brownfields 0-6yrs old percent 50m radius	present	06; 11	herbaceous plants; invertebrates	conservation	1	2011	2011
infrastructure	density/count	urban brownfields 0-8yrs old percent 100m radius	present	06; 11	herbaceous plants; invertebrates	conservation	1	2011	2011

infrastructure	density/count	urban brownfields 0-8yrs old percent 200m radius	present	06; 11	herbaceous plants; invertebrates	conservation	1	2011	2011
infrastructure	density/count	urban brownfields 0-8yrs old percent 25m radius	present	06; 11	herbaceous plants; invertebrates	conservation	1	2011	2011
infrastructure	density/count	urban brownfields 0-8yrs old percent 50m radius	present	06; 11	herbaceous plants; invertebrates	conservation	1	2011	2011
infrastructure	density/count	urban brownfields 10-20yrs old percent 100m radius	present	06; 11	herbaceous plants; invertebrates	conservation	1	2011	2011
infrastructure	density/count	urban brownfields 10-20yrs old percent 200m radius	present	06; 11	herbaceous plants; invertebrates	conservation	1	2011	2011
infrastructure	density/count	urban brownfields 10-20yrs old percent 25m radius	present	06; 11	herbaceous plants; invertebrates	conservation	1	2011	2011
infrastructure	density/count	urban brownfields 10-20yrs old percent 50m radius	present	06; 11	herbaceous plants; invertebrates	conservation	1	2011	2011
infrastructure	density/count	urban brownfields 15-25yrs old percent 100m radius	present	06; 11	herbaceous plants; invertebrates	conservation	1	2011	2011
infrastructure	density/count	urban brownfields 15-25yrs old percent 200m radius	present	06; 11	herbaceous plants; invertebrates	conservation	1	2011	2011
infrastructure	density/count	urban brownfields 15-25yrs old percent 25m radius	present	06; 11	herbaceous plants; invertebrates	conservation	1	2011	2011
infrastructure	density/count	urban brownfields 15-25yrs old percent 50m radius	present	06; 11	herbaceous plants; invertebrates	conservation	1	2011	2011

infrastructure	density/count	urban brownfields 25-30yrs old percent 100m radius	present	06; 11	herbaceous plants; invertebrates	conservation	1	2011	2011
infrastructure	density/count	urban brownfields 25-30yrs old percent 200m radius	present	06; 11	herbaceous plants; invertebrates	conservation	1	2011	2011
infrastructure	density/count	urban brownfields 25-30yrs old percent 25m radius	present	06; 11	herbaceous plants; invertebrates	conservation	1	2011	2011
infrastructure	density/count	urban brownfields 25-30yrs old percent 50m radius	present	06; 11	herbaceous plants; invertebrates	conservation	1	2011	2011
infrastructure	density/count	urban brownfields 6-11yrs old percent 100m radius	present	06; 11	herbaceous plants; invertebrates	conservation	1	2011	2011
infrastructure	density/count	urban brownfields 6-11yrs old percent 200m radius	present	06; 11	herbaceous plants; invertebrates	conservation	1	2011	2011
infrastructure	density/count	urban brownfields 6-11yrs old percent 25m radius	present	06; 11	herbaceous plants; invertebrates	conservation	1	2011	2011
infrastructure	density/count	urban brownfields 6-11yrs old percent 50m radius	present	06; 11	herbaceous plants; invertebrates	conservation	1	2011	2011
infrastructure	density/count	urban brownfields densely-vegetated percent 100m radius	present	06; 11	herbaceous plants; invertebrates	conservation	1	2011	2011
infrastructure	density/count	urban brownfields densely-vegetated percent 200m radius	present	06; 11	herbaceous plants; invertebrates	conservation	1	2011	2011
infrastructure	density/count	urban brownfields densely-vegetated percent 25m radius	present	06; 11	herbaceous plants; invertebrates	conservation	1	2011	2011

infrastructure	density/count	urban brownfields densely-vegetated percent 50m radius	present	06; 11	herbaceous plants; invertebrates	conservation	1	2011	2011
infrastructure	density/count	urban brownfields dry percent 100m radius	present	06; 11	invertebrates	exploratory	1	2006	2006
infrastructure	density/count	urban brownfields dry percent 125m radius	present	06; 11	invertebrates	exploratory	1	2006	2006
infrastructure	density/count	urban brownfields dry percent 25m radius	present	06; 11	invertebrates	exploratory	1	2006	2006
infrastructure	density/count	urban brownfields dry percent 50m radius	present	06; 11	invertebrates	exploratory	1	2006	2006
infrastructure	density/count	urban brownfields dry percent 75m radius	present	06; 11	invertebrates	exploratory	1	2006	2006
infrastructure	density/count	urban brownfields high vegetation percent 100m radius	present	06; 11	herbaceous plants; invertebrates	conservation	1	2011	2011
infrastructure	density/count	urban brownfields high vegetation percent 200m radius	present	06; 11	herbaceous plants; invertebrates	conservation	1	2011	2011
infrastructure	density/count	urban brownfields high vegetation percent 25m radius	present	06; 11	herbaceous plants; invertebrates	conservation	1	2011	2011
infrastructure	density/count	urban brownfields high vegetation percent 50m radius	present	06; 11	herbaceous plants; invertebrates	conservation	1	2011	2011
infrastructure	density/count	urban brownfields low vegetation percent 100m radius	present	06; 11	herbaceous plants; invertebrates	conservation	1	2011	2011
infrastructure	density/count	urban brownfields low vegetation percent 200m radius	present	06; 11	herbaceous plants; invertebrates	conservation	1	2011	2011
infrastructure	density/count	urban brownfields low vegetation percent 25m radius	present	06; 11	herbaceous plants; invertebrates	conservation	1	2011	2011

infrastructure	density/count	urban brownfields low vegetation percent 50m radius	present	06; 11	herbaceous plants; invertebrates	conservation	1	2011	2011
infrastructure	density/count	urban brownfields open percent 100m radius	present	06; 11	invertebrates	exploratory	1	2006	2006
infrastructure	density/count	urban brownfields open percent 125m radius	present	06; 11	invertebrates	exploratory	1	2006	2006
infrastructure	density/count	urban brownfields open percent 25m radius	present	06; 11	invertebrates	exploratory	1	2006	2006
infrastructure	density/count	urban brownfields open percent 50m radius	present	06; 11	invertebrates	exploratory	1	2006	2006
infrastructure	density/count	urban brownfields open percent 75m radius	present	06; 11	invertebrates	exploratory	1	2006	2006
infrastructure	density/count	urban brownfields percent 100m radius	present	06; 11	herbaceous plants; invertebrates	conservation; exploratory	2	2006	2011
infrastructure	density/count	urban brownfields percent 125m radius	present	06; 11	invertebrates	exploratory	1	2006	2006
infrastructure	density/count	urban brownfields percent 200m radius	present	06; 11	herbaceous plants; invertebrates	conservation	1	2011	2011
infrastructure	density/count	urban brownfields percent 25m radius	present	06; 11	herbaceous plants; invertebrates	conservation; exploratory	2	2006	2011
infrastructure	density/count	urban brownfields percent 50m radius	present	06; 11	herbaceous plants; invertebrates	conservation; exploratory	2	2006	2011
infrastructure	density/count	urban brownfields percent 75m radius	present	06; 11	invertebrates	exploratory	1	2006	2006
infrastructure	density/count	urban brownfields sparsely-vegetated percent 100m radius	present	06; 11	herbaceous plants; invertebrates	conservation	1	2011	2011

infrastructure	density/count	urban brownfields sparsely-vegetated percent 200m radius	present	06; 11	herbaceous plants; invertebrates	conservation	1	2011	2011
infrastructure	density/count	urban brownfields sparsely-vegetated percent 25m radius	present	06; 11	herbaceous plants; invertebrates	conservation	1	2011	2011
infrastructure	density/count	urban brownfields sparsely-vegetated percent 50m radius	present	06; 11	herbaceous plants; invertebrates	conservation	1	2011	2011
infrastructure	density/count	urban brownfields wet percent 100m radius	present	06; 11	invertebrates	exploratory	1	2006	2006
infrastructure	density/count	urban brownfields wet percent 125m radius	present	06; 11	invertebrates	exploratory	1	2006	2006
infrastructure	density/count	urban brownfields wet percent 25m radius	present	06; 11	invertebrates	exploratory	1	2006	2006
infrastructure	density/count	urban brownfields wet percent 50m radius	present	06; 11	invertebrates	exploratory	1	2006	2006
infrastructure	density/count	urban brownfields wet percent 75m radius	present	06; 11	invertebrates	exploratory	1	2006	2006
infrastructure	density/count	urban brownfields with forest percent 100m radius	present	06; 11; 15	invertebrates	exploratory	1	2006	2006
infrastructure	density/count	urban brownfields with forest percent 125m radius	present	06; 11; 15	invertebrates	exploratory	1	2006	2006
infrastructure	density/count	urban brownfields with forest percent 25m radius	present	06; 11; 15	invertebrates	exploratory	1	2006	2006
infrastructure	density/count	urban brownfields with forest percent 50m radius	present	06; 11; 15	invertebrates	exploratory	1	2006	2006
infrastructure	density/count	urban brownfields with forest percent 75m radius	present	06; 11; 15	invertebrates	exploratory	1	2006	2006

infrastructure	density/count	urban green space percent	present; future	06; 11	invertebrates; birds	disturbance/habitat change; reintroduction/restoration	3	2012	2021
infrastructure	density/count	urban habitats percent	past; present; future	06; 11	birds	disturbance/habitat change	1	2010	2010
infrastructure	density/count	urban infrastructure percent	present	06; 09; 11	mammals	reintroduction/restoration	1	2018	2018
infrastructure	density/count	urban non-vegetated areas percent	present	06; 11	amphibians	exploratory	1	2015	2015
infrastructure	density/count	urban or industrial area percent 100m radius	present	06; 11	invertebrates	exploratory	1	2015	2015
infrastructure	density/count	urban or industrial area percent 10m radius	present	06; 11	invertebrates	exploratory	1	2015	2015
infrastructure	density/count	urban parks number habitats 100m radius	present	06; 11	birds	food/economics	1	2005	2005
infrastructure	density/count	urban parks number habitats 150m radius	present	06; 11	birds	food/economics	1	2005	2005
infrastructure	density/count	urban parks number habitats 200m radius	present	06; 11	birds	food/economics	1	2005	2005
infrastructure	density/count	urban parks number habitats 250m radius	present	06; 11	birds	food/economics	1	2005	2005
infrastructure	density/count	urban parks number habitats 50m radius	present	06; 11	birds	food/economics	1	2005	2005
infrastructure	density/count	urban parks percent	present; future	06; 11	invertebrates	exploratory	1	2021	2021
infrastructure	density/count	urban polygons count	present	06; 11	mammals	reintroduction/restoration	1	2002	2002
infrastructure	density/count	urban rural areas percent	present	06; 11	trees/shrubs	invasions	1	2017	2017
infrastructure	density/count	urban woody vegetation or urban crops percent	present	02; 06; 11	amphibians	exploratory	1	2015	2015

infrastructure	density/count	urban woody vegetation percent	present	06; 11	amphibians	exploratory	1	2015	2015
infrastructure	density/count	vacant buildings percent	present		mammals	human health/safety	1	2014	2014
infrastructure	density/count	villages count	present		mammals	exploratory	1	2003	2003
infrastructure	density/count	villages density	present		mammals; birds; herbaceous plants	conservation; exploratory; food/economics	6	2012	2021
infrastructure	density/count	villages density 5km radius	present		birds	conservation	1	2017	2017
infrastructure	density/count	villages frequency	present		mammals	conservation	1	2007	2007
infrastructure	density/count	villages percent	present		amphibians	conservation	1	2015	2015
infrastructure	density/count	villages uninhabited per district count	present		trees/shrubs	food/economics	1	2016	2016
infrastructure	density/count	villages with <500 residents count	present		trees/shrubs	food/economics	1	2016	2016
infrastructure	density/count	water and urban edge density	present	06; 11	birds	conservation	1	2005	2005
infrastructure	density/count	weir density 9km radius	present		mammals	invasions	1	2020	2020
infrastructure	descriptive	anthropogenic disturbance index	present		mammals	disturbance/habitat change	1	2020	2020
infrastructure	descriptive	anthropogenic disturbance percent	present		birds	exploratory	1	2007	2007
infrastructure	descriptive	anthropogenic disturbance yr2000 percent	present		birds	exploratory	1	2007	2007
infrastructure	descriptive	artificial embankment structures	present		invertebrates	reintroduction/restoration	1	2007	2007
infrastructure	descriptive	artificial waterbodies	present		birds	exploratory	1	2012	2012
infrastructure	descriptive	artificial waterbodies with quarrying	present		birds	exploratory	1	2012	2012
infrastructure	descriptive	buildings	present		reptiles; trees/shrubs; mammals	invasions; exploratory	5	2013	2018
infrastructure	descriptive	buildings within 100m radius	present		birds	food/economics	1	2012	2012
infrastructure	descriptive	buildings within 25m radius	present		birds	food/economics	1	2012	2012

infrastructure	descriptive	built-up areas	past; present; future		mammals; reptiles; birds; invertebrates	disturbance/habitat change; invasions; exploratory; human health/safety	10	2007	2021
infrastructure	descriptive	built-up areas 10m radius	present		invertebrates	exploratory	1	2020	2020
infrastructure	descriptive	built-up areas 500m radius	present		birds	food/economics	1	2020	2020
infrastructure	descriptive	built-up areas edge	present		invertebrates	exploratory	1	2020	2020
infrastructure	descriptive	built-up subbasin	present		fish	conservation	1	2018	2018
infrastructure	descriptive	built-up upstream	present		fish	conservation	1	2018	2018
infrastructure	descriptive	church architecture material type	present		birds	conservation	1	2020	2020
infrastructure	descriptive	cities	present	06; 11	invertebrates	human health/safety	1	2007	2007
infrastructure	descriptive	continuous urban fabric	present	06; 11	invertebrates	food/economics	1	2018	2018
infrastructure	descriptive	developed areas	present		fish; invertebrates; mammals	disturbance/habitat change; exploratory	3	2012	2018
infrastructure	descriptive	developed areas low-intensity class 21-22 percent 100m radius	present	1	fish	reintroduction/restoration	1	2021	2021
infrastructure	descriptive	developed areas low-intensity class 21-22 percent 1km radius	present	1	fish	reintroduction/restoration	1	2021	2021
infrastructure	descriptive	developed areas low-intensity class 23 percent 100m radius	present	1	fish	reintroduction/restoration	1	2021	2021
infrastructure	descriptive	developed areas low-intensity class 23 percent 1km radius	present	1	fish	reintroduction/restoration	1	2021	2021
infrastructure	descriptive	developed areas open low	present		birds	conservation	1	2017	2017
infrastructure	descriptive	developed areas open low medium	present		birds	conservation	1	2017	2017
infrastructure	descriptive	developed areas roads and deciduous woodland	present		birds	conservation	1	2013	2013

infrastructure	descriptive	developed open space	present		birds	exploratory	1	2016	2016
infrastructure	descriptive	developed open space stream segment percent	present		invertebrates	disturbance/habitat change	1	2017	2017
infrastructure	descriptive	developed open spaces	present		birds	exploratory	1	2019	2019
infrastructure	descriptive	discontinuous urban fabric	present	06; 11	invertebrates	food/economics	1	2021	2021
infrastructure	descriptive	disturbance class	present	1	herbaceous plants	exploratory	1	2018	2018
infrastructure	descriptive	disturbance low	present		birds	disturbance/habitat change	1	2011	2011
infrastructure	descriptive	disturbance moderate	present		birds	disturbance/habitat change	1	2011	2011
infrastructure	descriptive	disturbance type	present		herbaceous plants; invertebrates	conservation	1	2011	2011
infrastructure	descriptive	exurban areas	present		mammals	disturbance/habitat change	1	2016	2016
infrastructure	descriptive	flow regime disturbance index	present		fish	exploratory	1	2016	2016
infrastructure	descriptive	forest disturbance percent 120m radius	present	15	mammals	disturbance/habitat change	1	2017	2017
infrastructure	descriptive	forest disturbance percent 1500m radius	present	15	reptiles	conservation	1	2021	2021
infrastructure	descriptive	forest disturbance percent 150m radius	present	15	reptiles	conservation	1	2021	2021
infrastructure	descriptive	forest disturbance percent 3000m radius	present	15	reptiles	conservation	1	2021	2021
infrastructure	descriptive	forest disturbance percent 300m radius	present	15	reptiles	conservation	1	2021	2021
infrastructure	descriptive	forest disturbance presence	present	15	herbaceous plants	invasions	1	2015	2015
infrastructure	descriptive	forest disturbance within 0-10yrs percent 1500m radius	present	15	reptiles	conservation	1	2021	2021

infrastructure	descriptive	forest disturbance within 0-10yrs percent 300m radius	present	15	reptiles	conservation	1	2021	2021
infrastructure	descriptive	forest disturbance within 10-20yrs percent 1500m radius	present	15	reptiles	conservation	1	2021	2021
infrastructure	descriptive	forest disturbance within 10-20yrs percent 300m radius	present	15	reptiles	conservation	1	2021	2021
infrastructure	descriptive	forest disturbance within 20-30yrs percent 1500m radius	present	15	reptiles	conservation	1	2021	2021
infrastructure	descriptive	forest disturbance within 20-30yrs percent 300m radius	present	15	reptiles	conservation	1	2021	2021
infrastructure	descriptive	forest logging or disturbance intensity	present	15	amphibians; birds; mammals; reptiles	exploratory	1	2002	2002
infrastructure	descriptive	garden greenhouses	present		birds	exploratory	1	2012	2012
infrastructure	descriptive	high-density urban areas	present	06; 11	reptiles	conflict/collisions	1	2019	2019
infrastructure	descriptive	historic urban brownfields age in yr2003	present	06; 11	herbaceous plants; invertebrates	conservation	1	2011	2011
infrastructure	descriptive	horticulture greenhouses	present		birds	exploratory	1	2012	2012
infrastructure	descriptive	housing 25m radius	present	11	invertebrates	food/economics	1	2013	2013
infrastructure	descriptive	housing 50m radius	present	11	invertebrates	food/economics	1	2013	2013
infrastructure	descriptive	human development	present		mammals	conflict/collisions	1	2010	2010
infrastructure	descriptive	human disturbance	present		mammals	conservation; conflict/collisions	2	2013	2017
infrastructure	descriptive	human disturbance index	present		mammals; fish; birds	conflict/collisions; conservation; disturbance/habitat change	5	2006	2020

infrastructure	descriptive	human disturbance index 4km radius	present		birds	reintroduction/restoration	1	2018	2018
infrastructure	descriptive	human disturbance index catchment	present		fish	conservation	1	2019	2019
infrastructure	descriptive	human disturbance index upstream	present		fish	conservation	1	2019	2019
infrastructure	descriptive	human shoreline development	present		birds	disturbance/habitat change	1	2015	2015
infrastructure	descriptive	industrial and commercial areas	present		invertebrates	human health/safety	1	2017	2017
infrastructure	descriptive	industrial and commercial units percent	present		birds	reintroduction/restoration	1	2012	2012
infrastructure	descriptive	industrial areas	present		birds	conservation	1	2011	2011
infrastructure	descriptive	industrial sites	present		herbaceous plants	invasions	1	2017	2017
infrastructure	descriptive	infrastructure	present	06; 09	invertebrates	exploratory	1	2015	2015
infrastructure	descriptive	land ownership	present		trees/shrubs	invasions	1	2018	2018
infrastructure	descriptive	lawn short cut	present; future		birds	conservation	1	2019	2019
infrastructure	descriptive	low-intensity developed areas	present		mammals	disturbance/habitat change	1	2020	2020
infrastructure	descriptive	military restricted areas	present		invertebrates	conservation	1	2014	2014
infrastructure	descriptive	military training areas	present		mammals	exploratory	1	2017	2017
infrastructure	descriptive	ratio forest 200m to urban 20m radius	present	06; 11; 15	invertebrates	exploratory	1	2015	2015
infrastructure	descriptive	recent disturbance by logging	present		invertebrates	disturbance/habitat change	1	2006	2006
infrastructure	descriptive	residential and urban areas	present	06; 11	invertebrates	human health/safety	1	2017	2017
infrastructure	descriptive	residential area yr2000 100m radius	present		herbaceous plants	invasions	1	2012	2012
infrastructure	descriptive	residential area yr2000 500m radius	present		herbaceous plants	invasions	1	2012	2012

infrastructure	descriptive	residential area yr2000 or yr1990 500m radius	present		herbaceous plants	invasions	1	2012	2012
infrastructure	descriptive	residential areas	present		mammals	exploratory	1	2018	2018
infrastructure	descriptive	roads disturbance index	present		birds	exploratory	1	2007	2007
infrastructure	descriptive	rural areas	present		mammals	disturbance/habitat change	1	2016	2016
infrastructure	descriptive	settlements	present		mammals; invertebrates	conservation; exploratory; food/economics; reintroduction/restoration; disturbance/habitat change	6	2004	2020
infrastructure	descriptive	sparsely populated areas	present		invertebrates	human health/safety	1	2017	2017
infrastructure	descriptive	street	present		birds	conservation	1	2012	2012
infrastructure	descriptive	suburban areas	present	11	trees/shrubs	invasions	1	2018	2018
infrastructure	descriptive	suburban or urban areas	present	06; 11	mammals	disturbance/habitat change	1	2016	2016
infrastructure	descriptive	suburban slums	present	11	invertebrates	human health/safety	1	2018	2018
infrastructure	descriptive	tire storage depots	present		invertebrates	human health/safety	1	2018	2018
infrastructure	descriptive	town	present		invertebrates	exploratory	1	2014	2014
infrastructure	descriptive	towns and settlements	present		mammals	disturbance/habitat change	1	2010	2010
infrastructure	descriptive	train depots abandoned	present		invertebrates	human health/safety	1	2018	2018
infrastructure	descriptive	undeveloped privately-owned areas	present		mammals	disturbance/habitat change	1	2016	2016
infrastructure	descriptive	urban-affected fires presence	present	06; 11	birds	reintroduction/restoration	1	2017	2017
infrastructure	descriptive	urban and built-up areas	present; future	06; 11	mammals; herbaceous plants; birds	disturbance/habitat change; invasions; exploratory	3	2010	2021
infrastructure	descriptive	urban and industrial areas	present	06; 11	birds; reptiles	conservation; exploratory	2	2007	2017

infrastructure	descriptive	urban areas	past; present; future	06; 11	herbaceous plants; mammals; invertebrates; microorganisms; trees/shrubs; birds; fish	human health/safety; conservation; disturbance/habitat change; exploratory; reintroduction/restoration; invasions	24	2009	2021
infrastructure	descriptive	urban areas 500m radius	present	06; 11	mammals	conservation; exploratory	2	2013	2021
infrastructure	descriptive	urban areas gyrate	present	06; 11	mammals	conservation	1	2016	2016
infrastructure	descriptive	urban areas or landscape local	present	06; 11	invertebrates	exploratory	1	2015	2015
infrastructure	descriptive	urban brownfields soil type	present	06; 11; 15	herbaceous plants; invertebrates	exploratory	1	2009	2009
infrastructure	descriptive	urban elements	present	06; 11	mammals	exploratory	1	2020	2020
infrastructure	descriptive	urban green space	present	06; 11	invertebrates	food/economics	1	2018	2018
infrastructure	descriptive	vacant housing units percent	present	11	invertebrates	human health/safety	1	2019	2019
infrastructure	descriptive	villages	present		mammals; birds	conservation; exploratory	2	2009	2018
infrastructure	descriptive	wasteland	past; present		mammals	disturbance/habitat change	1	2016	2016
infrastructure	descriptive	wasteland area size	present		invertebrates	exploratory	1	2017	2017
infrastructure	descriptive	water supply type cistern	present	6	invertebrates	human health/safety	1	2018	2018
infrastructure	descriptive	wetlands diked impounded	present	15	birds	conservation	1	2017	2017
infrastructure	descriptive	wetlands excavated	present	15	birds	conservation	1	2017	2017
infrastructure	distance	anthropogenic disturbance distance	present		mammals	conservation; exploratory	2	2021	2021
infrastructure	distance	anthropogenic structures distance	present		birds; mammals	conflict/collisions; disturbance/habitat change	2	2020	2020

infrastructure	distance	artificial ditch distance	present		reptiles	conflict/collisions	1	2021	2021
infrastructure	distance	artificial waterhole distance	present; future		trees/shrubs; mammals	disturbance/habitat change	2	2020	2021
infrastructure	distance	bathing distance	present		invertebrates	human health/safety	1	2018	2018
infrastructure	distance	big settlements distance	present		birds	disturbance/habitat change	1	2012	2012
infrastructure	distance	bridges distance	present		herbaceous plants	invasions	1	2014	2014
infrastructure	distance	buildings distance	past; present		birds; mammals; herbaceous plants	conservation; disturbance/habitat change; exploratory; invasions	9	2007	2021
infrastructure	distance	buildings paved and vegetation mosaic distance	present		invertebrates	human health/safety	1	2021	2021
infrastructure	distance	buildings rural distance	present		birds	disturbance/habitat change	1	2011	2011
infrastructure	distance	built-up areas distance	past; present		herbaceous plants; birds; mammals	invasions; conservation; disturbance/habitat change; exploratory	9	2009	2020
infrastructure	distance	car wash distance	present		invertebrates	human health/safety	1	2018	2018
infrastructure	distance	cities >100k population distance	present	06; 11	amphibians; fish; invertebrates; reptiles	invasions	1	2019	2019
infrastructure	distance	cities >50k population distance	present	06; 11	invertebrates	invasions	1	2021	2021
infrastructure	distance	cities and urban villages distance	present	06; 11	mammals	conservation	1	2020	2020
infrastructure	distance	cities distance	present	06; 11	mammals; birds; reptiles	exploratory; disturbance/habitat change; conservation	9	2012	2021
infrastructure	distance	cities main distance	present	06; 11	birds	conservation	1	2015	2015

infrastructure	distance	cities small distance	present	06; 11	birds	conservation	1	2015	2015
infrastructure	distance	commercial area distance	present		birds	reintroduction/restoration	1	2021	2021
infrastructure	distance	commercial plant nursery distance	present		invertebrates	invasions	1	2019	2019
infrastructure	distance	commercial wood pallet manufacturers distance	present		invertebrates	invasions	1	2019	2019
infrastructure	distance	cottage distance	present		birds	exploratory	1	2005	2005
infrastructure	distance	developed areas distance	present		herbaceous plants; trees/shrubs; mammals	invasions; reintroduction/restoration; disturbance/habitat change; conflict/collisions; conservation	7	2014	2021
infrastructure	distance	developed areas distance 100m radius	present		amphibians	disturbance/habitat change	1	2009	2009
infrastructure	distance	developed areas distance 1km radius	present		amphibians	disturbance/habitat change	1	2009	2009
infrastructure	distance	developed areas distance 2km radius	present		amphibians	disturbance/habitat change	1	2009	2009
infrastructure	distance	developed areas distance 30m radius	present		amphibians	disturbance/habitat change	1	2009	2009
infrastructure	distance	developed areas distance 500m radius	present		amphibians	disturbance/habitat change	1	2009	2009
infrastructure	distance	developed areas high-intensity distance	present		mammals	exploratory	1	2018	2018
infrastructure	distance	developed areas low-intensity distance	present		mammals	exploratory	1	2018	2018
infrastructure	distance	developed open space distance	present		mammals; birds	exploratory; invasions	2	2009	2018
infrastructure	distance	discontinuous urban fabric distance	present	06; 11	mammals	disturbance/habitat change	2	2019	2019

infrastructure	distance	disturbance depth	present		herbaceous plants; invertebrates	reintroduction/restoration	1	2008	2008
infrastructure	distance	disturbance depth^2	present		herbaceous plants; invertebrates	reintroduction/restoration	1	2008	2008
infrastructure	distance	ditching distance	present		invertebrates	human health/safety	1	2018	2018
infrastructure	distance	draining distance	present		invertebrates	human health/safety	1	2018	2018
infrastructure	distance	easements distance	present		birds	conservation	1	2017	2017
infrastructure	distance	factory distance	present		mammals	conservation; exploratory	2	2020	2021
infrastructure	distance	filling distance	present		invertebrates	human health/safety	1	2018	2018
infrastructure	distance	forest disturbance distance	present	15	reptiles	conservation	1	2021	2021
infrastructure	distance	forest disturbance within 0-10yrs distance	present	15	reptiles	conservation	1	2021	2021
infrastructure	distance	forest disturbance within 10-20yrs distance	present	15	reptiles	conservation	1	2021	2021
infrastructure	distance	forest disturbance within 20-30yrs distance	present	15	reptiles	conservation	1	2021	2021
infrastructure	distance	gravel pits distance	present		herbaceous plants	invasions	1	2014	2014
infrastructure	distance	hamlet towns or cities distance	present	06; 11	mammals	disturbance/habitat change	1	2015	2015
infrastructure	distance	house construction activities distance	present		herbaceous plants	invasions	1	2014	2014
infrastructure	distance	house distance	present		mammals; reptiles; birds	exploratory; reintroduction/restoration; disturbance/habitat change	4	2004	2016
infrastructure	distance	houses and roads isolated distance	present		birds	exploratory	1	2012	2012
infrastructure	distance	houses distance	present		invertebrates	human health/safety	1	2019	2019
infrastructure	distance	housing distance	present	11	mammals	conservation	1	2014	2014
infrastructure	distance	human settlements distance	present	11	mammals	conservation	1	2020	2020

infrastructure	distance	impervious surface distance	present		mammals	conservation	1	2020	2020
infrastructure	distance	industrial area distance	present		mammals	conservation	1	2016	2016
infrastructure	distance	industrial facility distance	present		birds	reintroduction/restoration	1	2021	2021
infrastructure	distance	infrastructure distance	present	06; 09	mammals	exploratory	1	2015	2015
infrastructure	distance	landfill distance	present		birds	disturbance/habitat change	1	2018	2018
infrastructure	distance	linear infrastructure distance	present	06; 09	mammals	conservation	1	2015	2015
infrastructure	distance	major cities distance	present	06; 11	microorganisms	disturbance/habitat change	1	2021	2021
infrastructure	distance	major towns and cities distance	present	06; 11	mammals	reintroduction/restoration	1	2021	2021
infrastructure	distance	military base and settlements distance	present		mammals	exploratory	1	2017	2017
infrastructure	distance	military bases distance	present		invertebrates	invasions	1	2019	2019
infrastructure	distance	navy exercise areas distance	present		mammals	conservation	1	2016	2016
infrastructure	distance	public facility distance	present		birds	reintroduction/restoration	1	2021	2021
infrastructure	distance	public roads and villages distance	present		mammals	conservation	1	2013	2013
infrastructure	distance	railways urban areas water bodies distance	present	06; 11	mammals	exploratory	1	2011	2011
infrastructure	distance	residence distance	present		mammals	exploratory	1	2020	2020
infrastructure	distance	residential area distance	past; present		mammals; birds	disturbance/habitat change; conservation; reintroduction/restoration; exploratory	7	2015	2021

infrastructure	distance	residential areas distance	present		birds; mammals; herbaceous plants; invertebrates	reintroduction/restoration; exploratory; conservation; disturbance/habitat change; invasions; human health/safety	9	2009	2021
infrastructure	distance	roads and ditches distances	present		birds	conservation	1	2018	2018
infrastructure	distance	roads and settlements cost distance	present		mammals	exploratory	1	2021	2021
infrastructure	distance	roads and urban areas distance	present	06; 11	birds	conservation	1	2010	2010
infrastructure	distance	roads gravel distance	present		mammals	reintroduction/restoration	1	2019	2019
infrastructure	distance	roads urban distance	present	06; 11	mammals	conservation	1	2009	2009
infrastructure	distance	rural areas distance	present		mammals	disturbance/habitat change	1	2019	2019
infrastructure	distance	rural urban extent	present	06; 11	mammals	human health/safety	1	2021	2021
infrastructure	distance	rural villages distance	present		mammals	conservation	1	2020	2020
infrastructure	distance	settlements and health centers distance	present	3	trees/shrubs	food/economics	1	2019	2019
infrastructure	distance	settlements and large artificial surfaces distance	present		mammals	conflict/collisions	1	2017	2017
infrastructure	distance	settlements and roads distance	present		mammals; herbaceous plants; trees/shrubs	conservation; invasions	2	2013	2021
infrastructure	distance	settlements and roads distance increase 10	future		herbaceous plants; trees/shrubs	invasions	1	2013	2013
infrastructure	distance	settlements and roads distance increase 30	future		herbaceous plants; trees/shrubs	invasions	1	2013	2013

infrastructure	distance	settlements and roads distance stable	future		herbaceous plants; trees/shrubs	invasions	1	2013	2013
infrastructure	distance	settlements and urban areas distance	present	06; 11	birds	exploratory	1	2007	2007
infrastructure	distance	settlements cost distance	present		herbaceous plants	invasions	1	2021	2021
infrastructure	distance	settlements distance	past; present; future		mammals; birds; invertebrates; reptiles; trees/shrubs; fish; herbaceous plants	conflict/collisions; conservation; exploratory; reintroduction/restoration; invasions; disturbance/habitat change; human health/safety; food/economics	65	2008	2021
infrastructure	distance	settlements green and open space distance	present		birds	conflict/collisions	1	2020	2020
infrastructure	distance	settlements or villages distance	present		mammals	reintroduction/restoration	1	2008	2008
infrastructure	distance	settlements permanent distance	present		mammals	conflict/collisions	1	2013	2013
infrastructure	distance	settlements roads distance mean	present		mammals	conservation	1	2006	2006
infrastructure	distance	settlements rural distance	present		birds; reptiles; mammals	exploratory; conservation; invasions	6	2012	2021
infrastructure	distance	settlements small distance	present		birds	disturbance/habitat change	1	2012	2012
infrastructure	distance	settlements temporary distance	present		mammals	conflict/collisions	1	2013	2013
infrastructure	distance	settlements urban distance	present	06; 11	birds; reptiles; mammals; invertebrates	exploratory; conservation; invasions; food/economics	6	2016	2021

infrastructure	distance	suburban areas distance	present	11	mammals; birds	reintroduction/restoration; conflict/collisions; exploratory	3	2007	2021
infrastructure	distance	tower distance	present		birds	disturbance/habitat change	1	2018	2018
infrastructure	distance	towns <100k inhabitants distance	present		birds	invasions	1	2006	2006
infrastructure	distance	towns >100k inhabitants distance	present		invertebrates; amphibians; mammals; birds	conservation; exploratory; invasions	5	2006	2018
infrastructure	distance	towns >500k inhabitants distance	present		invertebrates; amphibians; mammals; birds	conservation; exploratory; invasions	4	2014	2018
infrastructure	distance	towns and settlements distance	present		mammals	disturbance/habitat change	1	2010	2010
infrastructure	distance	towns cost distance	past; present		mammals	conflict/collisions	1	2019	2019
infrastructure	distance	towns distance	present		mammals; birds; herbaceous plants	conflict/collisions; reintroduction/restoration; exploratory; conservation	10	2004	2020
infrastructure	distance	towns or cities distance	present	06; 11	mammals	conservation	1	2013	2013
infrastructure	distance	township distance	present		mammals	conservation	1	2015	2015
infrastructure	distance	universities distance	present		invertebrates	invasions	1	2019	2019
infrastructure	distance	urban and agricultural areas distance	present	06; 11	herbaceous plants	invasions	1	2013	2013
infrastructure	distance	urban and built-up areas distance	present	06; 11	mammals	conservation; disturbance/habitat change	2	2019	2021
infrastructure	distance	urban and industrial areas distance	present	06; 11	herbaceous plants; trees/shrubs	exploratory; invasions	2	2013	2015

infrastructure	distance	urban areas >100k inhabitants distance	present	06; 11	amphibians; birds; mammals; reptiles	exploratory	1	2010	2010
infrastructure	distance	urban areas >500k inhabitants distance	present	06; 11	amphibians; birds; mammals; reptiles	exploratory	1	2010	2010
infrastructure	distance	urban areas adjacency	present	06; 11	herbaceous plants	invasions	1	2010	2010
infrastructure	distance	urban areas adjacency watershed	present	06; 11	herbaceous plants	invasions	1	2010	2010
infrastructure	distance	urban areas adjacency watershed mean	present	06; 11	herbaceous plants	invasions	1	2010	2010
infrastructure	distance	urban areas cost distance actual	present	06; 11	mammals	exploratory	1	2008	2008
infrastructure	distance	urban areas cost distance sum	present	06; 11	mammals	exploratory	1	2008	2008
infrastructure	distance	urban areas distance	present; future	06; 11	mammals; reptiles; trees/shrubs; birds; amphibians; herbaceous plants; invertebrates; fish	reintroduction/restoration; disturbance/habitat change; conservation; invasions; exploratory; conflict/collisions; food/economics; human health/safety	62	2004	2021
infrastructure	distance	urban areas high-intensity distance	present	06; 11	mammals	disturbance/habitat change	1	2019	2019
infrastructure	distance	urban center distance	present	06; 11	microorganisms; mammals; fish	conservation	3	2016	2019
infrastructure	distance	urban coastal areas distance	present	06; 11	mammals	conservation	1	2021	2021

infrastructure	distance	urban distance	present; future	06; 11	amphibians; birds; reptiles; mammals	disturbance/habitat change	2	2018	2021
infrastructure	distance	urban parks distance	present	06; 11	mammals	disturbance/habitat change	2	2019	2019
infrastructure	distance	urban parks distance from forest	present	06; 11; 15	birds	food/economics	1	2005	2005
infrastructure	distance	villages distance	present		mammals; birds; microorganisms; herbaceous plants	conservation; exploratory; disturbance/habitat change; food/economics; invasions; reintroduction/restoration	43	2003	2021
infrastructure	distance	villages distance class	present	1	birds	conservation	1	2006	2006
infrastructure	distance	villages small distance	present		mammals	reintroduction/restoration	1	2009	2009
infrastructure	distance	water abstraction distance	present	6	invertebrates	human health/safety	1	2018	2018
infrastructure	distance	water well ditch or canal distance	present	6	birds	disturbance/habitat change	1	2018	2018
infrastructure	distance	weir distance	present		trees/shrubs	disturbance/habitat change	1	2014	2014
infrastructure	index	artificial land aggregation index	present		birds	reintroduction/restoration	1	2021	2021
infrastructure	index	artificial land clumpiness	present		mammals	invasions	1	2016	2016
infrastructure	index	developed areas cohesion	present		birds	exploratory	1	2011	2011
infrastructure	index	developed areas high-intensity	present		mammals; birds; trees/shrubs	disturbance/habitat change; exploratory; invasions	4	2016	2020
infrastructure	index	developed areas high-intensity percent	present		birds	exploratory	3	2011	2020
infrastructure	index	developed areas high-intensity percent 1km radius	present		fish	reintroduction/restoration	1	2021	2021

infrastructure	index	developed areas high-intensity percent catchment	present	invertebrates	disturbance/habitat change	1	2017	2017
infrastructure	index	developed areas high-intensity stream segment percent	present	invertebrates	disturbance/habitat change	1	2017	2017
infrastructure	index	developed areas high intensity	present	birds	conservation	1	2017	2017
infrastructure	index	developed areas high intensity percent	present	birds	exploratory	1	2018	2018
infrastructure	index	developed areas low-intensity	present	birds	exploratory	1	2019	2019
infrastructure	index	developed areas low-intensity percent	present	birds; herbaceous plants	exploratory	5	2011	2020
infrastructure	index	developed areas low-intensity percent 100m radius	present	fish	reintroduction/restoration	1	2021	2021
infrastructure	index	developed areas low-intensity percent 1km radius	present	fish	reintroduction/restoration	1	2021	2021
infrastructure	index	developed areas low-intensity percent catchment	present	invertebrates	disturbance/habitat change	1	2017	2017
infrastructure	index	developed areas low-intensity stream segment percent	present	invertebrates	disturbance/habitat change	1	2017	2017
infrastructure	index	developed areas low high-intensity	present	trees/shrubs; birds	invasions; exploratory	2	2016	2018
infrastructure	index	developed areas medium-intensity	present	birds	exploratory	2	2016	2019
infrastructure	index	developed areas medium-intensity percent	present	birds	exploratory	2	2018	2020
infrastructure	index	developed areas medium-intensity percent 100m radius	present	fish	reintroduction/restoration	1	2021	2021

infrastructure	index	developed areas medium-intensity percent 1215m radius	past; present	birds	conservation	1	2021	2021
infrastructure	index	developed areas medium-intensity percent 165m radius	past; present	birds	conservation	1	2021	2021
infrastructure	index	developed areas medium-intensity percent 1km radius	present	fish	reintroduction/restoration	1	2021	2021
infrastructure	index	developed areas medium-intensity percent 315m radius	past; present	birds	conservation	1	2021	2021
infrastructure	index	developed areas medium-intensity percent 615m radius	past; present	birds	conservation	1	2021	2021
infrastructure	index	developed areas medium-intensity percent catchment	present	invertebrates	disturbance/habitat change	1	2017	2017
infrastructure	index	developed areas medium-intensity stream segment percent	present	invertebrates	disturbance/habitat change	1	2017	2017
infrastructure	index	developed light intensity percent 1215m radius	past; present	birds	conservation	1	2021	2021
infrastructure	index	developed light intensity percent 165m radius	past; present	birds	conservation	1	2021	2021
infrastructure	index	developed light intensity percent 315m radius	past; present	birds	conservation	1	2021	2021
infrastructure	index	developed light intensity percent 615m radius	past; present	birds	conservation	1	2021	2021
infrastructure	index	disturbance geomorphological	present	fish	reintroduction/restoration	1	2015	2015
infrastructure	index	disturbance index	past	fish	exploratory	1	2016	2016
infrastructure	index	disturbance index change yr1975-1990	present	herbaceous plants	invasions	1	2012	2012

infrastructure	index	disturbance index change yr1990-2000	present		herbaceous plants	invasions	1	2012	2012
infrastructure	index	disturbance index yr1975	present		herbaceous plants	invasions	1	2012	2012
infrastructure	index	disturbance index yr1990	present		herbaceous plants	invasions	1	2012	2012
infrastructure	index	disturbance index yr2000	present		herbaceous plants	invasions	1	2012	2012
infrastructure	index	disturbance intensity	present		herbaceous plants; invertebrates	reintroduction/restoration	1	2008	2008
infrastructure	index	disturbance intensity^2	present		herbaceous plants; invertebrates	reintroduction/restoration	1	2008	2008
infrastructure	index	forest disturbance burning frequency	present	15	birds; herbaceous plants; invertebrates	disturbance/habitat change	1	2016	2016
infrastructure	index	forest disturbance logging frequency	present	15	birds; herbaceous plants; invertebrates	disturbance/habitat change	1	2016	2016
infrastructure	index	forest to urban bushland remnant ratio	present	06; 11; 15	mammals	exploratory	1	2013	2013
infrastructure	index	imperviousness	present		birds; fish; invertebrates	disturbance/habitat change; conservation	3	2018	2021
infrastructure	index	imperviousness attenuated	present		mammals	disturbance/habitat change	1	2014	2014
infrastructure	index	imperviousness attenuated minimum	present		mammals	disturbance/habitat change	1	2014	2014
infrastructure	index	imperviousness total	present		mammals	disturbance/habitat change	1	2014	2014

infrastructure	index	imperviousness total minimum	present		mammals	disturbance/habitat change	1	2014	2014
infrastructure	index	reservoir capacities local	present		fish	invasions	1	2018	2018
infrastructure	index	reservoir capacities upstream	present		trees/shrubs; fish	invasions	2	2018	2018
infrastructure	index	shoreline development index	present		fish	disturbance/habitat change	1	2015	2015
infrastructure	index	street index	present		herbaceous plants	exploratory	1	2018	2018
infrastructure	index	urban areas high-intensity	present	06; 11	mammals	exploratory	1	2017	2017
infrastructure	index	urban areas high-intensity percent	present	06; 11	invertebrates; fish	invasions; conservation	3	2018	2020
infrastructure	index	urban areas high-intensity percent 2km radius	present	06; 11	amphibians	exploratory	1	2012	2012
infrastructure	index	urban areas index	present	06; 11	birds	reintroduction/restoration	1	2017	2017
infrastructure	index	urban areas low-intensity	present	06; 11	mammals; reptiles	exploratory; disturbance/habitat change	2	2006	2017
infrastructure	index	urban areas low-intensity percent	present	06; 11	fish; invertebrates	conservation	2	2018	2019
infrastructure	index	urban areas low-intensity percent 2km radius	present	06; 11	amphibians	exploratory	1	2012	2012
infrastructure	index	urban areas medium-intensity percent	present	06; 11	fish; invertebrates	conservation	2	2018	2019
infrastructure	index	urban brownfields ratio	present	06; 11	herbaceous plants; invertebrates	exploratory	1	2009	2009
infrastructure	index	villages index	present		herbaceous plants	exploratory	1	2018	2018
infrastructure	size	artificial land mean area size	present		birds	reintroduction/restoration	1	2021	2021

infrastructure	size	artificial open space size	past; present		invertebrates	disturbance/habitat change	1	2020	2020
infrastructure	size	buildings area size	past; present		invertebrates; herbaceous plants	disturbance/habitat change; human health/safety; invasions	3	2013	2021
infrastructure	size	buildings height 1000m radius	present		birds	invasions	1	2018	2018
infrastructure	size	buildings height 50m radius	present		birds	invasions	1	2018	2018
infrastructure	size	buildings surface area	present		trees/shrubs	invasions	1	2017	2017
infrastructure	size	built-up areas area-weighted mean	present		mammals	conservation	1	2021	2021
infrastructure	size	built-up areas size	present		invertebrates; mammals	exploratory; invasions	2	2010	2017
infrastructure	size	developed areas high area size	present		invertebrates	human health/safety	1	2021	2021
infrastructure	size	developed areas mean size 100m riparian radius	present		fish	exploratory	1	2009	2009
infrastructure	size	developed areas mean size 100m upstream radius	present		fish	exploratory	1	2009	2009
infrastructure	size	developed areas mean size subcatchment radius	present		fish	exploratory	1	2009	2009
infrastructure	size	developed areas medium area size	present		invertebrates	human health/safety	1	2021	2021
infrastructure	size	developed areas open area size	present		invertebrates	human health/safety	1	2021	2021
infrastructure	size	developed or exposed area patch size mean	present		invertebrates	exploratory	1	2009	2009
infrastructure	size	human population size of nearest town	present	11	herbaceous plants	exploratory	1	2019	2019
infrastructure	size	impervious surfaces area size	present		birds	invasions	2	2019	2019
infrastructure	size	paved area size	present		invertebrates	human health/safety	1	2021	2021
infrastructure	size	property size	present		trees/shrubs	invasions	1	2018	2018
infrastructure	size	residential areas area size	present		mammals	exploratory	1	2011	2011
infrastructure	size	settlements area size	present		birds	exploratory	1	2007	2007

infrastructure	size	settlements area size mean	present		fish	exploratory	1	2020	2020
infrastructure	size	settlements mean size 1000m radius	present		mammals	exploratory	1	2017	2017
infrastructure	size	settlements mean size 2000m radius	present		mammals	exploratory	1	2017	2017
infrastructure	size	settlements mean size 250m radius	present		mammals	exploratory	1	2017	2017
infrastructure	size	settlements mean size 500m radius	present		mammals	exploratory	1	2017	2017
infrastructure	size	street length	present		herbaceous plants	conservation; invasions	2	2013	2018
infrastructure	size	street major length	present		herbaceous plants	invasions	1	2010	2010
infrastructure	size	street minor length	present		herbaceous plants	invasions	1	2010	2010
infrastructure	size	urban areas area-weighted mean	present	06; 11	mammals	conservation	1	2016	2016
infrastructure	size	urban areas size	present	06; 11	microorganisms; birds; invertebrates; herbaceous plants; mammals	conservation; invasions; exploratory; conflict/collisions	7	2011	2020
infrastructure	time	church year built	present		birds	conservation	1	2020	2020
infrastructure	time	forest time since logging or disturbance	present	15	amphibians; birds; mammals; reptiles	exploratory	1	2002	2002
infrastructure	time	housing year built median	present	11	trees/shrubs	invasions	1	2018	2018
infrastructure	time	housing year moved in median	present	11	trees/shrubs	invasions	1	2018	2018
infrastructure	time	time-in-production of property years	present		mammals	disturbance/habitat change	1	2015	2015
infrastructure	time	time since buildings demolition in years	present		invertebrates	exploratory	1	2006	2006

infrastructure	time	transportation travel time to nearest cities by land or sea	present	06; 11	birds; invertebrates	human health/safety	1	2018	2018
infrastructure	time	travel time cities >50k inhabitants	present	06; 11	fish; herbaceous plants; invertebrates; amphibians; birds; mammals; reptiles; trees/shrubs	invasions	1	2019	2019
infrastructure	time	travel time to cities	present	06; 11	herbaceous plants	food/economics	1	2020	2020
infrastructure	time	travel time to major cities	present	06; 11	amphibians; birds; mammals; reptiles	invasions	1	2021	2021
infrastructure	time	urban brownfields age class	present	01; 06; 11	herbaceous plants; invertebrates	exploratory	1	2009	2009
infrastructure	time	urban parks age	present	06; 11	birds	food/economics	1	2005	2005
management/ interventions	density/count	conservation program percent	past; present		birds	disturbance/habitat change	1	2017	2017
management/ interventions	density/count	conservation reserve percent	present		birds	exploratory	1	2017	2017
management/ interventions	density/count	cultivated and managed areas percent	present; future		herbaceous plants; birds	disturbance/habitat change	2	2012	2017
management/ interventions	density/count	cultivated and managed herbaceous graminoids percent	present		amphibians	exploratory	1	2015	2015

management/ interventions	density/count	cultivated and managed herbaceous non-graminoids percent	present		amphibians	exploratory	1	2015	2015
management/ interventions	density/count	cultivated and managed vegetation percent	present		fish; herbaceous plants; invertebrates; mammals	conservation; invasions	4	2019	2021
management/ interventions	density/count	discharge regulation percent	present		fish	reintroduction/restoration	1	2021	2021
management/ interventions	density/count	forest managed percent	present	15	mammals	exploratory	1	2020	2020
management/ interventions	density/count	game reserve percent	present		mammals	conflict/collisions	1	2020	2020
management/ interventions	density/count	improved grassland frequency	present		mammals	reintroduction/restoration	1	2021	2021
management/ interventions	density/count	low or conservation use percent	past; present		birds	exploratory	1	2018	2018
management/ interventions	density/count	managed bamboo forest percent	present	15	reptiles	conflict/collisions	1	2021	2021
management/ interventions	density/count	managed broadleaf forest percent	present	15	reptiles	conflict/collisions	1	2021	2021
management/ interventions	density/count	managed clearing areas percent	present		mammals	conflict/collisions	1	2015	2015
management/ interventions	density/count	managed conifer forest percent	present	15	reptiles	conflict/collisions	1	2021	2021
management/ interventions	density/count	managed grassland percent	present		invertebrates	disturbance/habitat change	1	2019	2019
management/ interventions	density/count	managed grassland percent 1600m radius	present		birds	exploratory	1	2002	2002
management/ interventions	density/count	managed grassland percent 400m radius	present		birds	exploratory	1	2002	2002
management/ interventions	density/count	managed grassland percent 800m radius	present		birds	exploratory	1	2002	2002

management/ interventions	density/count	managed land percent	present		birds	conservation	1	2018	2018
management/ interventions	density/count	managed pastures percent	present; future		mammals; invertebrates	disturbance/habitat change; human health/safety	3	2019	2021
management/ interventions	density/count	managed trees percent	present		invertebrates	conservation	1	2021	2021
management/ interventions	density/count	managed wetland percent	present		birds	conservation	1	2018	2018
management/ interventions	density/count	management pollution discharge elimination system density	present	06; 11; 13	fish	conservation	1	2019	2019
management/ interventions	density/count	management Superfund site density	present		fish	conservation	1	2019	2019
management/ interventions	density/count	management toxic release inventory site density	present		fish	conservation	1	2019	2019
management/ interventions	density/count	nest box density	present		birds	reintroduction/restoration	1	2011	2011
management/ interventions	density/count	protected areas percent	present	14	mammals; invertebrates	reintroduction/restoration; exploratory	2	2015	2018
management/ interventions	density/count	reforested areas percent	present		birds	exploratory	1	2015	2015
management/ interventions	density/count	sites of special scientific interest frequency	present		mammals	reintroduction/restoration	1	2021	2021
management/ interventions	density/count	unprotected land percent 100m radius	present		fish	reintroduction/restoration	1	2021	2021
management/ interventions	density/count	urban forest canopy percent	present	06; 11; 15	mammals	conflict/collisions	1	2015	2015
management/ interventions	density/count	urban forest percent	present	06; 11; 15	invertebrates	disturbance/habitat change	1	2019	2019
management/ interventions	descriptive	artificial regeneration presence	present		herbaceous plants	invasions	1	2015	2015

management/ interventions	descriptive	baiting treatment category	present		mammals	exploratory	1	2020	2020
management/ interventions	descriptive	bird conservation areas	present		birds	exploratory	1	2021	2021
management/ interventions	descriptive	conservation area within 5km radius	present		birds	exploratory	1	2021	2021
management/ interventions	descriptive	conservation status	present		birds	exploratory	1	2020	2020
management/ interventions	descriptive	cultivated and managed areas	present; future		invertebrates	disturbance/habitat change	1	2014	2014
management/ interventions	descriptive	cultivated and managed vegetation	present		invertebrates; herbaceous plants; fish; birds	human health/safety; conservation; food/economics; invasions; exploratory	5	2014	2021
management/ interventions	descriptive	forest management approaches	present	15	microorganisms	disturbance/habitat change	1	2021	2021
management/ interventions	descriptive	forest preserve	present	15	birds	food/economics	1	2012	2012
management/ interventions	descriptive	habitat management class	present	1	birds	exploratory	1	2016	2016
management/ interventions	descriptive	habitat protection class	present	1	mammals	conservation	1	2020	2020
management/ interventions	descriptive	land management type	present	15	invertebrates	conservation	1	2005	2005
management/ interventions	descriptive	managed areas	present		birds	exploratory	1	2020	2020
management/ interventions	descriptive	managed feeding areas presence	present		birds	exploratory	1	2005	2005
management/ interventions	descriptive	managed mixed forest percent	present	15	reptiles	conflict/collisions	1	2021	2021
management/ interventions	descriptive	managed pasture	present		birds	conservation	1	2019	2019
management/ interventions	descriptive	management plot type	present		birds	conservation	1	2017	2017

management/ interventions	descriptive	management regime type	present		invertebrates	food/economics	1	2016	2016
management/ interventions	descriptive	management zone class	present	1	birds	reintroduction/restoration	1	2019	2019
management/ interventions	descriptive	marine park zone type	present	14	mammals	conservation	1	2021	2021
management/ interventions	descriptive	national wilderness and roadless areas	present		mammals	exploratory	1	2015	2015
management/ interventions	descriptive	Native American land	present		birds	conservation	1	2017	2017
management/ interventions	descriptive	Native American trails	past		trees/shrubs	disturbance/habitat change	1	2015	2015
management/ interventions	descriptive	nature reserves	present		mammals	exploratory	2	2017	2018
management/ interventions	descriptive	nature reserves local frequency	present		mammals	reintroduction/restoration	1	2021	2021
management/ interventions	descriptive	nature reserves national frequency	present		mammals	reintroduction/restoration	1	2021	2021
management/ interventions	descriptive	oil gas regulated areas	present		invertebrates	conservation	1	2014	2014
management/ interventions	descriptive	private land protected	present		mammals	disturbance/habitat change	1	2016	2016
management/ interventions	descriptive	private land unprotected	present		mammals	disturbance/habitat change	1	2016	2016
management/ interventions	descriptive	protected areas	present	14	invertebrates; mammals; birds	human health/safety; conservation; disturbance/habitat change	6	2012	2021
management/ interventions	descriptive	protection class	present	1	mammals	conservation	1	2003	2003
management/ interventions	descriptive	protection status	present		mammals	conservation	1	2006	2006
management/ interventions	descriptive	reforestation percent	present	15	trees/shrubs; mammals	invasions; conservation	2	2003	2021

management/ interventions	descriptive	regulated areas	present		mammals	conservation	1	2014	2014
management/ interventions	descriptive	rescinded fish advisories	present		birds	conservation	1	2017	2017
management/ interventions	descriptive	site preparation presence	present		herbaceous plants	invasions	1	2015	2015
management/ interventions	descriptive	tree plantations and managed woods	present	15	mammals	conservation	1	2009	2009
management/ interventions	descriptive	tribal land	present		mammals	disturbance/habitat change	1	2016	2016
management/ interventions	descriptive	water ecological status	present	6	herbaceous plants	exploratory	1	2020	2020
management/ interventions	distance	artificial reef distance	present		invertebrates	reintroduction/restoration	1	2021	2021
management/ interventions	distance	first record distance	present		amphibians; fish; invertebrates; reptiles	invasions	1	2019	2019
management/ interventions	distance	forest management areas distance	present	15	mammals	conservation	1	2018	2018
management/ interventions	distance	historic Native American villages distance	past		trees/shrubs	disturbance/habitat change	1	2015	2015
management/ interventions	distance	improved grassland distance	present		mammals	reintroduction/restoration	1	2021	2021
management/ interventions	distance	management area distance	present		mammals	conservation	1	2016	2016
management/ interventions	distance	management eradication area distance	present		invertebrates	invasions	1	2019	2019
management/ interventions	distance	Native American trails distance	past		trees/shrubs	disturbance/habitat change	1	2015	2015
management/ interventions	distance	nature reserve distance	present		mammals	conservation	1	2015	2015
management/ interventions	distance	non-hunting areas distance	present		mammals	conservation	1	2017	2017

management/ interventions	distance	non-hunting reserve distance	present		mammals	disturbance/habitat change	1	2015	2015
management/ interventions	distance	park security gates and outposts distance	present		mammals	conservation	1	2014	2014
management/ interventions	distance	protected areas distance	present	14	mammals; invertebrates; birds	reintroduction/restoration; exploratory; conservation; conflict/collisions; disturbance/habitat change; invasions; food/economics	17	2009	2021
management/ interventions	distance	protected areas extent	present	14	mammals	disturbance/habitat change	1	2019	2019
management/ interventions	distance	protection area for waterfowl distance	present		invertebrates	invasions	1	2020	2020
management/ interventions	distance	ranger station distance	present		birds	exploratory	1	2014	2014
management/ interventions	distance	research camp distance	present		mammals	disturbance/habitat change	1	2020	2020
management/ interventions	distance	snare hotspots distance	present		mammals	conservation	1	2014	2014
management/ interventions	distance	species introduction site distance	present		invertebrates; herbaceous plants; mammals; birds	invasions; reintroduction/restoration; food/economics	8	2006	2021
management/ interventions	distance	unimproved grasslands distance	present		mammals	exploratory	1	2015	2015
management/ interventions	distance	wilderness areas distance	present		mammals	exploratory	1	2009	2009
management/ interventions	index	forest stand stocking rate	present	15	birds	exploratory	1	2018	2018
management/ interventions	index	gross domestic product percent invested in conservation	present		mammals	conservation	1	2013	2013

	management/ interventions	index	intensive grasslands index	present		amphibians	disturbance/habitat change	1	2020	2020
	management/ interventions	index	managed flow velocity	present		mammals	reintroduction/restoration	1	2021	2021
	management/ interventions	index	management hydrological connectivity	present		amphibians; fish; invertebrates; reptiles	reintroduction/restoration	1	2013	2013
	management/ interventions	index	management plot to crop contrast	present		birds	conservation	1	2017	2017
	management/ interventions	index	protection level	present		herbaceous plants; mammals	exploratory; conservation	2	2013	2017
	management/ interventions	index	stocking rate	present		birds; mammals	disturbance/habitat change	1	2010	2010
	management/ interventions	size	protected areas mean size	present	14	mammals	conservation	1	2021	2021
	management/ interventions	time	annual management date	present		invertebrates	conservation	1	2005	2005
	pollution	density/count	ammonium concentration	present; future		fish	disturbance/habitat change	1	2018	2018
	pollution	density/count	avian lead poisoning	present		birds	conservation	1	2017	2017
	pollution	density/count	avian pesticide death	present		birds	conservation	1	2017	2017
	pollution	density/count	chlorophyll-a maximum	present		fish; herbaceous plants; invertebrates; microorganisms; mammals; reptiles; trees/shrubs	invasions	1	2015	2015

pollution	density/count	chlorophyll-a minimum	present		fish; herbaceous plants; invertebrates; microorganisms; mammals; reptiles; trees/shrubs	invasions	1	2015	2015
pollution	density/count	dump sites percent	present		birds	reintroduction/restoration	1	2012	2012
pollution	density/count	government point-source pollution site density catchment	present	06; 11; 13	fish	reintroduction/restoration	1	2021	2021
pollution	density/count	lead concentration	present		birds	disturbance/habitat change	1	2019	2019
pollution	density/count	mean annual runoff	present; future		invertebrates	conservation	1	2017	2017
pollution	density/count	nitrate concentration	present; future		fish	disturbance/habitat change	1	2018	2018
pollution	density/count	nitrogen concentration	present		herbaceous plants	reintroduction/restoration	1	2020	2020
pollution	density/count	phosphate concentration	present; future		fish	disturbance/habitat change	1	2018	2018
pollution	density/count	phosphate total dissolved	present; future		fish	disturbance/habitat change	1	2018	2018
pollution	density/count	poisoning incidents count 10yrs 5km radius	present		birds	conservation	1	2017	2017
pollution	density/count	pollutant discharge elimination sites density	present		invertebrates	conservation	1	2021	2021
pollution	density/count	pollutants hydrocarbons volume	present		invertebrates	disturbance/habitat change	1	2019	2019
pollution	density/count	pollutants inorganic concentration	present		microorganisms	conservation	1	2020	2020
pollution	density/count	pollutants ocean-based concentration	present	14	microorganisms	conservation	1	2020	2020

pollution	density/count	pollutants organic concentration	present		microorganisms	conservation	1	2020	2020
pollution	density/count	pollutants total tins volume	present		invertebrates	disturbance/habitat change	1	2019	2019
pollution	density/count	polyaromatic hydrocarbons total	present		invertebrates	food/economics	1	2015	2015
pollution	density/count	superfund sites density	present		invertebrates	conservation	1	2021	2021
pollution	density/count	toxic release sites density 100m radius	present		invertebrates	conservation	1	2021	2021
pollution	density/count	wastewater discharge m^3	present	6	invertebrates	disturbance/habitat change	1	2018	2018
pollution	descriptive	andosol	present		herbaceous plants	disturbance/habitat change	1	2015	2015
pollution	descriptive	artificial illumination	present		mammals	disturbance/habitat change	1	2021	2021
pollution	descriptive	dump or fallow land	present		birds	conservation	1	2012	2012
pollution	descriptive	dumped materials	present		herbaceous plants	exploratory	1	2020	2020
pollution	descriptive	hydrocarbon fields saline aquifers	present		invertebrates	conservation	1	2014	2014
pollution	descriptive	inorganic fertilization	present		mammals	conflict/collisions	1	2020	2020
pollution	descriptive	light condition type	present		mammals	conflict/collisions	1	2013	2013
pollution	descriptive	pesticide neonicotinoid use	present		invertebrates	disturbance/habitat change	1	2020	2020
pollution	descriptive	pesticide risk class	present	1	invertebrates	exploratory	1	2017	2017
pollution	descriptive	pesticide soybean aphid chemical use	present		invertebrates	disturbance/habitat change	1	2020	2020
pollution	descriptive	pesticide use kg historic yr1990	present		invertebrates	conservation	1	2021	2021
pollution	descriptive	phosphorus total catchment	present		fish	reintroduction/restoration	1	2021	2021
pollution	descriptive	pollutants polychlorinated biphenyls	present		invertebrates	disturbance/habitat change	1	2019	2019
pollution	descriptive	pollutants polycyclic aromatic hydrocarbons	present		invertebrates	disturbance/habitat change	1	2019	2019

pollution	descriptive	pollution class	present	01; 06; 11; 13	mammals	conservation	1	2006	2006
pollution	descriptive	polyaromatic hydrocarbons high	present		invertebrates	food/economics	1	2015	2015
pollution	descriptive	polyaromatic hydrocarbons low	present		invertebrates	food/economics	1	2015	2015
pollution	descriptive	soil with brick rubble presence	present	15	herbaceous plants; invertebrates	conservation	1	2011	2011
pollution	distance	artificial illumination distance	present		mammals	conflict/collisions	1	2020	2020
pollution	distance	contaminated sites distance	present	6	mammals	exploratory	1	2015	2015
pollution	distance	pollution point source distance	present	06; 11; 13	herbaceous plants	invasions	1	2017	2017
pollution	distance	waste dumping distance	present	06; 12	invertebrates	human health/safety	1	2018	2018
pollution	index	herbicide pressure	present		herbaceous plants	exploratory	1	2013	2013
pollution	index	illegal poison risk index	present		birds	exploratory	1	2015	2015
pollution	index	insecticide application rate	present		invertebrates	exploratory	1	2020	2020
pollution	index	night light development index	present		mammals	exploratory	2	2020	2021
pollution	index	night light intensity	present		mammals; birds; reptiles; herbaceous plants; invertebrates	conservation; exploratory; invasions; disturbance/habitat change; human health/safety; reintroduction/restoration; conflict/collisions	24	2011	2021
pollution	index	ocean acidification	present	14	microorganisms; invertebrates	conservation; exploratory	2	2014	2020

pollution	index	pesticide application rate kg km2	present		invertebrates	conservation	1	2017	2017
pollution	index	pesticide risk index	present		invertebrates	disturbance/habitat change	1	2019	2019
pollution	index	polyaromatic hydrocarbons high-low ratio	present		invertebrates	food/economics	1	2015	2015
recreation/tourism	density/count	domestic dog density	present		mammals	conservation	1	2006	2006
recreation/tourism	density/count	garden percent	present		invertebrates	conservation	1	2017	2017
recreation/tourism	density/count	hunting area percent	present		mammals	conflict/collisions	1	2020	2020
recreation/tourism	density/count	parks percent 2500m radius	present		birds	invasions	1	2009	2009
recreation/tourism	density/count	parks percent 50m radius	present		birds	invasions	1	2009	2009
recreation/tourism	density/count	pet shop density	present		reptiles	invasions	1	2017	2017
recreation/tourism	density/count	recreational areas percent	present; future		invertebrates	exploratory	1	2021	2021
recreation/tourism	density/count	recreational parks percent	present		reptiles	conflict/collisions	1	2021	2021
recreation/tourism	density/count	tourism percent	present		reptiles	exploratory	1	2014	2014
recreation/tourism	density/count	tourist arrival yr2014 count	present		microorganisms	conservation	1	2020	2020
recreation/tourism	density/count	trails density	present		mammals	reintroduction/restoration	1	2018	2018
recreation/tourism	descriptive	artisanal fishing presence	present	14	microorganisms	conservation	1	2020	2020
recreation/tourism	descriptive	game guard station	present		mammals	invasions	1	2013	2013
recreation/tourism	descriptive	garden	present		invertebrates; mammals; birds	food/economics; invasions; exploratory	3	2012	2016
recreation/tourism	descriptive	golf courses	present		reptiles	disturbance/habitat change	1	2006	2006
recreation/tourism	descriptive	hiking trails presence	present		birds	exploratory	1	2017	2017
recreation/tourism	descriptive	hunting areas	present		mammals	disturbance/habitat change	1	2015	2015
recreation/tourism	descriptive	parks and gardens	present		birds	exploratory	1	2012	2012
recreation/tourism	descriptive	parks cemeteries golf courses	present		mammals	exploratory	1	2017	2017
recreation/tourism	descriptive	recreational areas	present		trees/shrubs	invasions	1	2018	2018
recreation/tourism	descriptive	scenic locations	present		mammals	conservation	1	2018	2018

recreation/tourism	descriptive	trails	present		mammals; birds	invasions; disturbance/habitat change; exploratory; conservation	6	2000	2020
recreation/tourism	descriptive	trails adjacent	present		herbaceous plants	invasions	1	2013	2013
recreation/tourism	distance	bike trails distance	present		mammals	reintroduction/restoration	1	2015	2015
recreation/tourism	distance	bike trails distance^2	present		mammals	reintroduction/restoration	1	2015	2015
recreation/tourism	distance	campground distance	present		invertebrates; birds	invasions; conservation	2	2016	2019
recreation/tourism	distance	fishing camp distance	present	14	mammals	disturbance/habitat change	1	2015	2015
recreation/tourism	distance	garden centers distance	present		amphibians; fish; invertebrates; reptiles	invasions	1	2019	2019
recreation/tourism	distance	garden distance	present		mammals; birds	conservation	2	2018	2020
recreation/tourism	distance	hiking and biking trails distance	present		mammals	conflict/collisions	1	2017	2017
recreation/tourism	distance	hiking trails distance	present		herbaceous plants	invasions	1	2021	2021
recreation/tourism	distance	hunter access point distance	present		mammals	disturbance/habitat change	1	2020	2020
recreation/tourism	distance	hunting zone distance	present		mammals	conservation	1	2014	2014
recreation/tourism	distance	linear recreation distance	present		mammals	food/economics	1	2019	2019
recreation/tourism	distance	park or garden distance	present		mammals	exploratory	1	2015	2015
recreation/tourism	distance	parks distance	present		birds	invasions	1	2009	2009
recreation/tourism	distance	plantations and gardens distance	present		birds	conflict/collisions	1	2020	2020
recreation/tourism	distance	recreational areas distance	present		herbaceous plants	invasions	1	2014	2014
recreation/tourism	distance	roads and trails distance	present		herbaceous plants	invasions	2	2015	2018
recreation/tourism	distance	roads or trails distance	present		birds	exploratory	1	2005	2005

recreation/tourism	distance	scenic location distance	present		mammals	reintroduction/restoration	1	2021	2021
recreation/tourism	distance	ski-lift and cableway distance	present		birds; mammals	reintroduction/restoration; conflict/collisions	2	2020	2021
recreation/tourism	distance	ski-resort distance	present		mammals	conservation	1	2014	2014
recreation/tourism	distance	ski lift distance	present		birds	disturbance/habitat change	1	2012	2012
recreation/tourism	distance	tourism site distance	present		mammals	exploratory	1	2021	2021
recreation/tourism	distance	trails distance	present		mammals; invertebrates; birds; herbaceous plants; trees/shrubs	conservation; invasions; reintroduction/restoration; disturbance/habitat change; food/economics	14	2014	2021
recreation/tourism	distance	trails secondary distance	present		mammals	disturbance/habitat change	1	2020	2020
recreation/tourism	distance	trails vehicular distance	present		mammals	reintroduction/restoration	1	2018	2018
recreation/tourism	index	motorized recreation intensity	present		mammals	food/economics	1	2019	2019
recreation/tourism	index	non-motorized recreation intensity	present		mammals	food/economics	1	2019	2019
recreation/tourism	index	trails index	present		herbaceous plants	exploratory	1	2018	2018
recreation/tourism	size	golf course size	present		invertebrates	exploratory	1	2017	2017
recreation/tourism	size	ski tracks and lifts length	present		herbaceous plants	invasions	1	2013	2013
socio-economic	density/count	agricultural areas workers number individuals	present		herbaceous plants	food/economics	1	2018	2018
socio-economic	density/count	citizens >75yrs percent 1500m radius	present		birds	conservation	1	2020	2020
socio-economic	density/count	citizens with higher education percent 1500m radius	present	4	birds	conservation	1	2020	2020
socio-economic	density/count	cleared vegetation percent 1km radius	present		mammals	disturbance/habitat change	1	2018	2018

socio-economic	density/count	commune income per citizen 1500m radius	present	1	birds	conservation	1	2020	2020
socio-economic	density/count	drug-related killings	present		herbaceous plants	conflict/collisions	1	2015	2015
socio-economic	density/count	education bachelors and above percent	present		trees/shrubs; mammals	invasions; conflict/collisions	2	2015	2018
socio-economic	density/count	education high school and below percent	present	4	trees/shrubs	invasions	1	2018	2018
socio-economic	density/count	farmers 50-65yrs old count	present		invertebrates	exploratory	1	2006	2006
socio-economic	density/count	household income	present	1	birds; mammals	exploratory; conflict/collisions	2	2015	2019
socio-economic	density/count	household income median	present	1	invertebrates; trees/shrubs; birds	invasions	3	2014	2019
socio-economic	density/count	household income per capita	present	1	herbaceous plants	invasions	1	2021	2021
socio-economic	density/count	household separate parcels used count	present		herbaceous plants	food/economics	1	2013	2013
socio-economic	density/count	households number per block	present		invertebrates	human health/safety	1	2017	2017
socio-economic	density/count	households white percent	present		trees/shrubs	invasions	1	2018	2018
socio-economic	density/count	human population below poverty line percent	present	01; 11; 15	invertebrates	human health/safety	1	2019	2019
socio-economic	density/count	human population centers count	present	11	birds	exploratory	1	2005	2005

socio-economic	density/count	human population density	past; present; future	11	birds; mammals; fish; trees/shrubs; invertebrates; amphibians; reptiles; herbaceous plants; microorganisms	invasions; reintroduction/restoration; disturbance/habitat change; exploratory; human health/safety; conservation; food/economics; conflict/collisions	183	2001	2021
socio-economic	density/count	human population density 100km from lake	present	11	fish	invasions	1	2016	2016
socio-economic	density/count	human population density 1km radiusreef	present	11	fish	disturbance/habitat change	1	2012	2012
socio-economic	density/count	human population density catchment	present	11	fish	reintroduction/restoration	1	2021	2021
socio-economic	density/count	human population density catchment 100m radius	present	11	invertebrates	conservation	1	2021	2021
socio-economic	density/count	human population density coefficient of variation	present	11	mammals	reintroduction/restoration	1	2021	2021
socio-economic	density/count	human population density interpolated	present	11	mammals	exploratory	1	2001	2001
socio-economic	density/count	human population density max	present; future	11	mammals	exploratory	1	2019	2019
socio-economic	density/count	human population density mean	present; future	11	mammals	exploratory	1	2019	2019
socio-economic	density/count	human population density mean 10km	present	11	birds	exploratory	1	2006	2006
socio-economic	density/count	human population density min	present; future	11	mammals	exploratory	1	2019	2019
socio-economic	density/count	human population density river 100m radius	present	11	invertebrates	conservation	1	2021	2021
socio-economic	density/count	human population density rural	present	11	mammals	conservation; invasions; food/economics	4	2013	2018

socio-economic	density/count	human population density rural historic	present	11	mammals	conservation	1	2013	2013
socio-economic	density/count	human population density sd	present; future	11	mammals	exploratory	1	2019	2019
socio-economic	density/count	human population density within 200km	present	11	fish	disturbance/habitat change	1	2012	2012
socio-economic	density/count	human population density yr2000	present	11	mammals	exploratory	1	2021	2021
socio-economic	density/count	human population growth	present	11	birds	conservation	1	2014	2014
socio-economic	density/count	human population over 65yrs percent	present	11	birds	invasions	1	2017	2017
socio-economic	density/count	human population under 14yrs percent	present	11	birds	invasions	1	2017	2017
socio-economic	density/count	industry density	present		mammals	disturbance/habitat change	1	2021	2021
socio-economic	density/count	inhabitants nearest village count	present		birds	conservation	1	2017	2017
socio-economic	density/count	livestock owners count	present		birds	conservation	1	2017	2017
socio-economic	density/count	median household income	present	1	invertebrates	human health/safety	1	2019	2019
socio-economic	density/count	personal income mean	present	1	birds	invasions	1	2017	2017
socio-economic	density/count	postal address forwards total count	present		invertebrates	invasions	1	2019	2019
socio-economic	density/count	profession farm forestry fishing percent	present	14; 15	trees/shrubs	invasions	1	2018	2018
socio-economic	density/count	property owners percent	present		trees/shrubs	invasions	1	2018	2018
socio-economic	density/count	renters percent	present		trees/shrubs	invasions	1	2018	2018
socio-economic	density/count	residents >65yrs percent	present		trees/shrubs	invasions	1	2018	2018
socio-economic	density/count	residents density	present		birds	exploratory	1	2015	2015
socio-economic	density/count	residents working in ag forestry fishing hunting mining percent	present	14; 15	mammals	conflict/collisions	1	2015	2015
socio-economic	density/count	retired people density	present		birds	invasions	1	2018	2018
socio-economic	density/count	rural workers per district count	present		trees/shrubs	food/economics	1	2016	2016
socio-economic	density/count	unemployment percent	present	8	trees/shrubs	invasions	1	2018	2018

socio-economic	density/count	unemployment percent 1500m radius	present	8	birds	conservation	1	2020	2020
socio-economic	descriptive	community associations presence	present	11	trees/shrubs	invasions	1	2018	2018
socio-economic	descriptive	country state name	present		birds	exploratory	1	2021	2021
socio-economic	descriptive	household	present		mammals	disturbance/habitat change	1	2016	2016
socio-economic	descriptive	human population centers	present	11	invertebrates	invasions	1	2020	2020
socio-economic	descriptive	human population distribution	present	11	trees/shrubs	food/economics	1	2019	2019
socio-economic	descriptive	land tenure	present		mammals	conflict/collisions	1	2013	2013
socio-economic	descriptive	land tenure class	present	1	mammals	disturbance/habitat change	1	2018	2018
socio-economic	descriptive	police presence	present		herbaceous plants	conflict/collisions	1	2015	2015
socio-economic	descriptive	residences basic needs unmet	present		invertebrates	human health/safety	1	2018	2018
socio-economic	descriptive	state name	present		birds	conservation	1	2016	2016
socio-economic	distance	community distance	present	11	mammals	food/economics	1	2019	2019
socio-economic	distance	healthcare distance	present	3	birds	invasions	1	2018	2018
socio-economic	distance	human population centers distance	present	11	fish; birds	disturbance/habitat change	2	2011	2012
socio-economic	distance	human population large distance	present	11	birds; mammals	conservation	1	2007	2007
socio-economic	distance	human population small distance	present	11	birds; mammals	conservation	1	2007	2007
socio-economic	distance	inhabitants distance 1km radius	present		birds	reintroduction/restoration	1	2009	2009
socio-economic	distance	inhabitants distance 2.5km radius	present		birds	reintroduction/restoration	1	2009	2009
socio-economic	distance	inhabitants distance 5km radius	present		birds	reintroduction/restoration	1	2009	2009
socio-economic	distance	large communities distance	present		birds	conservation	1	2020	2020
socio-economic	distance	major human population distance	present	11	mammals	disturbance/habitat change	1	2012	2012

socio-economic	index	community associations activeness	present	11	trees/shrubs	invasions	1	2018	2018
socio-economic	index	education index	present		invertebrates	human health/safety	1	2019	2019
socio-economic	index	gross domestic product	present		mammals; microorganisms; invertebrates	exploratory; conservation; invasions	4	2013	2020
socio-economic	index	gross domestic product national	present		fish; herbaceous plants; invertebrates; amphibians; birds; mammals; reptiles; trees/shrubs	invasions	1	2019	2019
socio-economic	index	gross domestic product percent invested in environmental programs	present		mammals	conservation	1	2013	2013
socio-economic	index	household movement from quarantined counties	present		invertebrates	invasions	1	2008	2008
socio-economic	index	household value yr2000	present		herbaceous plants	food/economics	1	2013	2013
socio-economic	index	human population change 1951-1991 percent	present	11	trees/shrubs	disturbance/habitat change	1	2015	2015
socio-economic	index	human population change business as usual	future	11	trees/shrubs	disturbance/habitat change	1	2015	2015
socio-economic	index	human population change static	future	11	trees/shrubs	disturbance/habitat change	1	2015	2015
socio-economic	index	human population density change yr1985-2004	present	11	mammals	disturbance/habitat change	1	2015	2015
socio-economic	index	human population density change yr2000-2020	present	11	invertebrates	invasions	1	2021	2021
socio-economic	index	human population diffusion	present	11	mammals	exploratory	1	2003	2003
socio-economic	index	human poverty	present	1	invertebrates	human health/safety	1	2017	2017

socio-economic	index	land value	present		trees/shrubs	invasions	1	2018	2018
socio-economic	index	landowner wealth	present		trees/shrubs	invasions	1	2018	2018
socio-economic	size	agricultural land area size used per household	present	02; 11; 15	herbaceous plants	food/economics	1	2013	2013
socio-economic	size	household size	present		herbaceous plants	food/economics	1	2013	2013
socio-economic	size	household size age 13-55	present		herbaceous plants	food/economics	1	2013	2013
socio-economic	size	household size migrants	present		herbaceous plants	food/economics	1	2013	2013
socio-economic	size	marijuana eradication area size	present		herbaceous plants	conflict/collisions	1	2015	2015
socio-economic	size	opium eradication area size	present		herbaceous plants	conflict/collisions	1	2015	2015
socio-economic	time	travel time <15min percent	present		trees/shrubs	invasions	1	2018	2018
transportation	density/count	airports percent	present		birds	reintroduction/restoration	1	2012	2012
transportation	density/count	airstrips percent	present		amphibians	conservation	1	2015	2015
transportation	density/count	boat launchcount	present		invertebrates	invasions	1	2021	2021
transportation	density/count	canal density	present		amphibians; fish; invertebrates; reptiles	invasions; exploratory; conservation	3	2010	2019
transportation	density/count	canals percent	present		amphibians	conservation	1	2015	2015
transportation	density/count	canals percent'	present		amphibians	conservation	1	2015	2015
transportation	density/count	communal roads density	present		mammals	conservation	1	2019	2019
transportation	density/count	dirt roads density	present		mammals	reintroduction/restoration	1	2018	2018
transportation	density/count	dirt roads major percent	present		birds	conservation	1	2016	2016
transportation	density/count	dirt roads minor percent	present		birds	conservation	1	2016	2016
transportation	density/count	field path percent	present		invertebrates	food/economics	1	2013	2013
transportation	density/count	highways density	past; present		mammals; herbaceous plants; birds	reintroduction/restoration; conflict/collisions; exploratory; conservation	9	2009	2020
transportation	density/count	highways interstate density	present		birds	conservation	1	2013	2013

transportation	density/count	linear features density	present	mammals	disturbance/habitat change; exploratory	3	2014	2021
transportation	density/count	major transportation percent	past; present	fish	conservation	1	2021	2021
transportation	density/count	railways density	present	birds; invertebrates; mammals; reptiles; herbaceous plants	reintroduction/restoration; invasions; exploratory	4	2008	2020
transportation	density/count	railways percent	present	mammals	exploratory	1	2013	2013
transportation	density/count	regional roads density	present	mammals	conflict/collisions	1	2019	2019
transportation	density/count	road-stream intersections count	present	fish	conservation	1	2013	2013
transportation	density/count	roads access count	present	mammals	disturbance/habitat change	1	2015	2015
transportation	density/count	roads and highways density	present	mammals	exploratory	1	2020	2020
transportation	density/count	roads and railways density	present	herbaceous plants	invasions	1	2021	2021
transportation	density/count	roads autonomic density	present	mammals	conservation	1	2016	2016
transportation	density/count	roads conventional density	present	mammals	exploratory	1	2014	2014
transportation	density/count	roads count	present	mammals; herbaceous plants	invasions	2	2012	2013
transportation	density/count	roads county density	present	mammals	conservation	1	2019	2019
transportation	density/count	roads crossing density	present	fish	reintroduction/restoration; conservation	3	2019	2021
transportation	density/count	roads crossings frequency	present	fish	conservation	1	2020	2020

transportation	density/count	roads density	past; present	mammals; birds; amphibians; invertebrates; herbaceous plants; reptiles; fish; microorganisms; trees/shrubs	conflict/collisions; reintroduction/restoration; disturbance/habitat change; exploratory; conservation; invasions; food/economics; human health/safety	89	2001	2021
transportation	density/count	roads density <7m	present	mammals	reintroduction/restoration	1	2018	2018
transportation	density/count	roads density >7m	present	mammals	reintroduction/restoration	1	2018	2018
transportation	density/count	roads density 1km radius	present	mammals	exploratory; conservation	4	2010	2021
transportation	density/count	roads density 4km radius	present	mammals	conservation	1	2020	2020
transportation	density/count	roads density mean	present	fish	conservation	1	2015	2015
transportation	density/count	roads density mean 150m radius	present	herbaceous plants	invasions	1	2015	2015
transportation	density/count	roads lake perimeter count	present	fish	invasions	1	2016	2016
transportation	density/count	roads local density	present	mammals	conflict/collisions; exploratory	2	2015	2019
transportation	density/count	roads low-use density	present	mammals	conservation	1	2012	2012
transportation	density/count	roads major and local density	present	mammals	exploratory	1	2015	2015
transportation	density/count	roads major density	present	mammals	conflict/collisions; conservation; reintroduction/restoration	3	2006	2020
transportation	density/count	roads major traffic volume	present	mammals	conflict/collisions	1	2020	2020
transportation	density/count	roads minor density	present	mammals	conflict/collisions; reintroduction/restoration	2	2006	2020
transportation	density/count	roads minor traffic volume	present	mammals	conflict/collisions	1	2020	2020
transportation	density/count	roads narrow percent	present	mammals	human health/safety	1	2014	2014
transportation	density/count	roads national density	present	mammals	conservation	2	2016	2019

transportation	density/count	roads paved density	present	mammals	conservation; reintroduction/restoration; conflict/collisions; exploratory	6	2008	2021
transportation	density/count	roads paved density 1km radius	present	birds	reintroduction/restoration	1	2009	2009
transportation	density/count	roads paved density 2.5km radius	present	birds	reintroduction/restoration	1	2009	2009
transportation	density/count	roads paved density 5km radius	present	birds	reintroduction/restoration	1	2009	2009
transportation	density/count	roads paved percent	present	birds; amphibians	conservation; exploratory	2	2015	2016
transportation	density/count	roads percent	present	birds; invertebrates; mammals; microorganisms; fish	invasions; conservation; exploratory; reintroduction/restoration; human health/safety; conflict/collisions	12	2007	2020
transportation	density/count	roads percent 10km radius	present	mammals	conservation	1	2013	2013
transportation	density/count	roads percent 1km radius	present	mammals	conservation	1	2013	2013
transportation	density/count	roads percent 20km radius	present	mammals	exploratory	1	2020	2020
transportation	density/count	roads percent 25m radius	present	birds	exploratory	1	2010	2010
transportation	density/count	roads percent 4km radius	present	mammals	conservation	1	2013	2013
transportation	density/count	roads percent 500m radius	present; future	amphibians	disturbance/habitat change	1	2013	2013
transportation	density/count	roads percent 7km radius	present	mammals	conservation	1	2013	2013
transportation	density/count	roads primary and secondary percent	present	birds	disturbance/habitat change	1	2021	2021
transportation	density/count	roads primary density	present	mammals	disturbance/habitat change; conflict/collisions; conservation; exploratory	4	2014	2020
transportation	density/count	roads secondary density	past; present	mammals; birds	conflict/collisions; conservation	4	2013	2019

transportation	density/count	roads stream crossing count	past		fish	exploratory	1	2016	2016
transportation	density/count	roads stream crossing density	present		fish; invertebrates	conservation	1	2018	2018
transportation	density/count	roads tertiary density	present		mammals	disturbance/habitat change	1	2020	2020
transportation	density/count	roads unpaved density	present		mammals	conservation	1	2014	2014
transportation	density/count	roads unpaved density 1km radius	present		birds	reintroduction/restoration	1	2009	2009
transportation	density/count	roads unpaved density 2.5km radius	present		birds	reintroduction/restoration	1	2009	2009
transportation	density/count	roads unpaved density 5km radius	present		birds	reintroduction/restoration	1	2009	2009
transportation	density/count	roads wide percent	present		mammals	human health/safety	1	2014	2014
transportation	density/count	shipping lines count	present		microorganisms	invasions	1	2021	2021
transportation	density/count	ships count	present		invertebrates	invasions	1	2013	2013
transportation	density/count	ships count natural log	present		microorganisms	invasions	1	2021	2021
transportation	density/count	ships docked at ports count	present		microorganisms	invasions	1	2021	2021
transportation	density/count	ships gross tonnage	present		microorganisms	invasions	1	2021	2021
transportation	density/count	ships gross tonnage docked at port	present		microorganisms	invasions	1	2021	2021
transportation	density/count	ships gross tonnage natural log	present		microorganisms	invasions	1	2021	2021
transportation	density/count	sports and leisure facilities percent	present		birds	reintroduction/restoration	1	2012	2012
transportation	density/count	state roads density	present		mammals	conflict/collisions	1	2019	2019
transportation	density/count	tracks density	present		birds	disturbance/habitat change	1	2004	2004
transportation	density/count	trade frog legs sum imports export	present	9	microorganisms	conservation	1	2013	2013
transportation	density/count	trade sum imports export	present	9	microorganisms	conservation	1	2013	2013
transportation	density/count	traffic density	present		mammals	conservation	1	2006	2006
transportation	density/count	traffic volume	present; future		mammals; invertebrates; birds; reptiles	conflict/collisions; food/economics; conservation; invasions	5	2018	2020

transportation	density/count	traffic volume 20mile radius	present		invertebrates	invasions	1	2019	2019
transportation	density/count	waterway density	present		birds	reintroduction/restoration	1	2021	2021
transportation	descriptive	boat launch presence	present		herbaceous plants	invasions	1	2014	2014
transportation	descriptive	canal presence	present		reptiles	invasions	1	2021	2021
transportation	descriptive	dirt roads	present		amphibians	conservation	1	2015	2015
transportation	descriptive	dirt roads major bare soil edges length	present	15	birds	conservation	1	2016	2016
transportation	descriptive	dirt roads major grass edges length	present		birds	conservation	1	2016	2016
transportation	descriptive	dirt roads major riparian edges length	present		birds	conservation	1	2016	2016
transportation	descriptive	dirt roads major shrub edges length	present		birds	conservation	1	2016	2016
transportation	descriptive	dirt roads major tree edges length	present	15	birds	conservation	1	2016	2016
transportation	descriptive	dirt roads minor bare soil edges length	present	15	birds	conservation	1	2016	2016
transportation	descriptive	dirt roads minor grass edges length	present		birds	conservation	1	2016	2016
transportation	descriptive	dirt roads minor riparian edges length	present		birds	conservation	1	2016	2016
transportation	descriptive	dirt roads minor shrub edges length	present		birds	conservation	1	2016	2016
transportation	descriptive	dirt roads minor tree edges length	present	15	birds	conservation	1	2016	2016
transportation	descriptive	field path	present		birds	conservation	1	2012	2012
transportation	descriptive	field path 25m radius	present		invertebrates	food/economics	1	2013	2013
transportation	descriptive	field path 50m radius	present		invertebrates	food/economics	1	2013	2013
transportation	descriptive	highways	present		invertebrates; birds	invasions	2	2016	2020
transportation	descriptive	linear features	present		mammals	conservation	1	2021	2021
transportation	descriptive	paved roads	present		amphibians	conservation	1	2015	2015
transportation	descriptive	ports harbors shipping areas	present		invertebrates	conservation	1	2014	2014

transportation	descriptive	railways	present		reptiles; herbaceous plants; mammals	invasions	3	2014	2018
transportation	descriptive	railways verge	present		mammals	invasions	1	2014	2014
transportation	descriptive	roads	present		reptiles; mammals; herbaceous plants; invertebrates	conservation; exploratory; invasions; disturbance/habitat change; human health/safety	16	2007	2021
transportation	descriptive	roads adjacent	present		herbaceous plants	invasions	1	2013	2013
transportation	descriptive	roads main	present		mammals	disturbance/habitat change	1	2014	2014
transportation	descriptive	roads paved bare soil edges length	present	15	birds	conservation	1	2016	2016
transportation	descriptive	roads paved edge	present		invertebrates	exploratory	1	2020	2020
transportation	descriptive	roads paved grass edges length	present		birds	conservation	1	2016	2016
transportation	descriptive	roads paved patch	present		invertebrates	exploratory	1	2020	2020
transportation	descriptive	roads paved radius	present		invertebrates	exploratory	1	2020	2020
transportation	descriptive	roads paved riparian edges length	present		birds	conservation	1	2016	2016
transportation	descriptive	roads paved shrub edges length	present		birds	conservation	1	2016	2016
transportation	descriptive	roads paved tree edges length	present	15	birds	conservation	1	2016	2016
transportation	descriptive	roads primary	present		mammals	conservation	1	2012	2012
transportation	descriptive	roads primative adjacent	present		herbaceous plants	invasions	1	2013	2013
transportation	descriptive	roads secondary	present		mammals	conservation	2	2012	2015
transportation	descriptive	roads type	present		reptiles; mammals	conflict/collisions; invasions; disturbance/habitat change	4	2013	2021

transportation	descriptive	roads unpaved 10m radius	present	invertebrates	exploratory	1	2020	2020
transportation	descriptive	roads unpaved edge	present	invertebrates	exploratory	1	2020	2020
transportation	descriptive	roads unpaved patch	present	invertebrates	exploratory	1	2020	2020
transportation	descriptive	roads verge	present	mammals	invasions	1	2014	2014
transportation	descriptive	roads winter	present	mammals	disturbance/habitat change	1	2014	2014
transportation	descriptive	seaports	present	invertebrates	invasions	1	2020	2020
transportation	descriptive	ship mooring latitude	present	mammals	disturbance/habitat change	1	2021	2021
transportation	descriptive	ship mooring longitude	present	mammals	disturbance/habitat change	1	2021	2021
transportation	descriptive	shipping	present	invertebrates	invasions	1	2013	2013
transportation	descriptive	shipwrecks	present	invertebrates	conservation	1	2014	2014
transportation	descriptive	sports 10m radius	present	invertebrates	exploratory	1	2020	2020
transportation	descriptive	tracks	present	mammals	invasions	1	2014	2014
transportation	descriptive	transportation	present	reptiles	disturbance/habitat change	1	2006	2006
transportation	descriptive	transportation presence	present	trees/shrubs	invasions	1	2018	2018
transportation	distance	airports distance	present	amphibians; fish; herbaceous plants; invertebrates; microorganisms; birds; mammals; reptiles; trees/shrubs	invasions	1	2016	2016
transportation	distance	aviation structure distance	present	birds	conservation	1	2016	2016

transportation	distance	boat launch distance	present		invertebrates; mammals; herbaceous plants; amphibians; fish; reptiles	invasions; conservation; reintroduction/restoration	4	2019	2021
transportation	distance	commercial ports distance	present		fish; herbaceous plants; invertebrates; amphibians; birds; mammals; reptiles; trees/shrubs	invasions	1	2019	2019
transportation	distance	commercial ports sailing distance	present		microorganisms	invasions	1	2021	2021
transportation	distance	county highways distance	present		birds	conservation	1	2021	2021
transportation	distance	dirt roads major distance	present		birds	conservation	1	2016	2016
transportation	distance	dirt roads minor distance	present		birds	conservation	1	2016	2016
transportation	distance	federal aviation administration structure distance	present		birds	exploratory	1	2021	2021
transportation	distance	forest and country roads distance	present	15	mammals	exploratory	1	2011	2011
transportation	distance	forest roads distance	present	15	herbaceous plants	invasions	1	2016	2016
transportation	distance	highway distance	present		birds; mammals	conservation; exploratory	2	2016	2021

transportation	distance	highways distance	present		invertebrates; birds; mammals; fish; herbaceous plants; amphibians; reptiles; trees/shrubs	invasions; exploratory; conservation; conflict/collisions; disturbance/habitat change; food/economics; reintroduction/restoration	23	2006	2021
transportation	distance	improved roads distance	present		mammals	exploratory	1	2007	2007
transportation	distance	linear features distance	present		birds	conservation	1	2016	2016
transportation	distance	major ports sailing distance	present		microorganisms	invasions	1	2021	2021
transportation	distance	merchant shipping routes distance	present		mammals	conservation	1	2016	2016
transportation	distance	navigable water distance	present	6	birds; mammals; reptiles	exploratory	1	2015	2015
transportation	distance	permanent waterway distance	present		mammals	conservation	1	2020	2020
transportation	distance	ports distance	present		invertebrates; amphibians; fish; reptiles; herbaceous plants; microorganisms; birds; mammals; trees/shrubs	invasions; food/economics	6	2013	2021
transportation	distance	ports distance index	present		invertebrates	invasions	1	2015	2015
transportation	distance	provincial highways distance	present		birds	conservation	1	2021	2021
transportation	distance	public roads distance mean	present		mammals	conservation	1	2006	2006

transportation	distance	railways distance	past; present		birds; mammals; invertebrates; reptiles; amphibians; trees/shrubs; herbaceous plants	conservation; reintroduction/restoration; human health/safety; exploratory; invasions; food/economics	16	2013	2021
transportation	distance	road to scrub distance	present		mammals	conflict/collisions	1	2020	2020
transportation	distance	roads and highways distance	present		mammals	exploratory	1	2015	2015
transportation	distance	roads and railways distance	present		birds; mammals	reintroduction/restoration; conflict/collisions; disturbance/habitat change; conservation	4	2010	2021
transportation	distance	roads construction distance	present		mammals	disturbance/habitat change	1	2019	2019
transportation	distance	roads crossing proximity	present		fish	conservation	1	2019	2019
transportation	distance	roads distance	past; present; future		mammals; birds; herbaceous plants; reptiles; invertebrates; trees/shrubs; amphibians; fish; microorganisms	conflict/collisions; disturbance/habitat change; conservation; invasions; reintroduction/restoration; exploratory; food/economics; human health/safety	225	2000	2021
transportation	distance	roads distance class	present	1	herbaceous plants	invasions	1	2015	2015
transportation	distance	roads distance^2	present		mammals	reintroduction/restoration	1	2015	2015
transportation	distance	roads highways railways distance	present		mammals	exploratory	1	2020	2020
transportation	distance	roads large distance	present		herbaceous plants	invasions	1	2014	2014

transportation	distance	roads local distance	present	mammals	disturbance/habitat change	1	2016	2016
transportation	distance	roads main distance	present	mammals; birds; herbaceous plants	conservation; conflict/collisions; invasions; disturbance/habitat change; reintroduction/restoration; exploratory	11	2010	2020
transportation	distance	roads main or highway distance	present	birds	conservation	1	2000	2000
transportation	distance	roads major and local distance	present	mammals	exploratory	1	2015	2015
transportation	distance	roads major distance	present	mammals; amphibians; herbaceous plants; birds	conservation; disturbance/habitat change; conflict/collisions; invasions; exploratory	10	2009	2021
transportation	distance	roads minor distance	present	mammals	conflict/collisions; disturbance/habitat change	2	2015	2020
transportation	distance	roads national distance	present	birds; mammals	conservation	2	2007	2017
transportation	distance	roads other distance	present	mammals	reintroduction/restoration	1	2013	2013
transportation	distance	roads paved distance	present	amphibians; trees/shrubs; mammals; birds; reptiles	disturbance/habitat change; invasions; conservation; exploratory; reintroduction/restoration	18	2003	2021
transportation	distance	roads primary distance	present	mammals; reptiles; amphibians; trees/shrubs; birds	reintroduction/restoration; conservation; exploratory; invasions; disturbance/habitat change	13	2004	2021
transportation	distance	roads provincial distance	present	birds	conservation	1	2017	2017

transportation	distance	roads secondary distance	past; present	mammals; birds; amphibians; trees/shrubs	conflict/collisions; reintroduction/restoration; conservation; exploratory; disturbance/habitat change	16	2004	2021
transportation	distance	roads small distance	present	birds; herbaceous plants	invasions	2	2014	2018
transportation	distance	roads tertiary distance	present	mammals	exploratory	2	2009	2018
transportation	distance	roads unpaved distance	present	trees/shrubs; mammals; birds	invasions; conservation; disturbance/habitat change; reintroduction/restoration; exploratory	9	2005	2021
transportation	distance	roadside rest area distance	present	birds	disturbance/habitat change	1	2018	2018
transportation	distance	rough roads paved distance	present	birds	disturbance/habitat change	1	2004	2004
transportation	distance	still watercourses distance	present	birds	conflict/collisions	1	2020	2020
transportation	distance	tracks distance	present	mammals; birds	disturbance/habitat change	2	2004	2010
transportation	distance	transportation distance	present	invertebrates; herbaceous plants	disturbance/habitat change; invasions	2	2014	2018
transportation	distance	transportation facilities distance	present	invertebrates	invasions	1	2019	2019
transportation	distance	transportation rest stops distance	present	invertebrates	invasions	1	2019	2019
transportation	distance	transportation weigh stations distance	present	invertebrates	invasions	1	2019	2019
transportation	distance	transports network distance	present	mammals	disturbance/habitat change	1	2019	2019

transportation	distance	transports network main distance	present		mammals	disturbance/habitat change	1	2019	2019
transportation	index	boat traffic	present		mammals	exploratory	1	2014	2014
transportation	index	roads accessibility	present		invertebrates	invasions	1	2008	2008
transportation	index	roads traffic index	present		birds	exploratory	1	2007	2007
transportation	index	shipping intensity	present		microorganisms; birds	conservation; exploratory	2	2017	2020
transportation	index	traffic annual daily	past; present		birds	disturbance/habitat change	1	2017	2017
transportation	index	traffic annual daily mean	present		invertebrates	human health/safety	1	2021	2021
transportation	index	vehicle and pedestrian transit level	present		mammals	disturbance/habitat change	1	2015	2015
transportation	size	canals length	present		herbaceous plants	invasions	1	2013	2013
transportation	size	highways length	past; present		herbaceous plants	human health/safety	2	2013	2018
transportation	size	highways length total	present		trees/shrubs	invasions	1	2021	2021
transportation	size	industrial commercial transportation area	present		herbaceous plants	invasions	1	2017	2017
transportation	size	railways length	past; present		herbaceous plants; birds; invertebrates	human health/safety; conservation; food/economics; invasions	5	2010	2018
transportation	size	roads and highways length	present		birds	exploratory	1	2009	2009
transportation	size	roads area size	present		birds; mammals	exploratory	1	2018	2018
transportation	size	roads forest residential length	present	15	herbaceous plants	invasions	1	2014	2014
transportation	size	roads large volume length	present		mammals	conflict/collisions	1	2010	2010
transportation	size	roads length	present		invertebrates; birds; fish; trees/shrubs; herbaceous plants; mammals	invasions; conservation; exploratory; reintroduction/restoration	14	2006	2021

transportation	size	roads length 1km radius	present	herbaceous plants	invasions	1	2019	2019
transportation	size	roads length density	present	invertebrates	food/economics	1	2014	2014
transportation	size	roads length mean	present	mammals	exploratory	1	2017	2017
transportation	size	roads length mean 10km	present	birds	exploratory	1	2006	2006
transportation	size	roads length paved	present	birds	conservation	1	2017	2017
transportation	size	roads length sum	present	trees/shrubs	invasions; disturbance/habitat change	2	2015	2021
transportation	size	roads main length	present	birds	disturbance/habitat change	1	2012	2012
transportation	size	roads medium volume length	present	mammals	conflict/collisions	1	2010	2010
transportation	size	roads paved length	present	birds	exploratory	1	2005	2005
transportation	size	roads sealed length	present	mammals	conservation	1	2004	2004
transportation	size	roads small volume length	present	mammals	conflict/collisions	1	2010	2010
transportation	size	roads unpaved length	present	birds	conservation; exploratory	3	2005	2017
transportation	size	roads unsealed length	present	mammals	conservation	1	2004	2004
transportation	size	roads width	present	mammals	disturbance/habitat change	1	2015	2015
transportation	size	roads width category	present	reptiles	conflict/collisions	1	2021	2021
