# Supplementary material for: Gaps and opportunities in modelling human influence on species distributions in the Anthropocene
Source: Nat Ecol Evol. 2024 Jun 12;8(7):1365–77. doi: 10.1038/s41559-024-02435-3 (PMC11239511; doi:10.1038/s41559-024-02435-3)
Supplement: Supplementary file 2 — Reporting Summary [file 41559_2024_2435_MOESM2_ESM.pdf]

nature portfolio

Corresponding author(s): Veronica F. Frans

Last updated by author(s): 4/18/2024

Reporting Summary

Nature Portfolio wishes to improve the reproducibility of the work that we publish. This form provides structure for consistency and transparency in reporting. For further information on Nature Portfolio policies, see our Editorial Policies and the Editorial Policy Checklist.

Statistics

For all statistical analyses, confirm that the following items are present in the figure legend, table legend, main text, or Methods section.

n/a	Confirmed	
☐	☒	The exact sample size (*n*) for each experimental group/condition, given as a discrete number and unit of measurement
☐	☒	A statement on whether measurements were taken from distinct samples or whether the same sample was measured repeatedly
☒	☐	The statistical test(s) used AND whether they are one- or two-sided<br>*Only common tests should be described solely by name; describe more complex techniques in the Methods section.*
☒	☐	A description of all covariates tested
☒	☐	A description of any assumptions or corrections, such as tests of normality and adjustment for multiple comparisons
☐	☒	A full description of the statistical parameters including central tendency (e.g. means) or other basic estimates (e.g. regression coefficient) AND variation (e.g. standard deviation) or associated estimates of uncertainty (e.g. confidence intervals)
☒	☐	For null hypothesis testing, the test statistic (e.g. *F*, *t*, *r*) with confidence intervals, effect sizes, degrees of freedom and *P* value noted<br>*Give P values as exact values whenever suitable.*
☒	☐	For Bayesian analysis, information on the choice of priors and Markov chain Monte Carlo settings
☒	☐	For hierarchical and complex designs, identification of the appropriate level for tests and full reporting of outcomes
☒	☐	Estimates of effect sizes (e.g. Cohen's *d*, Pearson's *r*), indicating how they were calculated

Our web collection on statistics for biologists contains articles on many of the points above.

Software and code

Policy information about availability of computer code

Data collection	The data for the articles collected from Web of Science are available on Figshare (https://doi.org/10.6084/m9.figshare.24225316).
Data analysis	All data were analyzed using R version 4.3.0. All code for this study are publicly accessible on GitHub (https://github.com/vffrans/Human_influence_SDMs) and Figshare (https://doi.org/10.6084/m9.figshare.24225316).

For manuscripts utilizing custom algorithms or software that are central to the research but not yet described in published literature, software must be made available to editors and reviewers. We strongly encourage code deposition in a community repository (e.g. GitHub). See the Nature Portfolio guidelines for submitting code & software for further information.

Data

Policy information about availability of data

All manuscripts must include a data availability statement. This statement should provide the following information, where applicable:

- Accession codes, unique identifiers, or web links for publicly available datasets
- A description of any restrictions on data availability
- For clinical datasets or third party data, please ensure that the statement adheres to our policy

The datasets developed from this study are in Supplementary Tables 5 and 6. They are also publicly accessible on the Figshare repository (https://doi.org/10.6084/m9.figshare.24225316).

Human research participants

Policy information about studies involving human research participants and Sex and Gender in Research.

Reporting on sex and gender	not collected
Population characteristics	*Describe the covariate-relevant population characteristics of the human research participants (e.g. age, genotypic information, past and current diagnosis and treatment categories). If you filled out the behavioural & social sciences study design questions and have nothing to add here, write "See above."*
Recruitment	*Describe how participants were recruited. Outline any potential self-selection bias or other biases that may be present and how these are likely to impact results.*
Ethics oversight	*Identify the organization(s) that approved the study protocol.*

Note that full information on the approval of the study protocol must also be provided in the manuscript.

Field-specific reporting

Please select the one below that is the best fit for your research. If you are not sure, read the appropriate sections before making your selection.

☐ Life sciences ☐ Behavioural & social sciences ☒ Ecological, evolutionary & environmental sciences

For a reference copy of the document with all sections, see nature.com/documents/nr-reporting-summary-flat.pdf

Ecological, evolutionary & environmental sciences study design

All studies must disclose on these points even when the disclosure is negative.

Study description	This is a systematic review of articles on species distribution modeling (SDMs). We assessed whether articles published up to 2021 included human predictors in their models, and summarized those' articles procedures.
Research sample	Using Web of Science, we searched for all published articles up to December 31, 2021 that used the following terms in their titles, keywords, or abstracts: TS=(("species distribution model*" OR "environmental niche model*" OR "species niche model*" OR "bioclimatic niche model*" OR "habitat suitability model*" OR "ecological niche model*" OR "habitat model*")) AND DT=(Article) AND PY=(1900-2021)). This yielded 12,854 articles for the abstract screening step.
Sampling strategy	We screened all 12,854 article abstracts, searching for abstracts that indicate some acknowledgment of human influence on species' distributions. We manually screened ~300 abstracts at a time, added human-related terms found in those abstracts to a text-mining dictionary string, and then searched along the entire pool of abstracts to accept articles based on the updated terms. We repeated this for 28 iterations, allowing us to manually screen all rejected article abstracts (n=7,506), manually accept 551 article abstracts, automatically accept 4,626 article abstracts from the 477 terms added to the search, and manually review a total of 5,177 full articles and their supplementary materials (see PRISMA framework in Extended Data Fig. 1).
Data collection	All data were collected by the corresponding author, Veronica F. Frans. The 5,177 full articles that were accepted in the abstract screening step were manually downloaded based on a web search using each article's DOI or title. The supplementary materials of these articles were also collected. Fourteen accepted articles were not available. The full articles were then reviewed to see whether human predictors were used in species distribution models. This led to 1,429 eligible articles for our analysis and summary. We summarized the 1,429 articles by gathering the general focus (or, aim) of the study (as stated by the authors in the abstract or introduction), spatial scale of the study area, study area countries, the study's time frame (past, present and/or future SDM training and projection), the time frame represented by human predictors (including simulated scenarios across time), the taxa studied, study domain (terrestrial, marine, or freshwater habitat type), and SDM algorithms. For each article, we also listed the human predictors' names and the total numbers of environmental predictors used in the SDMs. We provide a description of these data in Supplementary Table 2.
Timing and spatial scale	We conducted the Web of Science search on September 14, 2022. The articles were collected from the year 1900 to 2021, but our analysis was restricted to articles published between 2000 and 2021. We did not limit the geographic coverage of our study.
Data exclusions	In the abstract screening step, we did not accept articles that did not mention human influence on species distributions in the abstract. Of the 5,177 full articles that we read, we did not accept articles that did not use human predictors within species distribution models.
Reproducibility	We maximized the reproducibility of our work by doing all abstract screening and data cleanup and analysis in R. The only portion of our work that cannot be automated is the collection of the raw data for each of the 1,429 accepted articles. These data were typed up in Excel while reading the articles. The PDFs of these articles were highlighted and annotated, and cannot be distributed due to copyright issues with article publications. However, all raw data corrections from the Excel spreadsheet were done in code in R. This project has 5 R scripts, which are accessible on GitHub (https://github.com/vffrans/Human_influence_SDMs) and Figshare (https://doi.org/10.6084/m9.figshare.24225316).

Randomization	We did not do randomization in our study. We were able to summarize all 1,429 accepted articles for our review and analysis.
Blinding	We did not do any blinding procedures in our study, since it was systematic review.

Did the study involve field work? ☐ Yes ☒ No

Reporting for specific materials, systems and methods

We require information from authors about some types of materials, experimental systems and methods used in many studies. Here, indicate whether each material, system or method listed is relevant to your study. If you are not sure if a list item applies to your research, read the appropriate section before selecting a response.

Materials & experimental systems

n/a	Involved in the study
☒	☐ Antibodies
☒	☐ Eukaryotic cell lines
☒	☐ Palaeontology and archaeology
☒	☐ Animals and other organisms
☒	☐ Clinical data
☒	☐ Dual use research of concern

Methods

n/a	Involved in the study
☒	☐ ChIP-seq
☒	☐ Flow cytometry
☒	☐ MRI-based neuroimaging
